# Multicomponent Synthesis of 3‑Thiazolines Using a Modified Asinger-Type Reaction

**DOI:** 10.1021/acs.joc.5c00563

**Published:** 2025-06-03

**Authors:** Vincent R.A.M. Reinartz, Jordy M. Saya, Darya Hadavi, Jules A.W. Harings, Romano V.A. Orrù

**Affiliations:** † Aachen Maastricht Institute for Biobased Materials (AMIBM), 5211 Maastricht University, Urmonderbaan 22, Geleen 6167RD, Netherlands; ‡ Maastricht MultiModal Molecular Imaging Institute (M4I), Maastricht University, Universiteitssingel 50, Geleen 6167RD, Netherlands

## Abstract

The classical Asinger reaction is a four-component process combining sulfur, ammonia, and oxo compounds to access 3-thiazolines, but its efficiency can be hampered by in situ ammonia-derived imine formation. We report a one-pot, two-step modification using preformed trimethylsilyl-imines to efficiently yield the desired 3-thiazolines. This method tolerates a wide range of aromatic aldehydes and α-halocarbonyl compounds under microwave irradiation. Benzothiazole-3-thiazoline products undergo bond isomerization to 2-thiazolines in HFIP under elevated microwave conditions, facilitating the synthesis of (±)-firefly luciferin and related derivatives.

## Graphical abstract

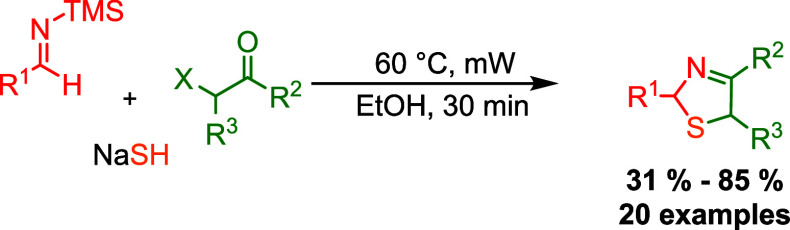

The Asinger reaction, first described by Friedrich Asinger in 1956, represents a powerful multicomponent approach for the synthesis of 3-thiazolines.^1,2^ These heterocycles are found in a wide range of bioactive compounds exhibiting antitubercular,^3^ antimicrobial,^4^ and anti-inflammatory^5,6^ properties (Scheme 1). As a multicomponent reaction (MCR), the Asinger reaction exemplifies the power of MCRs to rapidly construct structurally complex and diverse scaffolds from simple starting materials.^7–9^

**Scheme 1:**
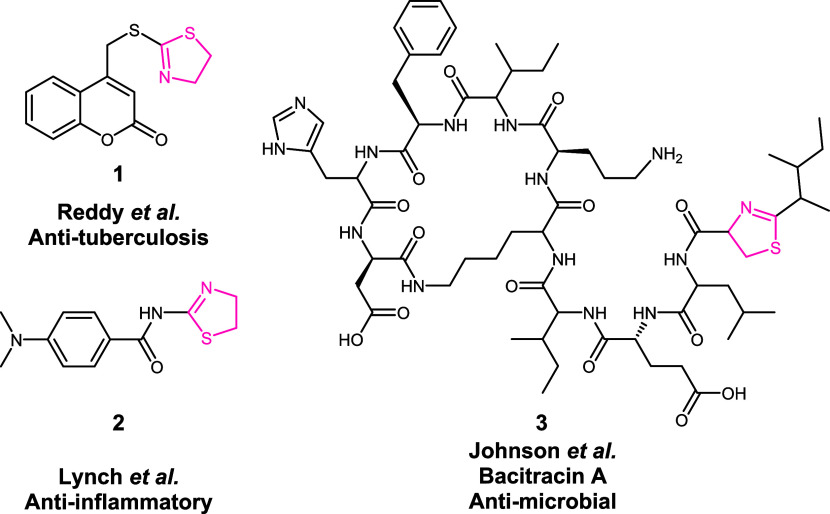
Various Bioactive Thiazoline-Bearing Compounds

The classical method employs elemental sulfur, gaseous ammonia, and two ketones or aldehydes to generate 3-thiazoline heterocycles.^1,10–14^ Although the Asinger four-component reaction (A-4CR) is generally efficient when using two (or more) equivalents of the same ketone/aldehyde, yields drop significantly with aromatic variants.^2^ Greater control and often improved yields can be achieved by preforming α-sulfanyl ketones/aldehydes, which act as intermediates in the A-4CR.^2^

In an attempt to complete a total synthesis of firefly luciferin (**8**) and analogues,^15,16^ the limitations of the A-4CR (and its α-sulfanyl variants) became clear. Therefore, this study aimed to harness the A-4CR’s capacity to rapidly generate a diverse 3-thiazoline library (**7**), followed by isomerization to 2-thiazolines and deprotection to yield a series of luciferin analogues (Scheme 2).

**Scheme 2:**
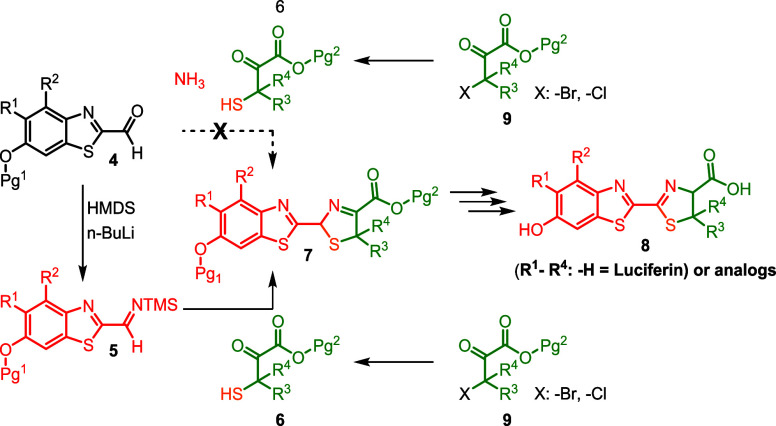
Conceptual Synthesis of luciferin (and analogs) Using the A-4CR

Attempts to combine benzothiazole aldehyde (**4**), ammonia, and ethyl 3-mercapto-2-oxopropanoates (**6**) to synthesize corresponding 3-thiazoline **7** were unsuccessful, despite extensive screening of reaction conditions. Less electrophilic aldehydes (e.g., aromatic variants) are known to be poorly tolerated in the A-4CR.^2,17^ A recently reported microwave-assisted protocol intended to overcome this limitation^18^ also failed to give the desired 3-thiazoline **7**.

The main reason for the above sluggish 3-thiazoline synthesis using the classical A-4CR approaches (Scheme 3a,b) lies in the *in situ* imine formation, which is difficult when combining ammonia and aromatic aldehydes.^19^ To overcome this intrinsic limitation in the A-4CR toward 3-thiazolines, we envisioned a one-pot, stepwise modification. This involves using trimethylsilyl (TMS)-imines as preformed surrogates for imines derived from aldehydes and ammonia (Scheme 3c).

**Scheme 3:**
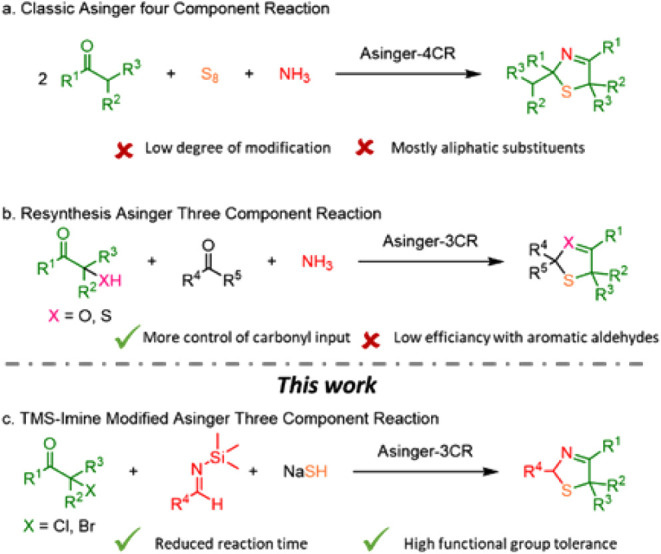
Different Types of Asinger MCRs

In this work, we report the results of our studies and show that our modified A-3CR reaction can indeed be used to access luciferin in a short and efficient synthetic sequence. We started our explorations to develop a modified Asinger reaction for 3-thiazolines by combining chloroacetone (**9a**) and NaSH to form α-sulfanyl-ketone (**6a**) and was cyclized *in situ* with *N*-trimethylsilylbenzaldimine (**5a**). This one-pot, two-step procedure indeed provided the 3-thiazoline **7a** in yields comparable to earlier reports via the classical A-4CR (Table 1).^14,18,20^ We then evaluated different solvents (entries 1–4). Protic polar solvents EtOH and HFIP emerged as the most effective, while aprotic polar solvents such as DCM and MeCN gave lower yields. Although HFIP gave slightly higher yields, we continued optimization with EtOH due to its ecofriendliness and lower toxicity.

**Table 1:**
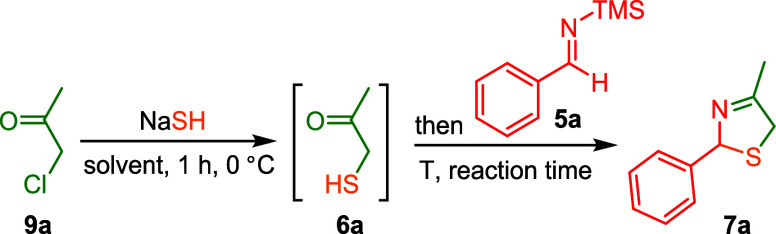
Reaction Optimization of the Three-Component Reaction Using α-Chloroacetone^a^ ^,^ ^b^

Entry	Solvent	*c* (M)	*T* (°C)	*t* (h)	N_2_	Yield (%)
1	EtOH	0.1	40	18	-	56
2	HFIP	0.1	40	18	-	58
3	DCM	0.1	40	18	-	35
4	MeCN	0.1	40	18	-	37
5^c^	EtOH	0.1	40	18	-	51
6	EtOH	0.2	40	18	-	46
7	EtOH	0.5	40	18	-	35
8	EtOH	0.1	40	18	-	56
9	EtOH	0.1	60	18	-	58
10	EtOH	0.1	60	3	-	70
11^d^	EtOH	0.1	60	0.5	-	63
12	EtOH	0.1	60	3	N_2_	78
**13** ^d^ ^e^	**EtOH**	**0.1**	**60**	**0.5**	**N**_**2**_	**92**

a Standard conditions: 0.34 mmol scale of **5a**.

b Yield determined by ^1^H NMR analysis 2,5-dimethylfuran as the internal standard.

c 2 equiv **9a** and NaSH.

d Microwave irradiation as the heating source.

e Ethanol predried using 4 Å MS.

Notably, increasing the stoichiometry of **6a** and the overall concentration resulted in decreased yields (entries 5–7). Further optimization of the yield of **7a** was achieved using a temperature of 60 °C (entries 8, 9) over 18 h. The reaction time could also be shortened to 3 h at 60 °C, with improved yield (entry 10). The most significant improvement was achieved by employing the above conditions under a nitrogen atmosphere, giving a 78% yield (entry 12). Comparable results were obtained under microwave irradiation, accomplishing the reaction in just 30 min (entry 11). Finally, using ethanol dried with 4 Å MS led to a satisfying 92% yield (entry 13). Although water is generally tolerated in the conventional A-4CR due to its dynamic equilibrium, in our variant, water in initial stages of the reaction can lead to the irreversible hydrolysis of imines **5a**–**o**, thus lowering the overall yield of 3-thiazolines

We next turned our attention to the scope of our modified Asinger reaction. First, we evaluated a set of TMS-imines (**5a**–**o**; preparation details in Supporting Information)^21^ for their ability to trap the *in situ*-formed α-sulfanyl-ketone (**6a**) under the optimized conditions. The reaction tolerated a broad range of functionalized phenyl substituents, affording the desired 3-thiazoline product (**7a**–**p**) in yields of 31–85% (Scheme 4). Although the introduction of alkyl substituents at the 2,6-positions introduces considerable steric bulk, the use of **5b** in our modified Asinger protocol gave the highest yield (**7b**). A notable trend emerged when electron-donating −OMe substituents (**7d**–**f**) were gradually placed closer to the reactive imine center, decreasing the yields. The opposite effect was observed when electron-withdrawing bromines were situated closer to the reactive center (**7i,j**). This trend in electronic properties was in line with the more moderate yield observed when the 4-CF_3_ substituted TMS-imine (**5g**) was employed, which gave 3-thiazoline (**7g**).

**Scheme 4:**
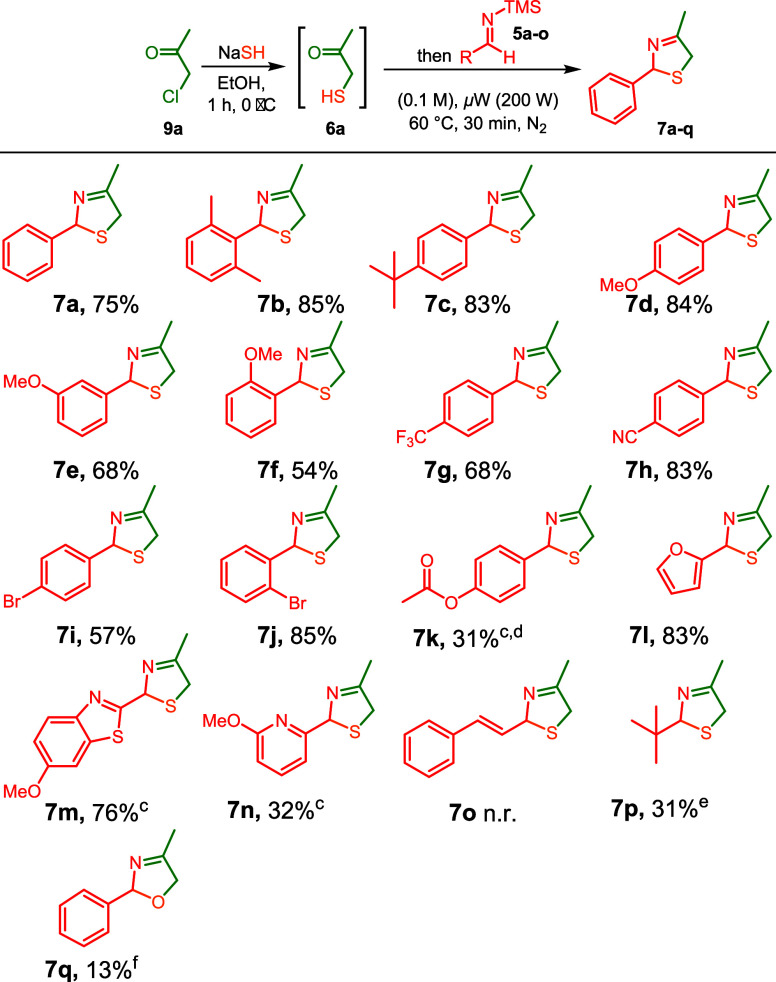
TMS-Imine Scope of the 3-Thiazoline Three-Component Reaction^a^ ^b^ ^c^ ^d^ ^e^ ^f^

a Standard conditions: NaSH (1 mmol) and **9a** (1 mmol) in EtOH at 0 °C, 1 h. Next, TMS-imine (0.67 mmol) microwave, 60 °C, 30 min under nitrogen.
b Isolated yields.
c Imine prepared *in situ*.
d Free alcohol group protected with Ac_2_O before isolation.
e Isolated by bulb-to-bulb distillation on 9.5 mmol scale.
f Hydroxyacetone used instead of **6a**.

In contrast, 4-CN-substituted **7h** was obtained in significantly higher yield. The relative stability of the TMS-imines that are used accounts for the observed trend. Subsequently, also the 4-OH TMS-imine derivative **5k** was subjected to our Asinger-type reaction. We found that even free −OH was tolerated, although the corresponding 3-thiazoline formed in a moderate yield of 30%. We slightly adapted our protocol, generating TMS-imine **5k** in situ followed by quenching excess LiHMDS (Section S4.2). Furthermore, *in situ* postmodification acyl analogue (**7k**) was necessary, as purification of the initially formed 3-thiazoline was cumbersome due to the free phenol. Next, the heterocyclic TMS-imines, **5l** and *in situ* prepared **5m** and **5n**, gave the corresponding products in 83%, 76%, and 32% yields, respectively. Surprisingly, styryl-substituted TMS-imine **5o** did not react under the optimized reaction conditions. No potential 1,4-addition products were observed, likely due to the highly electrophilic conditions around the imine and α-carbon. With *tert*-butyl-substituted **7p**, we could demonstrate that aliphatic TMS-imines are compatible with the modified Asinger methodology. However, the product was unstable under silica gel chromatography and was purified by distillation, resulting in a 31% yield. Finally, to assess the feasibility of α-hydroxyketones, imine **5a** was reacted with α-hydroxyacetone to produce **7q** in a 13% yield.

Next, we studied the scope of in situ-formed α-sulfanyl derivatives (**6b–f**, Scheme 5) as inputs for our modified Asinger protocol. Initially, we utilized simple aliphatic and aromatic substituents at the R^1^ position of **9b**–**f** (Scheme 3). After *in situ* formation of the α-sulfanyl-carbonyls (**6c**–**d**) and trapping with **5a,** the 3-thiazolines **7s** and **7t** were obtained in yields of 65% and 59%, respectively.

**Scheme 5:**
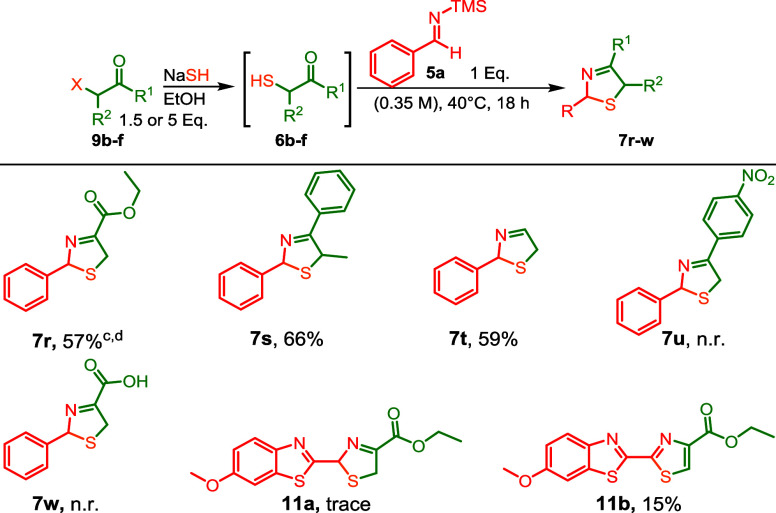
α-Haloketone Scope of the 3-Thiazoline Three-Component Reaction^a^ ^b^ ^c^ ^d^

a Standard conditions: NaSH (3.35 mmol), α-acylhalide (3.35 mmol) in EtOH, 0 °C, 1 h. TMS-imine (0.67 mmol), 40 °C, nitrogen, 18 h.
b Isolated yields.
c Thiol component isolated prior to reaction.
d 5 equiv thiol used.

As we envisioned to use our modified Asinger protocol in the synthesis of luciferin (and analogs, *vide infra*), we first tried the reaction using ethyl-3-bromo-2-oxopropanoate (**9b**) with NaSH under optimized conditions. The yield was significantly lower (15%) compared with our earlier successes. We observed that the initially formed 3-thiazoline core partially oxidized to the final thiazole product. However, by increasing the stoichiometry of **9b** and NaSH to 5 equiv each, the yield of **7r** improved to 68%, as determined by ^1^H NMR. Upon isolation by column chromatography, the product was also oxidized to the corresponding thiazole. We then isolated ethyl 3-mercapto-2-oxopropanoate **6b** before reaction with **5a** as oxidation is likely facilitated by traces of elemental sulfur in commercial NaSH.^2^ This two-step process allowed the isolation of 3-thiazoline **5p** in a moderate yield of 57%. Unfortunately, following a similar procedure to access **7u** and **7w**; no desired product was observed.

Having demonstrated the compatibility of TMS-imines in the Asinger reaction, we turned to more complex examples, aiming for a concise and efficient luciferin (and analogues) synthesis. First, we performed the modified Asinger reaction of *in situ* generated 2-benzothiazole TMS-imine (**5m**) with **6b**, which resulted in the trace formation of 3-thiazoline **11a**. Even under optimized conditions, we could isolate only around 15% of thiazole **11b**, with undesired oxidation occurring during purification (Scheme 5), with only trace amounts of the desired 3-thiazoline **11a**. Performing the reaction using Schlenk techniques resulted in slightly better formation of only 3-thiazoline **11a**. Switching to HFIP at 60 °C did not improve the yield of either the desired 3-thiazoline or the oxidized thiazole. However, at more elevated temperatures (Scheme 6) in HFIP, surprisingly, the regioisomeric 2-thiazoline **12** was formed in 40% yield after isolation. The structure of 2-thiazoline product **12** was confirmed by independent synthesis (compound **13**, Section S4.7), adapting literature procedures of White et al.^22^ and Suzuki et al.^23^

**Scheme 6:**
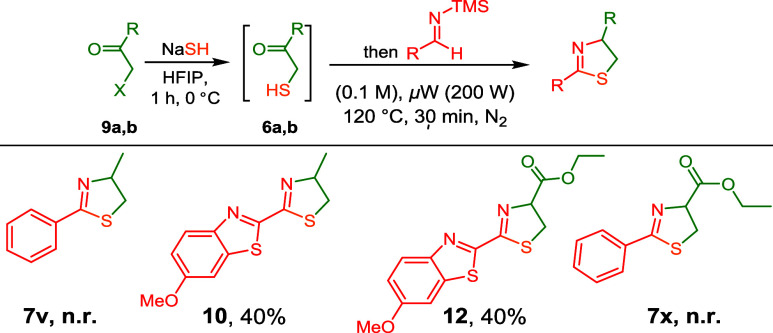
Bond Isomerization of **3** to 2-Thiazoline and Application in Luciferin Synthesis through the Asinger-MCR

Intrigued by this, we investigated whether similar regioisomeric 2-thiazolines (instead of 3-thiazolines) are formed when the benzothiazole imine **5m** reacts with **6a** under these conditions. Indeed, 2-thiazoline **10** was exclusively formed in a yield of 40% after isolation (Scheme 6). In contrast, the reaction of TMS-imine **5a** with either α-sulfanyl-ketones **6a** or **6b** gave exclusively the corresponding 3-thiazolines (**7a** and **7r**, respectively). The benzothiazole substituent in the TMS-imine input is crucial for the formation of 2-thiazolines, as other heterocyclic substituents (**7n**) do not show this occurrence. We hypothesized that 2-thiazoline formation results from the isomerization of the initially formed 3-thiazoline. To demonstrate this, we dissolved 3-thiazolines **7a,** **d**, **h**, **l**, and **m** in HFIP and subjected them to microwave irradiation at 120 °C for 0.5 h. However, no isomerizations were observed for **7a**, **d**, **h**, and **l**, even with further temperature increases and extended reaction times. We did observe trace amounts of 2-thiazoline **10** forming during the isomerization of **7m**. When 1 equiv of trimethylsilyl chloride was added to **7m** in HFIP and subjected to microwave irradiation at 120 °C for 0.5 h, a 55% conversion to 2-thiazoline **10** was detected (Section S5). This suggests that the SiMe_3_ substituent plays a role in the isomerization mechanism.

Although unexpected, with 2-thiazoline **12** in hand, it already contained the core framework of firefly luciferin. To access the natural product, we only needed to cleave the benzothiazole methoxy substituent and ethyl ester. We first deprotected the methoxy substituent by treating 2-thiazoline **12** with BBr_3_ in DCM. After an aqueous workup, the crude product was directly used without purification in the hydrolysis of the ethyl ester. After screening a wide range of hydrolysis conditions, we found that pig liver esterase (PLE)^24^ allowed the selective formation of luciferin **8** in a 30% isolated yield over two steps, with an ee% of 68% for the l-luciferin enantiomer.

Based on the above experiments and observations, we propose a mechanism for the formation of 3-thiazoline **7m** and 2-thiazoline **10** as depicted in Scheme 7 (a detailed mechanism is provided in Section S9). Initially, and in analogy with the conventional Asinger mechanism, the process starts with the addition of the thiol to the imine (steps **5m** to **I**). Subsequent cyclization forms intermediate **II**. This intermediate can undergo two pathways: (a) 3-thiazoline **7m** is generated through the elimination of Me_3_SiOH. We observed a slight conversion of 3-thiazoline **7m** to 2-thiazoline **10** under the reaction conditions, demonstrating that this can be a viable pathway. However, pathway (b), which involves direct isomerization via migration of the SiMe_3_ group to the benzothiazole nitrogen, seems more likely, as 2-thiazoline **10** is generated significantly faster when starting from TMS-imine **5m** and thiol **6a**. Moreover, since the addition of Me_3_SiCl to 3-thiazoline **7m** facilitates the isomerization to 2-thiazoline **10** (details in the Supporting Information), we speculate that it promotes the formation of intermediate **III** and isomerization from **IV**.

**Scheme 7:**
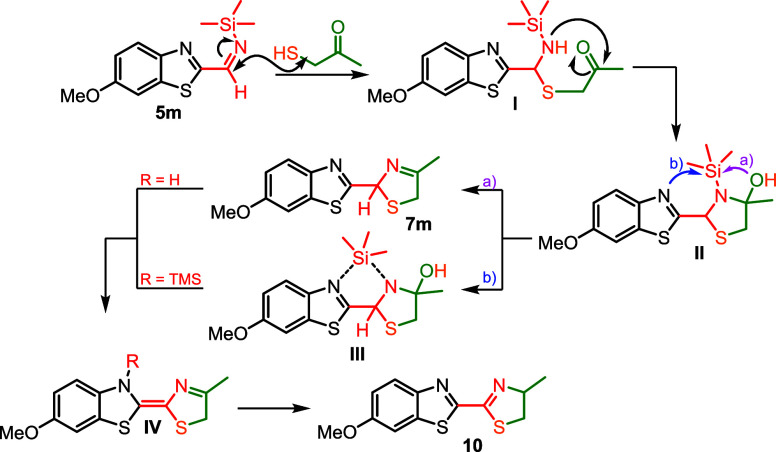
Proposed Reaction Mechanism toward 3-Thiazoline (**7m**) and 2-Thiazoline (**10**) Products by the Asinger Reaction

In conclusion, we developed a variation on the classical A-4CR using preformed TMS-imines in combination with α-halo ketones or aldehydes and NaSH. The one-pot, two-step procedure involves the trapping of initially formed α-sulfanyl ketones or aldehydes. A wide range of imines were tolerated, which are typically challenging in the classical/resynthesized A-4CR. Furthermore, we expanded the utility of this methodology by accessing both 2-thiazolines and 3-thiazolines from imine **7m** by simply switching conditions: 60 °C in EtOH and 120 °C in HFIP. We could exploit this efficient approach toward **12** in the synthesis of (±)-firefly luciferin **8**, achieving a 30% yield over two steps.

## Supplementary Material



## Data Availability

The data underlying this study are available in the published article and its Supporting Information.

## Notes

Detailed experimental procedures, spectroscopic data for all new compounds, and copies of ^1^H and ^13^C­{^1^H} NMR spectra (PDF)
