# Supplementary material for: Multicomponent Synthesis of 3‑Thiazolines Using a Modified Asinger-Type Reaction
Source: J Org Chem. 2025 Jun 3;90(23):7971–5. doi: 10.1021/acs.joc.5c00563 (PMC12172030; doi:10.1021/acs.joc.5c00563)
Supplement: Supplementary file 1 [file jo5c00563_si_001.pdf]

Supporting Information

Multicomponent Synthesis of 3-Thiazolines using a Modified Asinger-type Reaction

Vincent R.A.M. Reinartz[1], Jordy M. Saya[1], Darya Hadavi[2], Jules A.W. Harings[1] and Romano V.A. Orrù[1]*

[1]*Aachen Maastricht Institute for Biobased Materials (AMIBM), Maastricht University, Urmonderbaan 22 6167 RD Geleen, The Netherlands*

[2]*Maastricht MultiModal Molecular Imaging Institute (M4I), Maastricht University, Universiteitssingel 50 6229 ER Maastricht, The Netherlands*

**: Corresponding author: r.orru@maastrichtuniversity.nl*

Table of Contents

1. General Information 1

2. Optimization studies of 4-methyl-2-phenyl-2,5-dihydrothiazole (7a) 2

3. Optimization Studies of ethyl 2-phenyl-2,5-dihydrothiazole-4-carboxylate (7r) 5

4. Experimental Section 8

   4.1. Synthesis of TMS-Imines 8

   4.2. General procedure: Microwave assisted Asinger MCR 14

   4.3. Synthesis of α-ketonethiols 21

   4.4. General Procedure: Conventional heating 21

   4.5 Benzothiazole Precursor Procedure for Luciferin (8) synthesis 23

   4.6. General Procedure 2-thiazoline and Luciferin Synthesis 24

   4.7. L-luciferin 2-thiazoline reference material synthesis 26

   5. Isomerisation Experiments 28

   6. Spectral Data TMS-Imines 36

7. Spectral Data Thiazolines 51

8. Spectral data α-keto-thiol, 2-thiazolines, precursors and Luciferin Analogs 73

9. Isomerization mechanism of 3-thiazoline (7m) to 2-thiazoline (10) 83

10. References 83

1. General Information

Unless stated otherwise, all solvents and commercially available reagents used were purchased from Merck (Sigma Aldrich), Fluorochem/Doug Discovery, Fisher Scientific, TCI Chemicals and Bio Solve. Unless stated otherwise, all solvents and commercially available reagents were used as purchased. Anhydrous THF, DMF and Ethanol were obtained by either Inert Solvent purification system, commercially sourced or dried by molecular sieves (pore size 4Å). Thin layer chromatography (TLC) was performed on TLC plates from Merck (SiO_2, Kieselgel 60 F254 neutral, aluminium backed with fluorescent indicator). The compounds on the TLC plates were visualised by UV detection (254 or 366 nm) and stained by either $KMnO_4$ or p-anisaldehyde. All reactions that were heated (unless stated otherwise) were performed in biotage 0.5 to 20 mL microwave vials, crimped with a PFTE metal septum and heated with a closely fitted aluminium heating block set to the desired temperature. Flash Column Chromatography (FCC) was performed using Silica gel (VWR, particle size: 40-63 µm) as stationary phase. Elution was performed by mixtures of n-heptane:Ethyl acetate or Dichloromethane:Methanol as mobile phases. In addition, Automatic Flash column chromatography was performed by a Büchi Reveleris X2 Flash column chromatography system, using pre-packed Büchi Eco-flex columns (25 g of SiO_2, 50 µm irregular particles). NMR spectra were recorded on a Brüker Avance 300 MHz (75 MHz for ^{13}C) using the residual solvent[1] or TMS as internal reference: $CDCl_3$ (^1H: δ 7.26 ppm, ^{13}C: δ 77.16 ppm), MeOD d_4(1H: δ 3.31 ppm, ^{13}C: δ 49.00 ppm) DMSO d_6 (^1H: δ 7.26 ppm, ^{13}C: δ 77.16 ppm) and TMS (^1H: δ 0.00 ppm). Chemical shifts (δ) are given in ppm and coupling constants (J) are quoted in hertz (Hz). Resonances are described as s (singlet), d (doublet), t (triplet), q (quartet), quint (quintet), sex (sextet), sep (septet), br (broad singlet) and m (multiplet) or combinations thereof. Perkin Elmer Spotlight 400 FT(N)IR microscope and PerkinElmer Spotlight 400, equipped with a PIKE GladiATR, dual mode MCT (mercury cadmium telluride) detector with an array or a temperature-stabilized DTGS (deuterated triglycine sulphate) as a standard configuration. Measurements were performed in the range of 4000-650 cm^{-1}, with a resolution of 0.5 or 4 cm-1. The number of scans per sample for static measurements were 32. Electrospray ionization (ESI) high-resolution mass spectrometry was carried out on Bruker SolariX XR-FT-ICR-MS (ion type mass analyser) Samples were ionized by electrospray ionization (ESI) in positive ion mode. Enantiomeric excess (ee%) were determined using a Optical Activity AA-10 Polarimeter with a 589.44 nm wave filter and a cell with a length of 50 mm. The microwave Reactions were performed in a Biotage Initiator+ Microwave Synthesizer in either 5- or 20-mL microwave vails crimped with a septum. Preparative Liquid chromatography was performed on a Shimadzu Nexera HPLC Prep system with a Shim-pack Scepter 20 mm, C-18 functionalized column with a particle size of 5 µm and a flow rate of 20 mL/min; using an eluent system of acetonitrile and milli-q water modified with 0.1 % v/v formic acid.

2. Optimization studies of 4-methyl-2-phenyl-2,5-dihydrothiazole (7a)

2.1 initial scouting runs

Table S1: initial scouting runs[a]

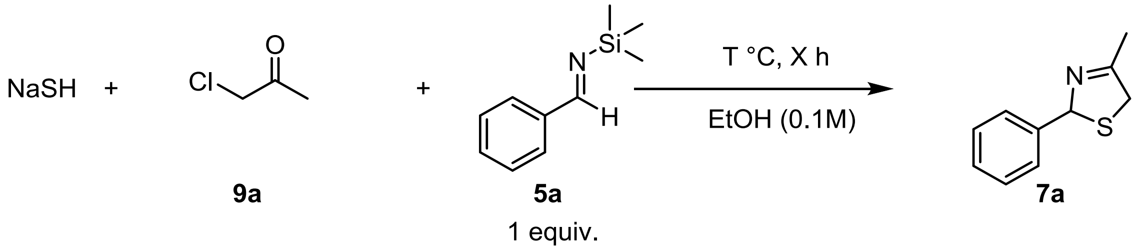

Entry	Temperature (°C)	Equiv.	Reaction Time (h)	Atmosphere (Air/N_2)	Yield (%)[b]
1	40	1.2	18	Air	56
2	40	2	18	Air	51
3	Rt.	1.2	18	Air	52
4	50	1.2	18	Air	14
5	50	1.2	3	Air	63
6	60	1.2	18	Air	58
7	60	1.2	3	Air	62
**8**	**60**	**1.2**	**3**	**N_2**	**78**

[a]Reaction conditions: 0.34 mmol **5a**.

[b]Yield determined by internal standard using 2.5-dimethylfuran

2.2 Solvent effect

Table S2: Solvent effect[a]

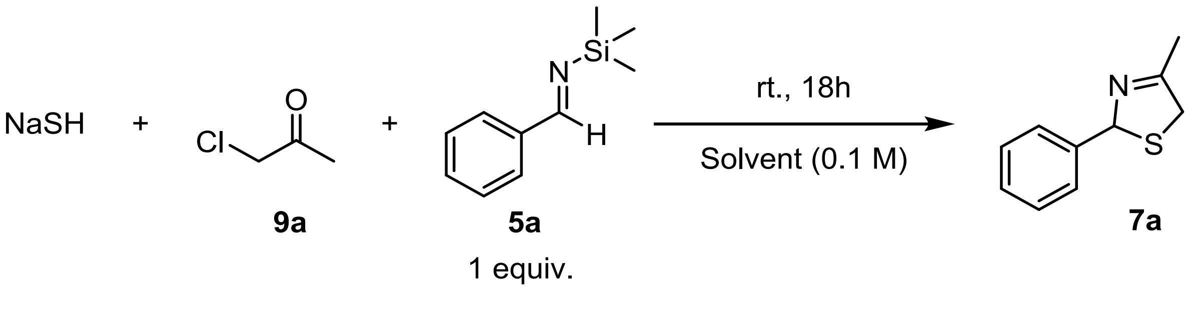

Entry	Solvent	Yield (%)[b]
1	HFIP	58
2	MeCN	35
3	DCM	37
4	Water	43

[a] Reaction Conditions: 0.34 mmol **5a**, 0.41 mmol **NaSH,** 0.45 mmol **9a**

[b] Yield determined by internal standard using 2.5-dimethylfuran.

2.3 Concentration

Table S3: Concentration effect[a]

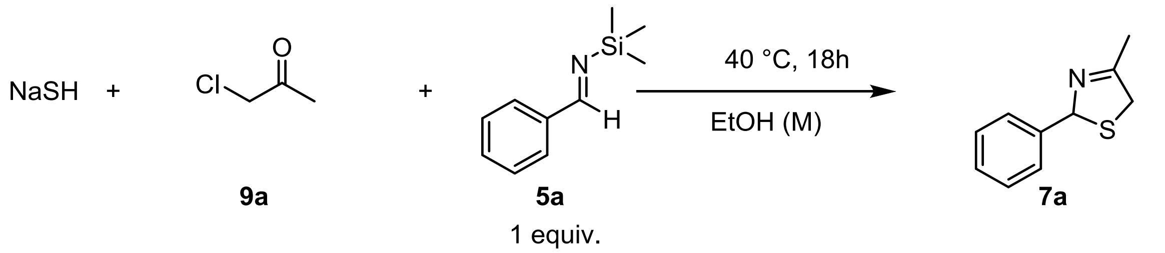

Entry	Concentration (M)	Yield (%)[b]
**1**	**0.097**	**56**
2	0.17	46
3	0.34	50
4	0.5	35
5	1	42
6	2	28

[a] Reaction Conditions: 0.34 mmol **5a**, 0.44 mmol **NaSH,** 0.44 mmol **9a**

[b] Yield determined by internal standard using 2.5-dimethylfuran.

2.4 Reaction Time

Table S4: Time effect[a]

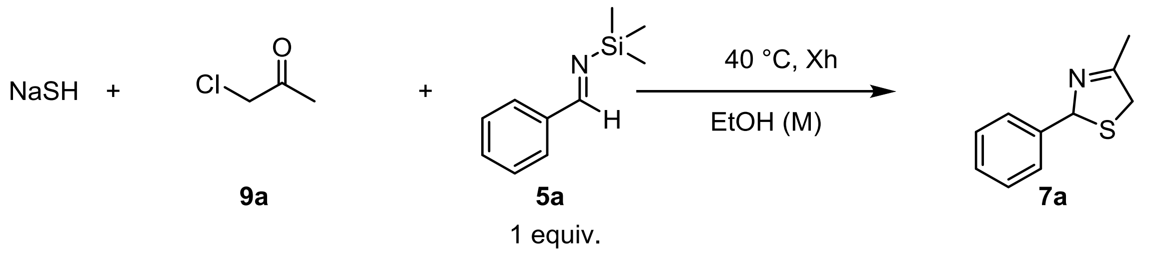

Entry	Reaction Time (h)	Yield (%)[b]
1	1	69
2	2	68
**3**	**3**	**77**
4	5	77
5	6	73
6	7	75
7	8	65
8	18	58

[a] Reaction Conditions: 0.34 mmol **5a**, 0.44 mmol **NaSH,** 0.44 mmol **9a**

[b] Yield determined by internal standard using 2.5-dimethylfuran.

2.5 Microwave vs conventional heating

Table S5: Heating effect

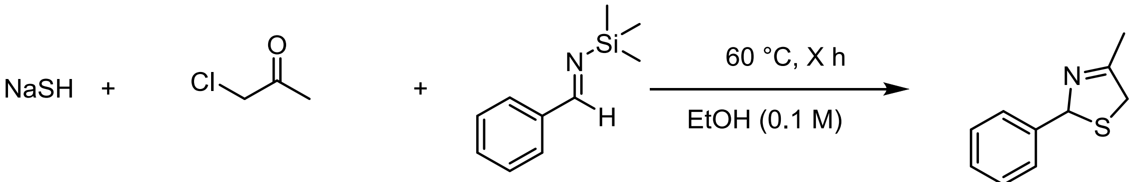

9a **5a** **7a**

1 equiv

Entry	Microwave Or Conventional	Reaction time (h)	Atmosphere (Air/N_2)	Yield (%)[b]
1	Conventional	3	N_2	78
2	Conventional	3	Air	62
3	Microwave	0.5	Air	63
4	Microwave	0.5	Air	62[c, d]
5	Microwave	0.5	Air	62[c]
6	Microwave	0.5	Air	79[e]
7	Microwave	1	Air	58
8	Microwave	0.5	Air	64[f]
9	Microwave	0.5	N_2	76
**10**	**Microwave**	**0.5**	**N_2**	**92[c]**

[a]Reaction condition: 0.34 mmol **5a**, 0.51 mmol **NaSH** and 0.51 mmol **9a**

[b]Yield determined by internal standard using 2.5-dimethylfuran.

[c] Solvent dried utilizing 4 Å Molecular sieves under N_2

[d]2 equiv of **NaSH** and 1.5 equiv. of **9a** were utilized.

[e] 1 mmol scale towards 5**a** was utilized

[f] Mercaptoacetone (**6a**) was added as a premade compound

3. Optimization Studies of ethyl 2-phenyl-2,5-dihydrothiazole-4-carboxylate (7r)

3.1. Solvent Effect

Table S6: Solvent effect[a]

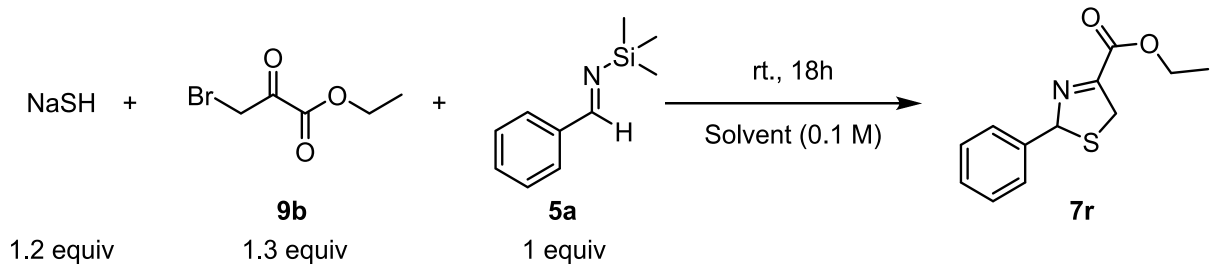

Entry	Solvent	Yield (%)[b]
**1**	**EtOH**	**37**
**2**	**HFIP**	**38**
3	MEOH	20
4	MeCN	5
5	EtOAc	6
6	Toluene	15
7	DCM	13
8	Chloroform	16
9	Water	12
10	IPA	16
11	DMF	10
12	Diethylether	18
13	TBME	14
14	THF	17
15	DMSO	8
16	Trifluoroethanol	6

[a] Reaction Conditions: 0.34 mmol **5a**, 0.41 mmol **NaSH**, 0.45 mmol of **9b**

[b] Yield determined by internal standard using 2.5-dimethylfuran.

3.2. Concentration

Table S7: Concentration effect[a]

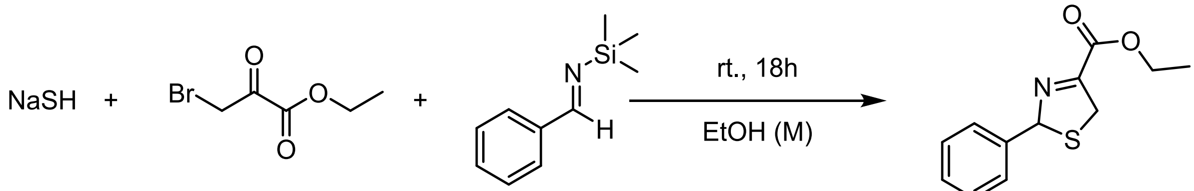

NaSH	9b	5a	7r
1.2 equiv	1.3 equiv	1 equiv	

Entry	Concentration (M)	Yield (%)[b]
1	0.1	26
2	0.17	25
**3**	**0.34**	**33**
4	0.5	15
5	0.75	21
6	1	22
7	2	14

[a] Reaction Conditions: 0.34 mmol **5a**, 0.41 mmol **NaSH,** 0.45 mmol of **9b**

[b] Yield determined by internal standard using 2.5-dimethylfuran.

3.3. Equivalents

Table S8: Stoichiometry effect[a]

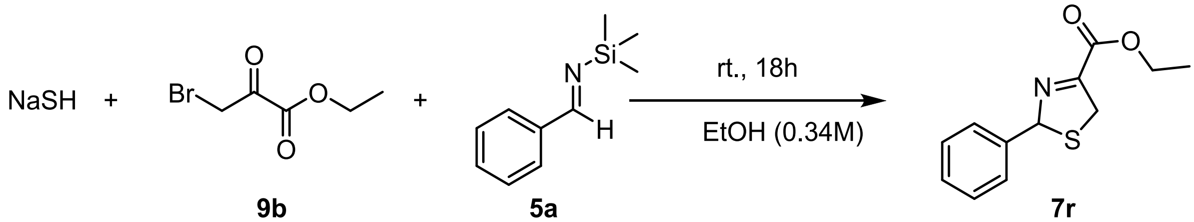

NaSH	9b	5a	7r
X equiv	X equiv	1 equiv	

Entry	Equiv. towards 5a	Yield (%)[b]
1	1.2	4
2	1.5	10
3	2	30
4	3	36
5	4	36
**6**	**5**	**68**

[a] Reaction Conditions: 0.34 mmol **5a**

[b] Yield determined by internal standard using 2.5-dimethylfuran.

3.4. Temperature, Reaction time and atmosphere

Table S9: Temperature, Reaction time and Atmosphere[a]

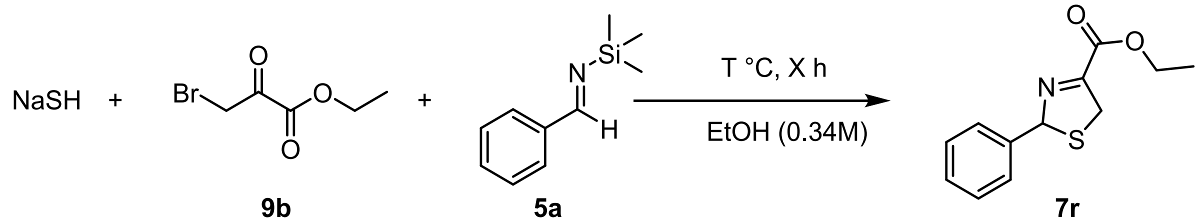

9b 5a 7r

5 equiv 5 equiv 1 equiv

Entry	Temperature (°C)	Reaction Time (h)	Atmosphere (Air/N_2)	Yield (%)[b]
1	Room (20-25)	18	Air	58
2	40	18	Air	79
3	40	18	Air	68[d]
4	60	18	Air	69
5	60	3	N_2	67[c]
6	60	96	N_2	36
**7**	**40**	**18**	**N_2**	**85[e]**

[a] Reaction Conditions: 0.34 mmol **5a**, 0.41 mmol **NaSH,** 0.45 mmol of **9b**.

[b] Yield determined by internal standard using 2.5-dimethylfuran.

c Reaction concentration was 0.13 M

[d]10 equiv. of sodiumsulfate was added as a drying agent inside the reaction.

[e]isolated **6b** was used.

4. Experimental Section

4.1. Synthesis of TMS-Imines

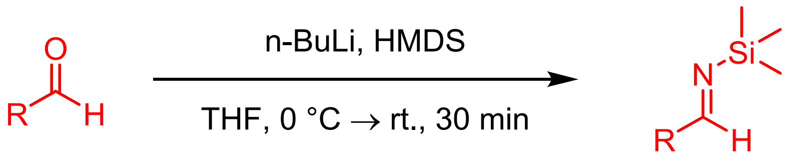

A 25 mL flask was purged from air and placed under nitrogen atmosphere. To the flask, bis(trimethylsilyl)amine (8.08 mmol, 1.1 Eq.) was added and cooled with an ice bath, followed by the slow addition of a 2.5 M n-butyllithium solution in hexanes (7.35 mmol 1 Eq.) over a period of 5 min. The ice bath was removed and the mixture was left to stir at room temperature for an additional 20 min. After this time, the solvent was removed in vacuo until a white slurry had formed. At this time the flask was again placed under nitrogen atmosphere and cooled to 0 °C using an ice-bath. The flask was charged with an aldehyde (7.35 mmol, 1 Eq.) dissolved in 3 mL of dry THF and allowed to stir for 5 min. after this time, the ice-bath was removed and left further to react on for an additional 15 to 20 min. This was followed by the removal of the residual solvent in vacuo by rotary evaporation to obtain the crude imine mixture. Further purification was performed by bulb-to-bulb distillation under reduced pressure to obtain the corresponding N-Trimethylsilylimine.

*1-phenyl-N-(trimethylsilyl)methanimine (**5a**)*

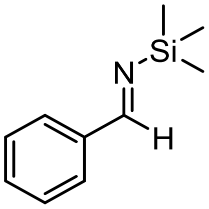

The imine was prepared using: benzaldehyde (1.000 g, 9.423 mmol), bis(trimethylsilyl)amine (1.673 g, 10.73 mmol) and 1.6 M n-butyllithium solution in hexanes (0.604 g, 5.890 mL, 9.423 mmol). N-Trimethylsilyl imine purification: Bulb to Bulb distillation, 95-100 °C at 1 mbar, affording a light-yellow liquid (651.2 mg, 39 %). The characterization data matched the data reported in literature.[2,3]

^{1}H NMR: (300 MHz, Chloroform-d) δ 7.87 – 7.74 (m, 2H), 7.43 (dd, J = 5.1, 1.9 Hz, 3H), 0.26 (s, 9H). **^{13}C{^{1}H} NMR:** (75 MHz, Chloroform-d) δ 159.4, 129.7, 127.2, 126.5, 125.9, 91.3, 76.1, 75.7, 75.3, 0.0. **FTIR** (V_{max}/cm^{-1}): 2957 (-CH=C-, m), 1641 (-CH=NR, w), 1250 (-CH=NR, m), 837 (-Si-CH_3, sbr), 752 (-Si-C, m).

*1-(2,6-dimethylphenyl)-N-(trimethylsilyl)methanimine (**5b**)*

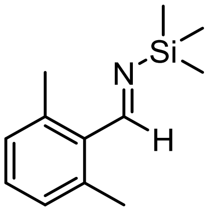

The imine was prepared using 2,6-dimethylbenzaldehyde (1.000 g, 7.453 mmol), bis(trimethylsilyl)amine (1.323 g, 8.198 mmol) and 2.5 M n-butyllithium solution in hexanes (0.477 g, 2.981 mL, 7.453 mmol). N-Trimethylsilyl imine purification: Bulb to Bulb distillation, 120-130 °C at 1 mbar, affording a-yellow liquid (633.8 mg, 47 %).

^{1}H NMR: (300 MHz, Chloroform-d) δ 9.34 (s, 1H), 7.13 (dd, J = 8.3, 6.7 Hz, 1H), 7.02 (d, J = 7.5 Hz, 2H), 2.40 (s, 6H), 0.26 (s, 9H). **^{13}C {^{1}H} NMR:** (75 MHz, Chloroform-d) δ 171.4, 137.9, 130.1, 129.8, 129.6, 21.2, 0.0. **FTIR** (V_{max}/cm^{-1}): 2958 (-CH=C-, w), 1635 (-CH=NR, m), 1251 (-CH=NR, s), 838 (-Si-CH_3, m doublet), 768 (-Si-C, s). **HRMS (ESI)** m/z $[M+H]^+$calculated for $C_{12}H_{20}NSi$ 206.1360; Found 206.1375.

*1-(4-(tert-butyl)phenyl)-N-(trimethylsilyl)methanimine (**5c**)*

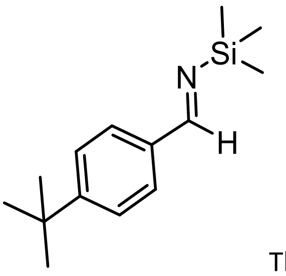

The imine was prepared using 4-(tert-butyl)benzaldehyde (1.000 g, 6.164 mmol), bis(trimethylsilyl)amine (1.094 g, 6.780 mmol) and 2.5 M n-butyllithium solution in hexanes (0.395 g, 2.466 mL, 6.352 mmol). N-Trimethylsilyl imine purification: Bulb to Bulb distillation, 160 °C at 1 mbar, affording a light-yellow oil (1.191 g, 83 %).

^{1}H NMR: (300 MHz, Chloroform-d) δ 8.95 (s, 1H), 7.73 (d, J = 8.4 Hz, 1H), 7.45 (d, J = 8.4 Hz, 1H), 1.34 (s, 9H), 0.25 (s, 9H). **^{13}C {^{1}H} NMR**: (75 MHz, Chloroform-d) δ 169.5, 158.9, 129.4, 127.2, 126.6, 126.4, 32.3, 2.5, 0.0. **FTIR** (V_{max}/cm^{-1}): 2956 (-CH=C-, m), 1655 (-CH=NR, m), 1247 (-CH=NR, m), 827 (-Si-CH_3, s doublet), 745 (-Si-C, m). **HRMS (ESI)** m/z $[M+H]^+$ calculated for $C_{14}H_{24}NSi$ 234.1673; Found 234.1682.

*1-(4-methoxyphenyl)-N-(trimethylsilyl)methanimine (**5d**)*

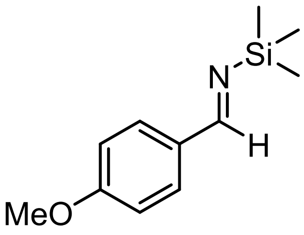

The imine was prepared using 4-methoxybenzaldehyde (1.000 g, 7.345 mmol), bis(trimethylsilyl)amine (1.304 g, 8.079 mmol) and 2.5 M n-butyllithium solution in hexanes (0.471 g, 2.938 mL, 7.345 mmol). N-Trimethylsilyl imine purification: Bulb to Bulb distillation,150 °C at 1 mbar, affording a yellow oil (735.6 mg, 48 %).

^{1}H NMR: (300 MHz, Chloroform-d) δ 8.90 (s, 1H), 7.75 (d, J = 8.6 Hz, 2H), 6.94 (d, J = 8.5 Hz, 2H), 3.85 (s, 4H), 0.24 (s, 9H). **^{13}C {^{1}H} NMR:** (75 MHz, Chloroform-d) δ 168.9, 131.2, 114.9, 78.5, 78.1, 77.6, 56.5, 0.0. **FTIR** (V_{max}/cm^{-1}): 2965 (-CH=C-), 1602 (-CH=NR), 1244 (-OMe), 1160 (-CH=NR), 829 (-Si-CH_3), 739 (-Si-C). **HRMS (ESI)** m/z $[M+H]^+$ calculated for $C_{11}H_{18}NOSi$ 208.1152; Found 208.1153.

*1-(3-methoxyphenyl)-N-(trimethylsilyl)methanimine (**5e**)*

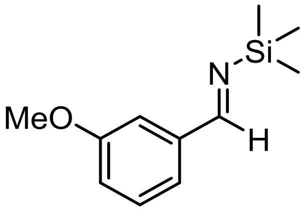

The imine was prepared using 3-methoxybenzaldehyde (1.000 g, 7.345 mmol), bis(trimethylsilyl)amine (1.304 g, 8.079 mmol) and 2.5 M n-butyllithium solution in hexanes (0.471 g, 2.938 mL, 7.345 mmol). N-Trimethylsilyl imine purification: Bulb to Bulb distillation, 130 °C at 1 mbar, affording a-yellow liquid (778.4 mg, 51 %).

^{1}H NMR: (300 MHz, Chloroform-d) δ 8.87 (s, 1H), 7.32 (td, J = 3.1, 2.5, 1.5 Hz, 1H), 7.28 (t, J = 1.2 Hz, 1H), 7.26 (d, J = 1.0 Hz, 1H), 6.96 – 6.90 (m, 1H), 3.79 (s, 3H), 0.19 (s, 9H). **^{13}C {^{1}H} NMR:** (75 MHz, Chloroform-d) δ 168.33, 158.2, 129.5, 121.8, 118.8, 118.1, 117.4, 112.5, 112.3, 112.1, 78.7, 77.4, 77.0, 76.6, 55.4, 55.2, 1.3, 0.5, -1.1. **FTIR** (V_{max}/cm^{-1}): 2962 (-CH=C-, w), 1603 (-CH=NR, m), 1239 (-CH=NR, s), 1167 (-OMe, m), 818 (-Si-CH_3, m doublet), 756 (-Si-C, w). **HRMS (ESI)** m/z [M+H]$^+$ calculated for $C_{11}H_{18}NOSi$ 208.1152; Found 208.1150.

*1-(2-methoxyphenyl)-N-(trimethylsilyl)methanimine (**5f**)*

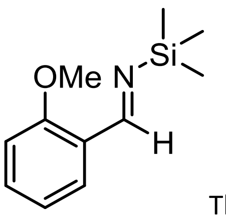

The imine was prepared using 2-methoxybenzaldehyde (1.000 g, 7.345 mmol), bis(trimethylsilyl)amine (1.304 g, 8.079 mmol) and 2.5 M n-butyllithium solution in hexanes (0.471 g, 2.938 mL, 7.345 mmol). N-Trimethylsilyl imine purification: Bulb to Bulb distillation, 120-130 °C at 1 mbar, affording a-yellow liquid (344 mg, 23 %).

^{1}H NMR: (300 MHz, Chloroform-*d*) δ 9.40 (s, 1H), 7.96 (dd, *J* = 7.6, 1.9 Hz, 1H), 7.40 (ddd, *J* = 8.8, 7.3, 1.9 Hz, 1H), 6.98 (dt, *J* = 19.0, 7.5 Hz, 2H), 3.89 (s, 3H), 0.25 (s, 9H). **^{13}C {^{1}H} NMR:** (75 MHz, Chloroform-d) δ 159.4, 129.7, 127.2, 126.5, 125.9, 91.3, 76.1, 75.7, 75.3,0.0. **FTIR** (V_{max}/cm^{-1}): 2956 (-CH=C-, w), 1598 (-CH=NR, m), 1239 (-CH=NR, s), 1159 (-OMe, m), 832 (-Si-CH_3, m doublet), 749 (-Si-C, w). **HRMS (ESI)** m/z [M+H]$^+$ calculated for $C_{11}H_{18}NOSi$ 208.1152; Found: 208.1168.

*1-(4-(trifluoromethyl)phenyl)-N-(trimethylsilyl)methanimine (**5g**)*

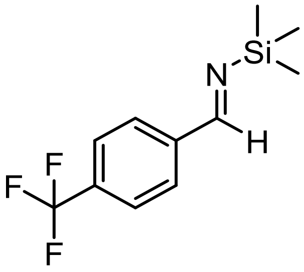

The imine was prepared using 4-(trifluoromethyl)benzaldehyde (1.000 g, 5.743 mmol), bis(trimethylsilyl)amine (1.020 g, 6.317 mmol) and 2.5 M n-butyllithium solution in hexanes (0.368 g, 2.297 mL, 7.345 mmol). N-Trimethylsilyl imine purification: Bulb to Bulb distillation,110-130 °C at 1 mbar, affording a yellow liquid (526.7 mg, 37 %).

^{1}H NMR (300 MHz, Chloroform-d) δ 9.00 (s, 1H), 7.93 – 7.87 (m, 2H), 7.69 (dd, J = 8.3, 2.2 Hz, 2H), 0.27 (s, 9H). **^{13}C {^{1}H} NMR**: (75 MHz, Chloroform-d) δ 158.8, 127.6. 126.4, 126.3, 125.1, 124.4, 90.0, -2.1. **FTIR** (V_{max}/cm^{-1}): 2962 (-CH=C-, w), 1633 (-CH=NR, mbr), 1319 (-CH=NR, s), 1065 (-CF_3, s), 1017 (-Si-CH_3, s), 837 (-Si-C, s). **HRMS (ESI)** m/z $[M+H]^+$ calculated for $C_{11}H_{15}F_3NSi$ 246.0920; Found 246.0938.

*4-(((trimethylsilyl)imino)methyl)benzonitrile (**5h**)*

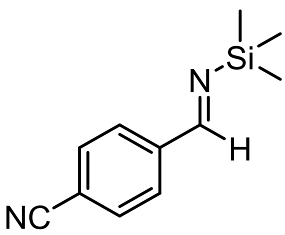

The imine was prepared using 4-formylbenzonitrile (1.000 g, 7.626 mmol), bis(trimethylsilyl)amine (1.354 g, 8.389 mmol) and 2.5 M n-butyllithium solution in hexanes (0.489 g, 3.050 mL, 6.164 mmol). N-Trimethylsilyl imine purification: Bulb to Bulb distillation, 140-160 °C at 1 mbar, affording a yellow/green solid (196.7 mg, 13 %).

^{1}H NMR: (300 MHz, Chloroform-d) δ 8.98 (s, 1H), 7.89 (dt, J = 8.3, 1.7 Hz, 2H), 7.83 – 7.58 (m, 2H), 0.26 (s, 9H). **^{13}C {^{1}H} NMR**: (75 MHz, Chloroform-d) δ 167.3. 158.3, 133.8, 130.2, 128.6, 78.9, 1.8. **FTIR** (V_{max}/cm^{-1}): 2957 (-CH=C-, w), 2229 (-CN, m) 1646 (-CH=NR, mbr), 1248 (-CH=NR, m), 829 (-Si-CH_3, sbr), 749 (-Si-C, sbr). **HRMS (ESI)** m/z $[M+H]^+$ calculated for $C_{11}H_{15}N_2Si$ 203.0999; Found 203.1083.*

*1-(4-bromophenyl)-N-(trimethylsilyl)methanimine (**5i**)*

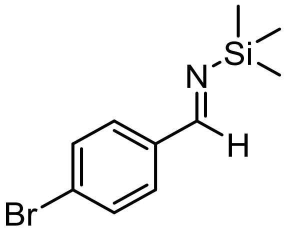

The imine was prepared using 4-bromobenzaldehyde (1.000 g, 5.405 mmol), bis(trimethylsilyl)amine (0.960 g, 5.945 mmol) and 2.5 M n-butyllithium solution in hexanes (0.346 g, 2.162 mL, 5.405 mmol). N-Trimethylsilyl imine purification: Bulb to Bulb distillation, 140-150 °C at 1 mbar, affording a-yellow oil (448.9 mg, 32 %).

^{1}H NMR: (300 MHz, Chloroform-d) δ 8.90 (s, 1H), 7.66 (dd, J = 8.5, 1.9 Hz 2H), 7.57 (dd, J = 8.5, 1.9 Hz 2H), 0.25 (s, 9H). **^{13}C {^{1}H} NMR:** (75 MHz, Chloroform-d) δ 168.2, 158.4, 133.1, 133.0, 132.7, 131.0, 130.1, 129.5, 79.2, 0.0. **FTIR** (V_{max}/cm^{-1}): 2957 (-CH=C-, m), 1652 (-CH=NR, w), 1248 (-CH=NR, m), 1010 (-Br, m), 833 (-Si-CH_3, sbr), 739 (-Si-C, m). **HRMS (ESI)** m/z $[M+H]^+$ calculated for $C_{10}H_{15}BrNSi$ 256,0152; Found 256.0158.

* HRMS of 5h has an error of 41.4 ppm, this inaccuracy is due to the instability of 5h in acidic and aqueous conditions required for the HRMS measurement.

*1-(2-bromophenyl)-N-(trimethylsilyl)methanimine (**5j**)*

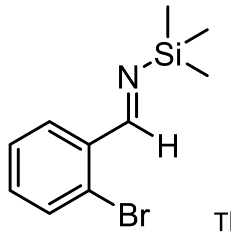

The imine was prepared using 2-bromobenzaldehyde (1.000 g, 5.405 mmol), bis(trimethylsilyl)amine (0.960 g, 5.945 mmol) and 2.5 M n-butyllithium solution in hexanes (0.346 g, 2.162 mL, 5.405 mmol). N-Trimethylsilylimine purification: Bulb to Bulb distillation,130-140 °C at 1 mbar, affording a yellow liquid (696 mg, 50 %).

^{1}H NMR: (300 MHz, Chloroform-d) δ 9.26 (s, 1H), 8.00 (dd, J = 7.6, 2.0 Hz, 1H), 7.57 (dd, J = 7.8, 1.2 Hz, 1H), 7.40 – 7.23 (m, 2H), 0.27 (s, 9H). **^{13}C {^{1}H} NMR:** (75 MHz, Chloroform-d) δ 158.0, 133.3, 133.2, 132.5, 132.0, 129.3, 128.9, 128.8, 128.2, 127.9, 127.7, 77.7, 77.6, 77.2, 76.7, 1.5, 0.8, -1.0. **FTIR** (V_{max}/cm^{-1}): 2957 (-CH=C-, w), 1636 (-CH=NR, m), 1248 (-CH=NR, m), 1026 (C-Br, w), 829 (-Si-CH_3, sbr), 749 (-Si-C, sbr). **HRMS (ESI)** m/z $[M+H]^+$ calculated for $C_{10}H_{15}BrNSi$ 256,0152; Found 256.0150.

*1-(furan-2-yl)-N-(trimethylsilyl)methanimine (**5l**)*

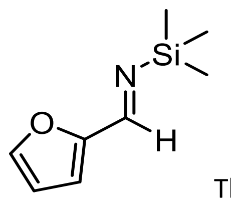

The imine was prepared using furan-2-carbaldehyde (1.000 g, 10.41 mmol), bis(trimethylsilyl)amine (1.848 g, 11.45 mmol) and 2.5 M n-butyllithium solution in hexanes (0.346 g, 4.163 mL, 10.41 mmol). N-Trimethylsilyl imine purification: Bulb to Bulb distillation,105-110 °C at 1 mbar, affording a dark yellow liquid (644.4 mg, 37 %).

^{1}H NMR: (300 MHz, Chloroform-d) δ 8.67 (s, 1H), 7.53 (s, 1H), 6.83 (d, J = 2.5 Hz, 1H), 6.49 (s, 1H), 0.24 (s, 9H). **^{13}C {^{1}H} NMR:** (75 MHz, Chloroform-d) δ 149.2, 144.0, 129.7, 114.4, 110.5, 109.1, 106.5, 82.8, 76.1, 75.7, 75.3, 0.0. **FTIR** (V_{max}/cm^{-1}): 2958 (-CH=C-, w), 1645 (-CH=NR, m), 1249 (-CH=NR, s), 1151 (-C-O-C-, w), 829 (-Si-CH_3, sbr), 749 (-Si-C, sbr). **HRMS (ESI)** m/z $[M+H]^+$ calculated for $C_8H_{14}NOSi$ 168.0839; Found 168.0847.

*3-phenyl-N-(trimethylsilyl)prop-2-en-1-imine (**5o**)*

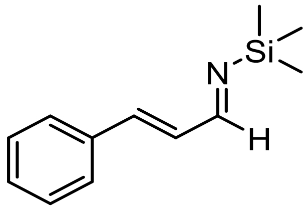

The imine was prepared using cinnamaldehyde (1.000 g, 7.567 mmol), bis(trimethylsilyl)amine (1.343 g, 8.324 mmol) and 2.5 M n-butyllithium solution in hexanes (0.485 g, 3.027 mL, 7.567 mmol). N-Trimethylsilyl mine purification: Bulb to Bulb distillation, 140-155 °C at 4 mbar, affording a light-orange oil (114.7 mg, 8 %).

^{1}H NMR: (300 MHz, Chloroform-*d*) δ 1H NMR (300 MHz, Chloroform-d) δ 8.70 (d, J = 8.3 Hz, 1H), 7.55 – 7.45 (m, 3H), 7.42 – 7.34 (m, 4H), 7.10 (d, J = 15.9 Hz, 1H), 6.83 (dd, J = 15.9, 8.3 Hz, 1H), 0.23 (s, 8H).**^{13}C {^{1}H} NMR:** (75 MHz, Chloroform-d) δ 171.7, 146.1, 133.2, 130.7, 130.1, 128.7, 78.7, 78.2, 77.8, 0.0. **FTIR** (V_{max}/cm^{-1}): 2956 (-CH=C-, w), 2896 (-CH=CH-,w), 1632 (-CH=NR, m), 1250 (-CH=NR, s), 837 (-Si-CH_3, m doublet), 747 (-Si-C, w). **HRMS (ESI):** m/z $[M+H]^+$ calculated for $C_{12}H_{18}NSi$ 204.1203; Found 204.1203.

*2,2-dimethyl-N-(trimethylsilyl)propan-1-imine (**5p**)*

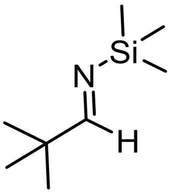

The imine was prepared using pivalaldehyde (1.000 g, 11.61 mmol), bis(trimethylsilyl)amine (2.061 g, 12.77 mmol) and 2.5 M n-butyllithium solution in hexanes (0.744 g, 4.644 mL, 11.61 mmol). N-Trimethylsilyl mine purification: Bulb to Bulb distillation, 55-80 °C at <100 mbar, affording a colourless liquid (725 mg, 40 %)

^{1}H NMR: (300 MHz, Chloroform-d) δ 7.43 (d, J = 0.9 Hz, 1H), 1.06 (s, 9H), 0.05 (s, 9H). **^{13}C {^{1}H} NMR:** (75 MHz, Chloroform-d) δ 168.1, 97.2, 83.2, 25.3, 24.6, 23.5, 0.0, -0.53. **FTIR** (V_{max}/cm^{-1}): 2954 (-CH, m), 1668 (-CH=NR, m), 1326 (-Me, m), 1248 (-CH=NR, s), 833 (-Si-CH_3, s), 744 (-Si-C, m). **HRMS (ESI)** m/z $[M+H]^+$ calculated for $C_8H_{20}NSi$ 158.1356; Found 158.1360.

4.2. General procedure: Microwave assisted Asinger MCR

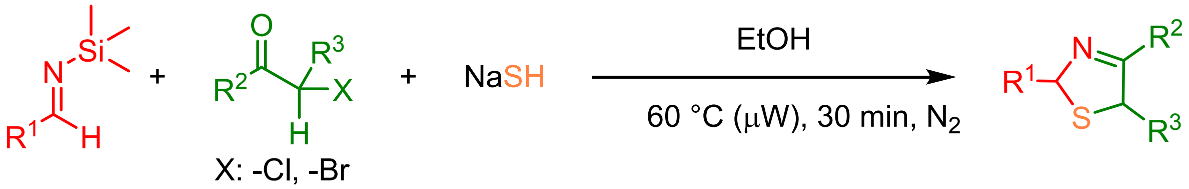

To a 10 mL microwave vial, sodium hydrogensulfide (56.9 mg, 1.01 mmol 1.5 Eq.) was added and placed under a nitrogen atmosphere. To this, Ethanol (5.0 mL, 0.1 M) was added and the contents was left to stir for 5-10 min. The vial was cooled to 0°C using an ice-bath, once cooled the α-haloketone (1.02 mmol 1.5 Eq.) was added and left to stir for 1 hour at 0°C. After this time N-Trimethylsilyl mine (0.67 mmol, 1.0 Eq.) was added. The vial cap was replaced, capped and placed in the microwave reactor for 30 min, at 60 °C using 200 Watts of power with a maximum pressure of 6 bar. After the reaction was allowed to cool to room temperature, the content was transferred to a separatory funnel using Ethyl acetate (20 mL) and water (20 mL). The layers were separated and the aqueous layer was once more extracted using ethyl acetate (20 mL). The organic layers were combined and dried by brine (10 mL) and Na_2SO_4, followed by the removal of solvent under reduced pressure. Isolation of the products was performed by flash column chromatography using mixtures of 10-80% n-heptane in ethyl acetate to afford the corresponding products.

*4-methyl-2-phenyl-2,5-dihydrothiazole (**7a**)*

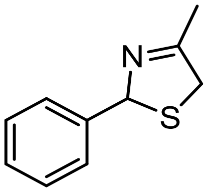

The 3-thiazoline was prepared using commercially available N-Trimethylsilylbenzaldimine **5a** (120 mg, 0.677 mmol), 1-chloropropan-2-one (94.0 mg, 80.9 µL, 1.0 mmol) and sodium hydrogensulfide (56.9 mg, 1.0 mmol). 3-Thiazoline purification: Flash column chromatography, 6:1 to 3:1 heptane:ethyl acetate afforded a pale-yellow solid (89.6 mg, 75 %).

R_f: 0.3 in 1:1 n-heptane:ethylacetate. **^{1}H NMR:** (300 MHz, Chloroform-d) δ 7.38 – 7.23 (m, 5H), 6.62 (ddq, J = 5.2, 3.7, 1.9 Hz, 1H), 4.06 (dq, J = 15.9, 12.7, 3.1 Hz, 2H), 2.25 (d, J = 1.8 Hz, 3H). **^{13}C {^{1}H} NMR:** (75 MHz, CDCl3) δ 128.6, 128.0, 126.8, 84.6, 77.5, 77.0, 76.6, 47.3, 19.6. **HRMS (ESI)** m/z $[M+H]^+$ calculated for $C_{10}H_{12}NS$ 178.0685; Found 178.0686.

*2-(2,6-dimethylphenyl)-4-methyl-2,5-dihydrothiazole (**7b**)*

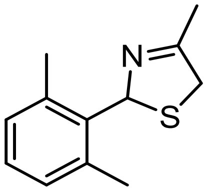

The 3-thiazoline was prepared using **5b** (139 mg, 0.677 mmol), 1-chloropropan-2-one (94.0 mg, 80.9 µL, 1.0 mmol) and sodium hydrogensulfide (56.9 mg, 1.0 mmol). 3-Thiazoline purification: Flash column chromatography, 6:1 to 3:1 heptane:ethyl acetate afforded a light-orange oil (118.6 mg, 85 %).

R_f: 0.5 in 2:1 n-heptane:ethylacetate. **^{1}H NMR:** (300 MHz, Chloroform-d) δ 7.10 – 6.96 (m, 4H), 4.21 – 3.97 (m, 2H), 2.34 (s, 6H), 2.21 (d, J = 2.2 Hz, 3H).**^{13}C {^{1}H} NMR:** (75 MHz, CDCl3) δ 169.4, 137.0, 129.5, 127.9, 81.0, 77.5, 77.0, 76.6, 47.8, 20.4, 19.6. **HRMS (ESI)** m/z $[M+H]^+$calculated for $C_{12}H_{16}NS$ 206,0998; Found 206.0999.

*2-(4-(tert-butyl)phenyl)-4-methyl-2,5-dihydrothiazole (**7c**)*

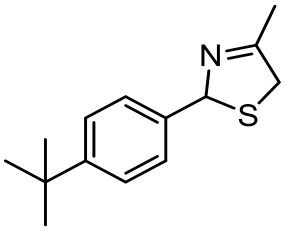

The 3-thiazoline was prepared using **5c** (142 mg, 0.608 mmol), 1-chloropropan-2-one (94.0 mg, 80.9 µL, 1.02 mmol) and sodium hydrogensulfide (56.9 mg, 1.02 mmol). 3-Thiazoline purification: Flash column chromatography, 6:1 to 2:1 heptane:ethyl acetate afforded a light-yellow oil (141.5 mg, 83 %).

R_f: 0.4 in 2:1 n-heptane:ethylacetate. **^{1}H NMR:** (300 MHz, Chloroform-d) δ 7.39 – 7.31 (m, 2H), 7.27 – 7.22 (m, 3H), 6.60 (ddt, J = 5.1, 3.6, 1.8 Hz, 1H), 4.07 (ddd, J = 17.6, 12.7, 4.7 Hz, 2H), 2.23 (d, J = 1.8 Hz, 3H), 1.30 (s, 9H).**^{13}C {^{1}H} NMR:** (75 MHz, CDCl3) δ 142.8, 110.4, 106.9, 77.5, 77.0, 76.6, 46.7, 19.6. **HRMS (ESI)** m/z $[M+H]^+$ calculated for $C_{14}H_{19}NS$ 234.1311; Found 234.1317.

*2-(4-methoxyphenyl)-4-methyl-2,5-dihydrothiazole (**7d**)*

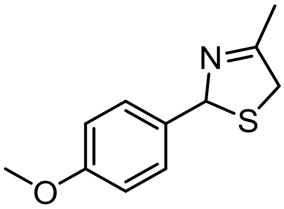

The 3-thiazoline was prepared using **5d** (142.6 mg, 0.687 mmol), 1-chloropropan-2-one (94.0 mg, 80.9 µL, 1.02 mmol) and sodium hydrogensulfide (56.9 mg, 1.02 mmol). 3-Thiazoline purification: Flash column chromatography, 6:1 to 3:1 heptane: ethyl acetate afforded a light-yellow oil (117.6 mg, 84 %).

R_f: 0.4 in 1:1 heptane: ethyl acetate. **^{1}H NMR:** (300 MHz, Chloroform-*d*) δ 7.24 (t, 2H), 6.87 (t, 2H), 6.58 (ddq, J = 5.1, 3.6, 1.9 Hz, 1H), 4.06 (ddd, 2H), 3.79 (s, 3H), 2.23 (d, J = 1.8 Hz, 3H). **^{13}C {^{1}H} NMR:** (75 MHz, CDCl3) δ 169.9, 132.0, 128.6, 128.0, 114.3, 114.0, 84.3, 77.5, 77.0, 76.6, 55.6, 55.3, 47.2, 41.7, 19.6. **HRMS (ESI)** m/z $[M+H]^+$ calculated for $C_{11}H_{14}NOS$ 208.0791; Found 208.0791.

*2-(3-methoxyphenyl)-4-methyl-2,5-dihydrothiazole (**7e**)*

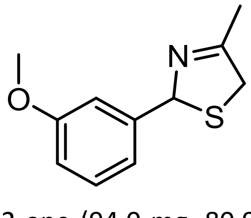

The 3-thiazoline was prepared using **5e** (142 mg, 0.685 mmol), 1-chloropropan-2-one (94.0 mg, 80.9 µL, 1.0 mmol) and sodium hydrogensulfide (56.9 mg, 1.0 mmol). 3-Thiazoline purification: Flash column chromatography, 6:1 to 3:1 heptane:ethyl acetate afforded a yellow oil (97.5 mg, 68 %).

R$_f$: 0.2 in 2:1 n-heptane:ethylacetate. **^{1}H NMR:** (300 MHz, Chloroform-d) δ 7.26 (t, J = 7.8 Hz, 1H), 6.97 – 6.78 (m, 3H), 6.60 (tt, J = 3.3, 1.8 Hz, 1H), 4.05 (dq, J = 15.0, 12.8, 3.2 Hz, 2H), 3.81 (s, 3H), 2.25 (d, J = 1.8 Hz, 3H). **^{13}C {^{1}H} NMR:** (75 MHz, chloroform-d) δ 129., 121.1, 119.1, 118.3, 113.5, 112.7, 112.4, 47.2, 44.3, 27.7, 19.6. **HRMS (ESI)** m/z [M+H]$^+$ calculated for $C_{11}H_{14}NOS$ 208.0791; Found 208.0792.

*2-(3-methoxyphenyl)-4-methyl-2,5-dihydrothiazole (**7f**)*

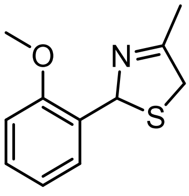

The 3-thiazoline was prepared using **5f** (142 mg, 0.685 mmol), 1-chloropropan-2-one (94.0 mg, 80.9 µL, 1.0 mmol) and sodium hydrogensulfide (56.9 mg, 1.0 mmol). 3-Thiazoline purification: Automatic Flash column chromatography, 6:1 to 3:1 heptane:ethyl acetate afforded a light-yellow oil (75.6 mg, 54 %).

R$_f$: 0.2 in 3:1 n-heptane:ethylacetate. **^{1}H NMR:** (300 MHz, Chloroform-d) δ 7.34 – 7.13 (m, 2H), 6.98 – 6.82 (m, 2H), 3.97 (t, J = 4.2 Hz, 2H), 3.88 (s, 3H), 2.27 (d, J = 1.8 Hz, 3H).**^{13}C {^{1}H} NMR:** (75 MHz, CDCl3) δ 170.8, 128.7, 126.3, 120.7, 110.4, 78.7, 77.5, 77.0, 76.6, 55.5, 46.2, 46.2, 19.8. **HRMS (ESI)** m/z [M+H]$^+$ calculated for $C_{11}H_{14}NOS$ 208.0791; Found 208.0791.

*4-methyl-2-(4-(trifluoromethyl)phenyl)-2,5-dihydrothiazole (**7g**)*

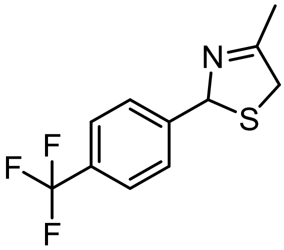

The 3-thiazoline was prepared using **5g** (142 mg, 0.579 mmol), 1-chloropropan-2-one (94.0 mg, 80.9 µL, 1.02 mmol) and sodium hydrogensulfide (56.9 mg, 1.02 mmol). 3-Thiazoline purification: Flash column chromatography, 6:1 to 2:1 heptane:ethyl acetate afforded a light-yellow oil (97.1 mg, 68 %).

R$_f$: 0.3 in 2:1 n-heptane:ethylacetate. **^{1}H NMR:** (300 MHz, Chloroform-*d*) δ 7.60 (d, J = 8.1 Hz, 2H), 7.42 (d, J = 8.1 Hz, 2H), 6.64 (s, 1H), 4.08 (ddd, J = 9.5, 12.8, 3.5 Hz, 2H), 2.27 (d, J = 1.8 Hz, 3H). **^{13}C {^{1}H} NMR:** (75 MHz, CDCl3) δ 127.1, 125.6, 77.4, 77.0, 76.6, 47.5, 19.6. **HRMS (ESI):** m/z [M+H]$^+$ calculated for $C_{11}H_{11}F_3NS$ 246.0559; Found 246.0558.

*4-(4-methyl-2,5-dihydrothiazol-2-yl)benzonitrile (**7h**)*

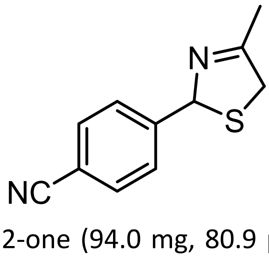

The 3-thiazoline was prepared using **5h** (130 mg, 0.643 mmol), 1-chloropropan-2-one (94.0 mg, 80.9 µL, 1.0 mmol) and sodium hydrogensulfide (56.9 mg, 1.0 mmol). 3-Thiazoline purification: Flash column chromatography, 6:1 to 3:1 heptane:ethyl acetate afforded a pale-yellow solid (106.4 mg, 83 %).

R_f: 0.5 in 1:1 n-heptane:ethylacetate. **^{1}H NMR:** (300 MHz, Chloroform-d) δ 7.64 (dt, J = 8.3, 1.9 Hz, 2H), 7.40 (dt, J = 6.3, 1.9 Hz, 2H), 6.62 (tt, J = 3.4, 1.8 Hz, 1H), 4.08 (dq, J = 12.4, 6.7, 3.4 Hz, 2H), 2.27 (d, J = 1.8 Hz, 3H). **^{13}C {^{1}H} NMR:** (75 MHz, CDCl3) δ 132.5, 127.5, 83.7, 77.4, 77.0, 76.6, 47.5, 19.6. **HRMS (ESI)** m/z $[M+H]^+$ calculated for $C_{11}H_{11}N_2S$ 203.0638; Found 203.0638.

*2-(4-bromophenyl)-4-methyl-2,5-dihydrothiazole (**7i**)*

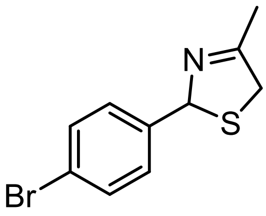

The 3-thiazoline was prepared using **5i** (171 mg, 0.667 mmol), 1-chloropropan-2-one (94.0 mg, 80.9 µL, 1.0 mmol) and sodium hydrogensulfide (56.9 mg, 1.0 mmol). 3-Thiazoline purification: Flash column chromatography, 6:1 to 5:1 heptane:ethyl acetate afforded a light-yellow oil (101.5 mg, 57 %).

R_f: 0.3 in 2:1 n-heptane:ethylacetate. **^{1}H NMR:** (300 MHz, Chloroform-d) δ 1H NMR (300 MHz, Chloroform-d) δ 7.51 – 7.40 (m, 2H), 7.23 – 7.13 (m, 2H), 6.55 (tt, J = 3.3, 1.8 Hz, 1H), 4.04 (qd, J = 16.0, 4.1 Hz, 2H), 2.23 (d, J = 1.8 Hz, 3H). **^{13}C {^{1}H} NMR:** (75 MHz, CDCl3) δ 170.9, 141.1, 132.1, 131.7, 131.4, 129.0, 128.5, 121.8, 83.9, 77.5, 77.1, 76.7, 47.4, 19.6. **HRMS (ESI):** m/z $[M+H]^+$ calculated for $C_{10}H_{11}BrNS$ 255.9790; Found 255.9790.

*2-(2-bromophenyl)-4-methyl-2,5-dihydrothiazole (**7j**)*

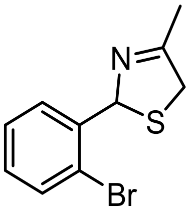

The 3-thiazoline was prepared using **5j** (171 mg, 0.667 mmol), 1-chloropropan-2-one (94.0 mg, 80.9 µL, 1.02 mmol) and sodium hydrogensulfide (56.9 mg, 1.02 mmol). 3-Thiazoline purification: Flash column chromatography, 6:1 to 2:1 heptane:ethyl acetate afforded a light-yellow oil (141.5 mg, 85 %).

R_f: 0.6 in 1:1 n-heptane:ethylacetate. **^{1}H NMR:** (300 MHz, Chloroform-d) δ 7.56 (dd, J = 8.0, 1.3 Hz, 1H), 7.29 (td, J = 7.4, 1.2 Hz, 1H), 7.23 – 7.08 (m, 2H), 6.93 (tq, J = 3.7, 1.9 Hz, 1H), 4.01 (dd, J = 4.0, 1.5 Hz, 2H), 2.29 (d, J = 1.8 Hz, 3H).**^{13}C {^{1}H} NMR:** (75 MHz, CDCl3) δ 132.7, 129.1, 127.6, 83.6, 77.5, 77.0, 76.6, 46.6, 19.8. **HRMS (ESI):** m/z calculated for $C_{10}H_{11}BrNS$ $[M+H]^+$ 255.9790, found: 255.9791.

*4-(4-methyl-2,5-dihydrothiazol-2-yl)phenyl acetate(**7k**)*

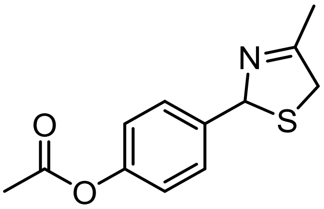

The 3-thiazoline was prepared in three consecutive steps. bis(trimethylsilyl)amine (480.0 mg, 2.979 mmol, 2.2 Eq) and 2.5 M n-butyllithium solution in hexanes (0.174 g, 1.083 mL, 2.708 mmol, 2 Eq) were mixed for 20 minutes. The hexanes were removed in vacuo until a white slurry/powder remained, this was dissolved in 1 mL of dry THF and cooled with an ice bath. 4-hydroxybenzaldehyde (165.4 mg, 1.354 mmol, 1 Eq) was dissolved in 2 mL of dry THF and added dropwise, forming a clear orange solution and was further reacted on room temperature for 20-30 minutes. After this time, Acetic acid (81.3 mg, 77.4 µL, 1.354 mmol, 1 Eq) was added and the solvent and excess acid was removed under vacuo. The remaining orange solid was redissolved in 2.5 mL of ethanol under nitrogen atmosphere. At the same time in a separate flask, sodium hydrogensulfide (113.9 mg, 2.031 mmol, 1.5 Eq) was placed under nitrogen, dissolved in 2.5 mL of ethanol, cooled with an ice bath and 1-chloropropan-2-one (161.7 µL, 187.9 mg, 2.031 mmol, 1.5 Eq) was added at once. This mixture was allowed to stir for 1 hour. After this time both mixtures were added together under nitrogen and left to react inside the microwave reactor according to the general procedure above. After reaction, the content was transferred to a new flask using ethyl acetate, the solvent was removed under vacuo and redissolved in dry THF. To this solution, acetic anhydride (414.7 mg, 383.3 µL, 4.062 mmol, 3 Eq) and dimethylaminopyridine (165.4 mg, 1.354 mmol, 1 Eq) were added and left to react overnight under nitrogen atmosphere at room temperature. The following morning, the reaction mixture was transferred to a separatory funnel, washed with water, extracted with ethyl acetate and dried with brine and Na_2SO_4. The solvent was removed under vacuo to afford the crude 3-thiazoline as a brown oil. 3-Thiazoline purification: Flash column chromatography, 3:1 to 2:1 heptane:ethyl acetate afforded an orange oil (98.1 mg, 31 % over three steps).

R_f: 0.2 in 2:1 n-heptane:ethyl acetate. **^{1}H NMR:** (300 MHz, Chloroform-d) δ 7.38 – 7.22 (m, 2H), 7.11 – 7.00 (m, 2H), 6.60 (ddq, J = 5.1, 3.6, 1.9 Hz, 1H), 4.05 (qd, J = 16.0, 4.1 Hz, 2H), 2.28 (s, 3H), 2.23 (d, J = 1.8 Hz, 3H).**^{13}C {^{1}H} NMR:** (75 MHz, CDCl3) δ 170.5, 169.4, 150.3, 139.5, 122.1, 121.9, 121.9, 121.8, 121.6, 84.0, 77.5, 77.1, 76.6, 47.3, 21.2, 19.6. **HRMS (ESI)** m/z $[M+H]^+$ calculated for $C_{12}H_{14}NO_2S$ 236.0740; Found 236.0742.

*2-(furan-2-yl)-4-methyl-2,5-dihydrothiazole (**7l**)*

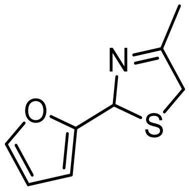

The 3-thiazoline was prepared using **5l** (113 mg, 0.677 mmol), 1-chloropropan-2-one (94.0 mg, 80.9 µL, 1.02 mmol) and sodium hydrogensulfide (56.9 mg, 1.02 mmol). 3-Thiazoline purification: Flash column chromatography, 6:1 to 1:1 heptane:ethyl acetate afforded a dark yellow oil (93.2 mg, 83 %).

R_f: 0.5 in 1:1 n-heptane:ethylacetate. **^{1}H NMR:** (300 MHz, Chloroform-d) δ 7.38 (dd, J = 1.8, 0.9 Hz, 1H), 6.63 (ddq, J = 4.6, 3.3, 1.8 Hz, 1H), 6.36 – 6.25 (m, 2H), 4.02 (ddd, J = 23.6, 13.3, 2.7 Hz, 2H), 2.22 (d, J

= 1.7 Hz, 3H).**^{13}C {^{1}H} NMR:** (75 MHz, CDCl3) δ 142.8, 110.4, 106.9, 77.5, 77.4, 77.0, 76.6, 46.7, 19.6. **HRMS (ESI)** m/z $[M+H]^+$ calculated for $C_8H_{11}NOS$ 168.0478; Found 168.0478.

*6-methoxy-2-(4-methyl-2,5-dihydrothiazol-2-yl)benzo[d]thiazole (**7m**)*

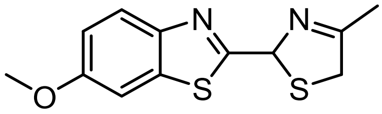

The 3-thiazoline was prepared in two consecutive steps. bis(trimethylsilyl)amine (55 mg, 0.34 mmol, 1.1 Eq) and 2.5 M n-butyllithium solution in hexanes (20 mg, 0.120 mL, 0.31 mmol, 1 Eq) were mixed for 20 minutes. The hexanes were removed in vacuo until a white slurry/powder remained, this was dissolved in 1 mL of dry THF and cooled with an ice bath. 6-methoxybenzo[d]thiazole-2-carbaldehyde **16** (60.2 mg, 0.312 mmol, 1 Eq) was dissolved in 2 mL of dry THF and added dropwise, forming a clear red solution and was further reacted on room temperature for 20-30 minutes. After this time, Acetic acid (81.3 mg, 77.4 µL, 1.354 mmol, 1 Eq) was added and the solvent was removed under vacuo. The remaining red solid was redissolved in 2.5 mL of ethanol under nitrogen atmosphere. At the same time in a separate flask, sodium hydrogensulfide (113.9 mg, 2.031 mmol,1.5 Eq) was placed under nitrogen, dissolved in 2.5 mL of ethanol, cooled with an ice bath and 1-chloropropan-2-one (161.7 µL, 187.9 mg, 2.031 mmol, 1.5 Eq) was added at once. This mixture was allowed to stir for 1 hour. After this time both mixtures were added together under nitrogen and left to react inside the microwave reactor according to the general procedure above. the reaction mixture was transferred to a separatory funnel, washed with water, extracted with ethyl acetate and dried with brine and Na_2SO_4. The solvent was removed under vacuo to afford the crude 3-thiazoline as a brown/orange oil. 3-Thiazoline purification: Automatic flash column chromatography, 9:1 to 7:3 heptane:ethyl acetate using a 25-gram SiO_2 flashpure eco-flex cartridge, afforded an orange solid (62 mg, 76 % over 2 steps).

R_f: 0.3 in 1:1 n-heptane:ethylacetate. **^{1}H NMR:** (300 MHz, Chloroform-d) δ 7.90 (d, J = 9.0 Hz, 1H), 7.30 (d, J = 2.6 Hz, 1H), 7.07 (dd, J = 9.0, 2.5 Hz, 1H), 6.96 (ddq, J = 4.9, 3.4, 1.8 Hz, 1H), 4.11 (dq, J = 13.1, 7.0, 3.0 Hz, 2H), 3.87 (s, 3H), 2.30 (d, J = 1.7 Hz, 3H), 1.61 (s, 1H). **^{13}C {^{1}H} NMR:** (75 MHz, CDCl3) δ 123.9, 115.5, 104.2, 82.0, 77.5, 77.0, 76.6, 55.8, 47.3, 19.7. **HRMS (ESI)** m/z $[M+H]^+$ calculated for $C_{12}H_{13}N_2OS_2$ 265.0464; Found 265.0467.

*2-(6-methoxypyridin-2-yl)-4-methyl-2,5-dihydrothiazole (**7o**)*

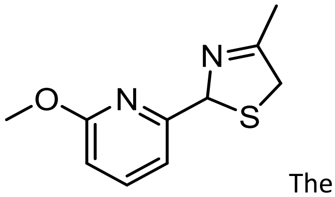

The 3-thiazoline was prepared in two consecutive steps. bis(trimethylsilyl)amine (118 mg, 0.734 mmol, 1.1 Eq) and 2.5 M n-butyllithium solution in hexanes (42.7 mg, 0.267 mL, 0.667 mmol, 1 Eq) were mixed for 10 minutes. This Mixture was dissolved in 1 mL of dry THF and cooled with an ice bath. 6-Methoxypyridine-2-carboxaldehyde (91.5 mg, 0.667 mmol, 1 Eq) was dissolved in 1 mL of dry THF and added, forming a clear light-yellow solution and was further reacted on room temperature for 20 minutes. After this time, Acetic acid (20 mg, 19.1 µL,0.334 mmol, 0.5 Eq) was added and the solvent was removed under vacuo. The remaining solid was redissolved in 2.5 mL of ethanol under nitrogen atmosphere. At the same time in a separate flask, sodium hydrogensulfide (56.1 mg, 1.0 mmol,1.5 Eq) was placed under nitrogen, dissolved in 2.5 mL of ethanol, cooled with an ice bath and 1-chloropropan-2-one (92.6 mg,79.9 µL 1.0 mmol, 1.5 Eq) was added at

once. This mixture was allowed to stir for 1 hour. After this time both mixtures were added together under nitrogen and left to react inside the microwave reactor according to the general procedure above. The reaction mixture was transferred to a flask and the solvent was removed under vacuo to afford the crude 3-thiazoline as an orange waxy solid. 3-Thiazoline purification: flash column chromatography, 3:1 to 1:3 n-heptane:ethyl acetate, afforded a dark-orange oil (43.8 mg, 32 % over 2 steps).

R_f:0.3 in 1:1 ethylacetate:n-heptane. **^{1}H NMR:** (400 MHz, Chloroform-*d*) δ 7.48 (ddd, *J* = 8.2, 7.2, 0.8 Hz, 1H), 6.79 (dd, *J* = 7.4, 0.9 Hz, 1H), 6.60 – 6.55 (m, 2H), 3.96 (ddt, *J* = 15.8, 13.4, 2.8, 2.5 Hz, 2H), 3.86 (s, 3H), 2.22 (s, 3H). **^{13}C NMR:** (101 MHz, chloroform-d) δ 202.9, 171.9, 163.8, 158.6, 139.4, 139.2, 112.8, 109.9, 85.4, 53.4, 46.6, 41.8, 28.5, 19.8. **HRMS (ESI)** m/z $[M+H]^+$ calculated for $C_{10}H_{13}N_2OS$ 209.0743; Found 209.0741.

*2-(tert-butyl)-4-methyl-2,5-dihydrothiazole (**7p**)*

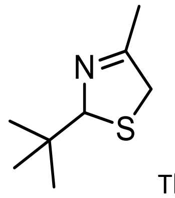

The 3-thiazoline was prepared using **5p** (998.5 mg, 6.347 mmol), 1-chloropropan-2-one (882.1 mg, 759.1 µL, 9.534 mmol) and sodium hydrogensulfide (534.5 mg, 9.534 mmol). 3-Thiazoline purification: Bulb to bulb distillation, 110-120 °C at 1 mbar afforded a colourless liquid (310.2 mg, 31%)

^{1}H NMR: (300 MHz, Chloroform-d) δ 5.47 (ddt, J = 5.1, 3.5, 1.7 Hz, 1H), 3.77 (t, J = 3.4 Hz, 2H), 2.13 (d, J = 1.8 Hz, 3H), 0.98 (s, 9H). **^{13}C {^{1}H} NMR:** (75 MHz, CDCl3) δ 95.1, 77.4, 77.0, 76.6, 45.6, 26.4, 19.5. **HRMS (ESI)** m/z $[M+H]^+$ calculated for $C_8H_{16}NS$ 158.0998; Found 158.0998.

*4-methyl-2-phenyl-2,5-dihydrooxazole (**7q**)*

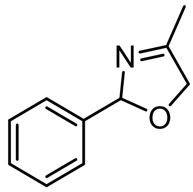

The 3-thiazoline was prepared using commercially available N-Trimethylsilylbenzaldimine **5a** (120 mg, 0.677 mmol) and α-hydroxyacetone (90 % purity, 83.6 mg, 1.02 mmol). 3-oxazole purification: Flash column chromatography, 3:1 to 1:1 heptane:ethyl acetate afforded a yellow oil (14.2 mg, 13 %).

R_f: 0.3 in 3:1 heptane:ethylacetate. **^{1}H NMR:** (400 MHz, Chloroform-*d*) δ 7.45 – 7.25 (m, 5H), 6.58 (ddp, *J* = 5.4, 3.7, 1.8 Hz, 1H), 4.74 – 4.53 (m, 2H), 2.12 (s, 3H). **^{13}C NMR:** (101 MHz, chloroform-d) δ 169.6, 139.9, 128.6, 126.1, 107.0, 77.4, 77.1, 76.8, 16.0. **HRMS (ESI)** m/z $[M+H]^+$ calculated for $C_{10}H_{12}NO$ 162.0913; Found 162.0913.

4.3. Synthesis of α-ketonethiols

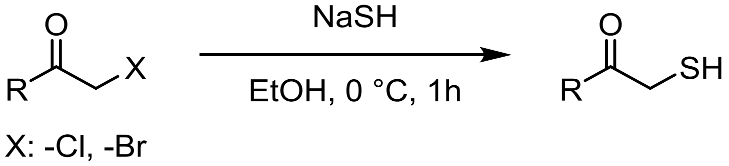

A flask was charged with sodium hydrogensulfide (1.1 Eq.) and dissolved in ethanol (0.65 M); this was left to stir until full dissolution occurred by the formation of a white cloudy solution. The solution was cooled with an ice bath and the α-haloketone (1 Eq,) was added and allowed to stir for 1-1.5 hours. After this time, the ice bath was removed and 20 mL of diethyl ether was added resulting in a precipitate to form. The precipitate was removed by vacuum filtration, washed with ethanol, diethyl ether and further dried under vacuum at 40 °C overnight. The products were used without further purification.

*ethyl 3-mercapto-2-oxopropanoate (**6b**)*

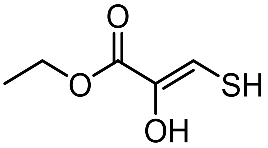

The thiol (isolated in enol-form) was prepared using Ethyl bromopyruvate (**3**, 2000 mg, 1.28 mL, 9.23 mmol, 90 % purity, 1 Eq.) and sodium hydrogensulfide (632.1 mg, 11.28 mmol 1.1 Eq.) forming a white powder (1.2859 g, 94 %); isolated as the enol-form.

^{1}H NMR: (300 MHz, DMSO-d_6) δ 6.95 (s, 1H), 4.14 (p, J = 7.1 Hz, 3H), 3.62 (d, J = 14.1 Hz, 1H), 2.86 (d, J = 14.2 Hz, 1H), 1.19 (q, J = 6.9 Hz, 5H).**^{13}C {^{1}H} NMR:** (75 MHz, DMSO) δ 171.2, 79.8, 77.1, 62.1, 62.0, 56.4, 36.9, 35.6, 14.4. **HRMS (ESI)** m/z $[2M+Na]^+$ calculated for $C_{10}H_{16}O_6S_2Na$ 319.0281; Found 319.0281.

4.4. General Procedure: Conventional heating

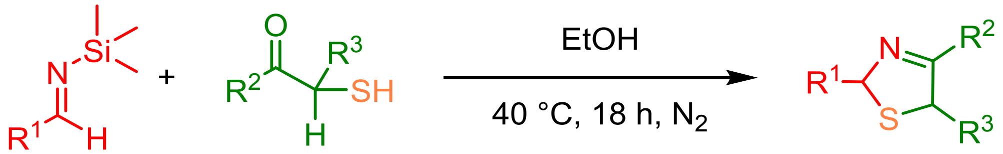

To a flask, α-thiol ketone (1.01-3.40 mmol, 1.50-5.00 Eq.) prepared prior was added and placed under a nitrogen atmosphere. To this, Ethanol (5.0 mL) was added and the contents was left to stir for 5-10 min. To this, N-Trimethylsilyl-imine (0.67 mmol, 1.00 Eq.) was added and left to stir for 18 hours at 40 °C. After the reaction, the content was transferred to a separatory funnel using Ethyl acetate (20 mL) and water (20 mL). The layers were separated and the aqueous layer was once more extracted using ethyl acetate (20 mL). The organic layers were combined and dried by brine (10 mL) and NaSO4, followed by the removal of solvent under reduced pressure. Isolation of the products was performed by flash column chromatography using mixtures of n-heptane in ethyl acetate to afford the corresponding products.

Ethyl 2-phenyl-2,5-dihydrothiazole-4-carboxylate (**7r**)

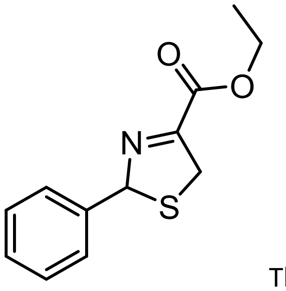

The 3-thiazoline was prepared using commercially available N-Trimethylsilylbenzaldimine (**5a**, 120 mg, 141 µL, 0.677 mmol) and ethyl 3-mercapto-2-oxopropanoate (500.8 mg, 3.38mmol). 3-Thiazoline purification: Automatic Flash column chromatography, 20:1 to7:3 heptane:ethyl acetate afforded a light-orange oil (90.2 mg, 57 %).

R_f: 0.8 in 3:2 n-heptane:ethylacetate. **^{1}H NMR:** (300 MHz, Chloroform-d) δ 7.43 – 7.27 (m, 5H), 6.87 (dd, J = 6.1, 4.2 Hz, 1H), 4.55 – 4.23 (m, 4H), 1.39 (t, J = 7.1 Hz, 3H). **^{13}C {^{1}H} NMR:** (75 MHz, CDCl3) δ 128.7, 128.5, 127.2, 85.1, 62.8, 44.1, 14.1. **HRMS (ESI)** m/z $[M+H]^+$ calculated for $C_{12}H_{14}NO_2S$ 236.0740; Found 236.0739

*5-methyl-2,4-diphenyl-2,5-dihydrothiazole (**7s**)*

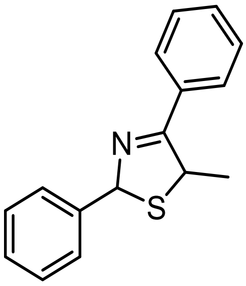

The 3-thiazoline was prepared using commercially available N-Trimethylsilylbenzaldimine (**5a**, 120 mg, 141 µL, 0.677 mmol), 2-bromo-1-phenylpropan-1-one (216 mg, 1.02 mmol) and sodium hydrogensulfide (56.9 mg, 1.02 mmol). 3-Thiazoline purification: Flash column chromatography, 9:1 to 8:2 n-heptane in ethyl acetate afforded a white solid (110.7 mg, 66 %).

R_f: 0.8 in 3:2 n-heptane:ethylacetate. **^{1}H NMR:** (300 MHz, Chloroform-d) δ 8.04 – 7.97 (m, 2H), 7.84 – 7.77 (m, 2H), 7.63 – 7.55 (m, 1H), 7.53 – 7.44 (m, 4H), 7.39 – 7.29 (m, 2H), 4.45 (qd, J = 6.9, 3.9 Hz, 2H), 1.61 (d, J = 6.9 Hz, 3H), 1.44 (d, J = 7.0 Hz, 3H).**^{13}C {^{1}H} NMR:** (75 MHz, CDCl3) δ 128.7, 128.5, 127.2, 85.1, 62.8, 44.1, 14.1. **HRMS (ESI)** m/z $[M+H]^+$ calculated for $C_{16}H_{16}NS$ 254,0998; Found 254,0997.

*2-phenyl-2,5-dihydrothiazole (**7t**)*

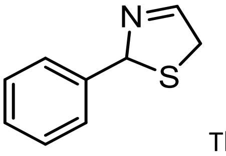

The 3-thiazoline was prepared using commercially available N-Trimethylsilylbenzaldimine (**5a**, 120 mg, 141 µL, 0.677 mmol), 2-chloroacetaldehyde (55 %wt. in water, 145 mg, 117 µL, 1.02 mmol) and sodium hydrogensulfide (56.9 mg, 1.0 mmol). 3-Thiazoline purification: Automatic Flash column chromatography, 6:1 to 3:1 heptane:ethyl acetate afforded a light-yellow oil (64.6 mg, 59 %).

R_f: 0.3 in 3:1 n-heptane:ethylacetate. **^{1}H NMR:** (300 MHz, Chloroform-d) δ 7.60 (dt, *J* = 2.7, 1.4 Hz, 1H), 7.35 – 7.19 (m, 5H), 6.66 (ddd, *J* = 5.9, 3.6, 2.6 Hz, 1H), 4.18 – 3.94 (m, 2H). **^{13}C {^{1}H} NMR:** (75 MHz, CDCl3) δ 161.8, 161.3, 141.6, 128.7, 128.1, 126.8, 85.3, 45.6. **HRMS (ESI)** m/z [M+H]$^+$ calculated for $C_9H_{10}NS$ 164.0528; Found 164.0529.

*ethyl 2-(6-methoxybenzo[d]thiazol-2-yl)thiazole-4-carboxylate (**11b**)*

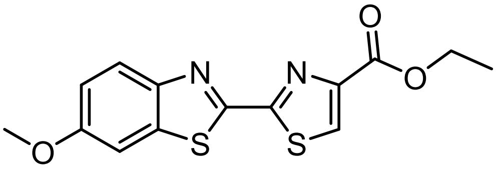

The 3-thiazole was prepared in two consecutive steps. bis(trimethylsilyl)amine (55 mg, 0.34 mmol, 1.1 Eq) and 2.5 M n-butyllithium solution in hexanes (20 mg, 0.120 mL, 0.31 mmol, 1 Eq) were mixed for 20 minutes. The hexanes were removed in vacuo until a white slurry/powder remained, this was dissolved in 1 mL of dry THF and cooled with an ice bath. 6-methoxybenzo[d]thiazole-2-carbaldehyde (**16**, 60.2 mg, 0.312 mmol, 1 Eq) was dissolved in 2 mL of dry THF and added dropwise, forming a clear red solution and was further reacted on room temperature for 20-30 minutes. After this time, Acetic acid (81.3 mg, 77.4 µL, 1.354 mmol, 1 Eq) was added and the solvent was removed under vacuo. The remaining red solid was redissolved in 5 mL of ethanol under nitrogen atmosphere. Ethyl 3-mercapto-2-oxopropanoate (230 mg, 1.55 mmol, 5 Eq.) was added under nitrogen and left to react inside the microwave reactor at 60°C for 30 minutes. The reaction mixture was transferred to a separatory funnel, washed with water, extracted with ethyl acetate and dried with brine and Na_2SO_4. The solvent was removed under vacuo to afford the crude 3-thiazoline as a brown/orange oil. 3-Thiazoline purification: Automatic flash column chromatography, 9:1 to 7:3 heptane: ethyl acetate using a 25-gram SiO_2 flash pure eco-flex cartridge, afforded an orange solid (15 mg, 15 % over 2 steps).

R_f: 0.3 using 10% methanol in DCM. **^{1}H NMR** (300 MHz, Chloroform-*d*) δ 8.29 (s, 1H), 7.98 (d, *J* = 9.0 Hz, 1H), 7.39 (d, *J* = 2.5 Hz, 1H), 7.14 (dd, *J* = 9.0, 2.5 Hz, 1H), 4.47 (q, *J* = 7.1 Hz, 2H), 3.92 (s, 3H), 1.45 (t, *J* = 7.1 Hz, 3H). **^{13}C {^{1}H} NMR** (75 MHz, $CDCl_3$) δ 129.5, 117.1, 104.0, 61.9, 56.0, 14.5. **HRMS (ESI)** m/z [M+H]$^+$ calculated for $C_{14}H_{13}N_2O_3S_2$ 321.0362; Found 321.0375.

4.5 Benzothiazole Precursor Procedure for Luciferin (8) synthesis

*2-bromo-6-methoxybenzo[d]thiazole (**15**)*

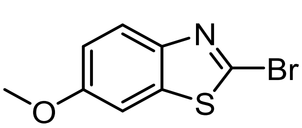

Copper (II) bromide (4.02 g, 18 mmol, 1.2 Eq.) and PEG (M_w: 200 g/mol, 6.3 g) were dissolved in MeCN C (150ml) under nitrogen atmosphere., iso-pentylnitrite (3.02 ml, 2.63 g, 22.5 mmol, 1.5 Eq.) was then added, and the mixture was stirred at room temperature. A separate solution was prepared of 2-amino-6-methoxybenzothiazole (2.70 g, 15 mmol, 1 Eq.) dissolved in MeCN (50ml) with PEG (M_w: 200 g/mol, 6.3 g). This mixture was added dropwise to the former under sonication while under nitrogen over a period of 10 min. The resulting solution was then stirred at 65°C for 2.5 to 3 h under nitrogen atmosphere. After cooling to room temperature, the mixture was poured into a separatory funnel and 1 M HCl Aq. (400 mL) was added. The organics were extracted with chloroform (200 mL), dried with brine and Na_2SO_4. The solvent evaporated in vacuo to dryness. The remaining dark red residue was purified by sublimation under reduced pressure (90-120°C) to give a yellow/red solid (2.550 g, 10.5 mmol, 70 %).

^{1}**H NMR** (300 MHz, Chloroform-d) δ 7.83 (d, J = 9.0 Hz, 1H), 7.23 (d, J = 2.6 Hz, 1H), 7.07 (dd, J = 9.0, 2.6 Hz, 1H), 3.87 (s, 3H). ^{13}**C {^{1}H} NMR**: (75 MHz, Chloroform-d) δ 123.3, 115.8, 103.6, 55.8. Spectra are in agreement with previous reported literature.[4] **HRMS (ESI)** m/z $[M+H]^+$ calculated for C_8H_7BrNOS 243.9426; Found 243.9427.

*6-methoxybenzo[d]thiazole-2-carbaldehyde (**16**)*

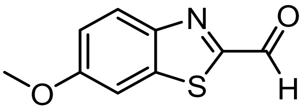

2-bromo-6-methoxybenzo-[d]-thiazole (**15**) (440.3 mg, 1.804 mmol, 1 Eq.) was charged in a flask, placed under nitrogen atmosphere, and dissolved in dried THF (8 mL). After leaving it to stir for 10 minutes, the solution was cooled to -78 °C by a dry ice bath with acetone. After cooling, n-butyllithium (150.2 mg, 937.9 µL, 2.5 molar, 1.3 Eq, 2.345 mmol) was added in small portions. After full addition, the mixture was stirred for an additional 30 minutes. Then, DMF (527.4 mg, 559 µL,7.215 mmol 4 Eq.) was added at once. The solution was allowed to further stir at -78 °C and monitored until consumption of the starting material had occurred, which typically took 1-1.5 h. After consumption of starting material had occurred, the reaction was quenched with saturated ammonium chloride solution at -78 °C and left to vigorously stir for 5 minutes. The mixture was extracted with ethyl acetate and dried with brine and Na_2SO_4. Purification performed by flash column chromatography with 2:1 n-heptane: ethyl acetate afforded a fluffy yellow solid (243.3 mg, 70%).

R_f: 0.5 in 2:1 n-heptane: ethyl acetate ^{1}**H NMR** (300 MHz, Chloroform-d) δ 10.11 (d, J = 0.8 Hz, 1H), 8.11 (d, J = 9.1 Hz, 1H), 7.39 (d, J = 2.5 Hz, 1H), 7.22 (dd, J = 9.1, 2.5 Hz, 1H), 3.93 (s, 3H). ^{13}**C {^{1}H} NMR** (75 MHz, chloroform-d) δ 185.3, 126.7, 118.5, 103.8, 56.1. **HRMS (ESI)** m/z $[M+H]^+$ calculated for C_9H_8NO2S 194.0270; Found 194.0271.

4.6. General Procedure 2-thiazoline and Luciferin Synthesis

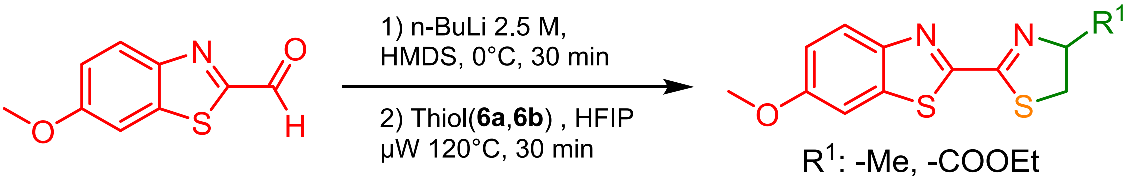

A flask was put under nitrogen, HMDS (1.1 Eq) was added and cooled to 0°C using an ice bath. Then n-BuLi (2.5 M, 1.0 Eq.) was added dropwise and allowed to stir for 10 minutes and diluted with dry THF (1.5 mL). 6-methoxybenzo[d]thiazole-2-carbaldehyde (**16,** 1 Eq.) was dissolved in THF (1.5 mL) and added dropwise to the former solution, this was allowed to stir for 30 min at 0°C. The mixture was quenched with acetic acid (1.0 Eq.) and the residual solvent removed under reduced pressure and redissolved in HFIP (2.5 mL, 0.13 M). Either pre- or in-situ made thiol was added and the flask purged with nitrogen again, and placed under microwave heating at 120 °C for 30 min. The mixture was extracted using ethyl acetate, dried with brine and Na_2SO_4 and the solvent was removed under reduced pressure. Final purification was achieved by column chromatography.

*6-methoxy-2-(4-methyl-4,5-dihydrothiazol-2-yl)benzo[d]thiazole (**10**)*

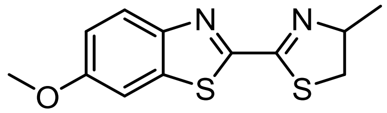

The imine intermediate was prepared using 6-methoxybenzo[d]thiazole-2-carbaldehyde (**16**) (60 mg, 0.31 mmol, 1 Eq.), 2.5 M n-butyllithium solution (0.31 mmol, 0.120 mL, 1 Eq.) and HMDS (55 mg, 71 µL, 0.34 mmol, 1.1 Eq.). Remaining n-butyllithium quenched with acetic acid (19 mg, 18 µL, 0.31 mmol 1 Eq.). The 2-thiazoline was made using sodium hydrogensulfide (26 mg, 0.46 mmol, 1.5 Eq.) and chloroacetone (43 mg, 37 µL, 0.46 mmol, 1.5 Eq.). 2 Thiazoline purification was performed by Automatic flash column chromatography, 9:1 to 7:3 heptane:ethyl acetate using a 25-gram SiO_2 flashpure eco-flex cartridge, afforded an orange solid (33 mg, 40 %).

R_f: 0.3 in 1:1 n-heptane:ethylacetate. **^{1}H NMR** (300 MHz, Chloroform-d) δ 8.02 (d, J = 9.0 Hz, 1H), 7.35 (d, J = 2.5 Hz, 1H), 7.13 (dd, J = 9.1, 2.5 Hz, 1H), 5.35 (dd, J = 9.6, 8.8 Hz, 1H), 4.31 (q, J = 7.1 Hz, 2H), 3.90 (s, 3H), 3.82 – 3.63 (m, 1H), 1.35 (t, J = 7.1 Hz, 3H). **^{13}C {^{1}H} NMR** (75 MHz, $CDCl_3$) δ 125.3, 116.9, 103.7, 78.4, 62.02, 55.8, 35.3, 35.3, 14.2. **HRMS (ESI)** m/z $[M+H]^+$ calculated for $C_{12}H_{13}N_2OS_2$ 265.0464; found 265.0462.

ethyl 2-(6-methoxybenzo[d]thiazol-2-yl)-4,5-dihydrothiazole-4-carboxylate (12)

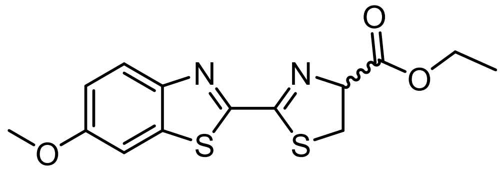

The imine intermediate was prepared using 6-methoxybenzo[d]thiazole-2-carbaldehyde (**16**) (250 mg, 1.29 mmol, 1 Eq.), 2.5 M n-butyllithium solution (1.29 mmol, 0.518 mL, 1 Eq.) and HMDS (230 mg, 297 µL, 1.42 mmol, 1.1 Eq.). Remaining n-butyllithium was quenched with acetic acid (77.7 mg, 74.1 µL, 1.29 mmol 1 Eq.). The 2-thiazoline was made using pre-made ethyl 3-mercapto-2-oxopropanoate (955 mg, 6.45 mmol 5 Eq.) 2-Thiazoline purification was performed by Automatic flash column chromatography, 6:1 to 1:1 heptane: ethyl acetate using a 25-gram SiO_2 flashpure eco-flex cartridge, afforded a yellow solid (165 mg, 40 %).

R_f: 0.3 using 10 % methanol in DCM. **^{1}H NMR** (300 MHz, Chloroform-*d*) δ 8.02 (d, *J* = 9.0 Hz, 1H), 7.34 (d, *J* = 2.5 Hz, 1H), 7.13 (dd, *J* = 9.0, 2.6 Hz, 1H), 5.35 (dd, *J* = 9.6, 8.8 Hz, 1H), 4.31 (q, *J* = 7.1 Hz, 2H), 3.90 (s, 3H), 3.74 (qd, j = 11.3, 9.2 Hz, 2H), 1.35 (t, *J* = 7.1 Hz, 3H). **^{13}C {^{1}H} NMR** (75 MHz, $CDCl_3$) δ 125.4, 117.0, 103.8, 78.5, 77.6, 77.2, 76.7, 62.2, 56.0, 35.4, 14.3. **HRMS (ESI)** m/z $[M+H]^+$ calculated for $C_{14}H_{15}N_2O_3S_2$ 323.0519; Found 323.0520.

2-(6-hydroxybenzo[d]thiazol-2-yl)-4,5-dihydrothiazole-4-carboxylic acid ((±) luciferin) (8)

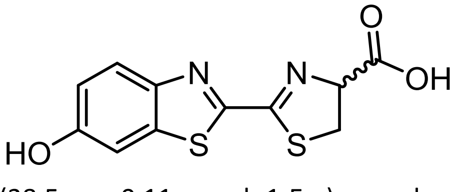

The deprotection was performed in two consecutive steps. **12** (38.5 mg, 0.11 mmol, 1 Eq.) was placed under nitrogen and dissolved in dry DCM (0.25 M), this was followed by cooling the solution using an ice bath for 10 minutes. BBr_3 in DCM (1 M, 0.46 mL, 0.46 mmol, 4.2 Eq.) was added and left to stir overnight to room temperature. The reaction was quenched with 1 M HCl $_{aq}$, extracted with ethyl acetate, dried with brine and Na_2SO_4. The solvent was removed

under reduced pressure resulting in a dark brown solid. This was redissolved in phosphate buffer and MeCN (80:20 v/v, 0.1 M Phosphate buffer pH 8.0) to a concentration of 2 mg/mL of crude substrate. Porcine Liver esterase (0.05 mg enzyme per 1 mg of substrate) was added. This mixture incubated at 37°C for 24 hours. After this time the mixture was freeze-dried. The resulting solids were triturated with MeCN & water. Final purification was performed by Preparatory HPLC resulting in a white powder (9.9 mg, 30 %).

^{1}H NMR (300 MHz, DMSO-d_6) δ 7.96 (d, J = 8.9 Hz, 1H), 7.45 (d, J = 2.4 Hz, 1H), 7.06 (dd, J = 8.9, 2.4 Hz, 1H), 5.40 (dd, J = 9.7, 8.3 Hz, 1H), 3.84 – 3.55 (m, 2H). **^{13}C {^{1}H} NMR** (75 MHz, DMSO) δ 171.7, 164.8, 157.9, 157.2, 146.7, 137.7, 125.3, 117.6, 107.3, 78.6, 35.1. Spectra in agreement with previously reported literature for D/L-Luciferin.[5,6] **ee%**: 70% towards L-Luciferin **HRMS (ESI)** m/z $[M+H]^+$ calculated for $C_{11}H_9N_2O_3S_2$ 281.0050; Found 281.0056

4.7. L-luciferin 2-thiazoline reference material synthesis

6-methoxybenzo[d]thiazole-2-carbonitrile (17)

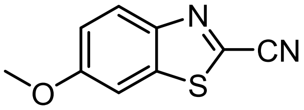

Sodium cyanide (240 mg, 3.69 mmol, 3 Eq.) was added to a microwave vail and purged with nitrogen and dry DMSO (12 mL, 0.1 M) was added, the salt was dissolved under microwave irradiation at 120 °C for 5 minutes. Afterwards 2-bromo-6-methoxybenzo-[d]-thiazole (**15**) (300 mg, 1.23 mmol, 1 Eq.) was added and allowed to dissolve. Then, the mixture was heated by microwave irradiation at 120°C for 20 minutes and conversion verified by TLC. The mixture was transferred to a separatory funnel and diluted with water, the organics extracted with ethyl acetate, washed with brine and dried over sodium sulfate. The mixture was filtered and the solvent removed under reduced pressure. Purification was performed by flash column chromatography using 4:1 heptane:ethyl acetate as eluent, resulting in an off-white powder (189.7 mg, 81 %).

R_f: 0.2 in 4:1 ethyl acetate:heptane. **^{1}H NMR** (300 MHz, Chloroform-d) δ 8.08 (d, J = 9.1 Hz, 1H), 7.36 (d, J = 2.5 Hz, 1H), 7.28 – 7.19 (m, 1H), 3.93 (s, 3H). **^{13}C {^{1}H} NMR:** (75 MHz, CDCl3) δ 126.0, 118.7, 103.1, 56.1. Spectra are in agreement with previous reported literature.[4]

ethyl 2-(6-methoxybenzo[d]thiazol-2-yl)-4,5-dihydrothiazole-4-carboxylate reference (NMR-Reference, 13)

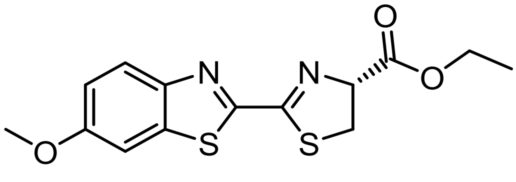

6-methoxybenzo[d]thiazole-2-carbonitrile (**17**) (155.1 mg, 0.815 mmol, 1 Eq.) and L-Cysteine ethyl ester hydrochloride (166.5 mg, 0.896 mmol, 1.1 Eq.) were added to a flask and placed under nitrogen atmosphere. The solids were dissolved in 2:1 v/v MeOH: H_2O (bubbled with nitrogen for 30 minutes, 8.2 mL, 0.1 M) for 10 minutes. In a separate vail, potassium carbonate (127 mg, 0.919 mmol, 1.13 Eq.) was dissolved in 0.5 mL of the pre-mentioned solvent mixture and added dropwise to the other flask. This was left to react until full conversion was verified by TLC, generally within 1-1.5 hr. The contents were transferred to a separatory funnel and acidified using 1 M HCl $_{(aq.)}$ and the organics extracted with ethyl acetate, dried with brine and sodium sulfate, filtered and the solvent removed under reduced pressure, resulting in a yellow solid (198 mg, 75%)

R_f: 0.3 using 10% methanol in DCM **^{1}H NMR**: (300 MHz, Chloroform-d) δ 8.02 (d, J = 9.0 Hz, 1H), 7.92 (d, J = 9.1 Hz, 1H), 7.34 (dd, J = 4.2, 2.6 Hz, 1H), 7.13 (dt, J = 9.1, 2.6 Hz, 1H), 5.35 (dd, J = 9.6, 8.8 Hz, 1H), 4.31 (q, J = 7.1 Hz, 1H), 3.90 (s, 3H), 3.74 (qd, J = 11.3, 9.2 Hz, 2H), 1.35 (t, J = 7.1 Hz, 3H). Luciferin core in agreement with previous reported literature on L-luciferin.[6] **^{13}C {^{1}H} NMR:** (75 MHz, $CDCl_3$) δ 159.2, 125.7, 125.3, 117.7, 116.9, 103.7, 103.4, 78.4, 62.0, 55.8, 35.3, 35.3, 29.7, 14.2. **HRMS (ESI)** m/z $[M+H]^+$ calculated for $C_{14}H_{15}N_2O_3S_2$ 323.0519; Found: 323.0518.

5. Isomerisation Experiments

7a Isomerisation in HFIP

Isomerisation of **7a** in HFIP at 120°C, 30 min

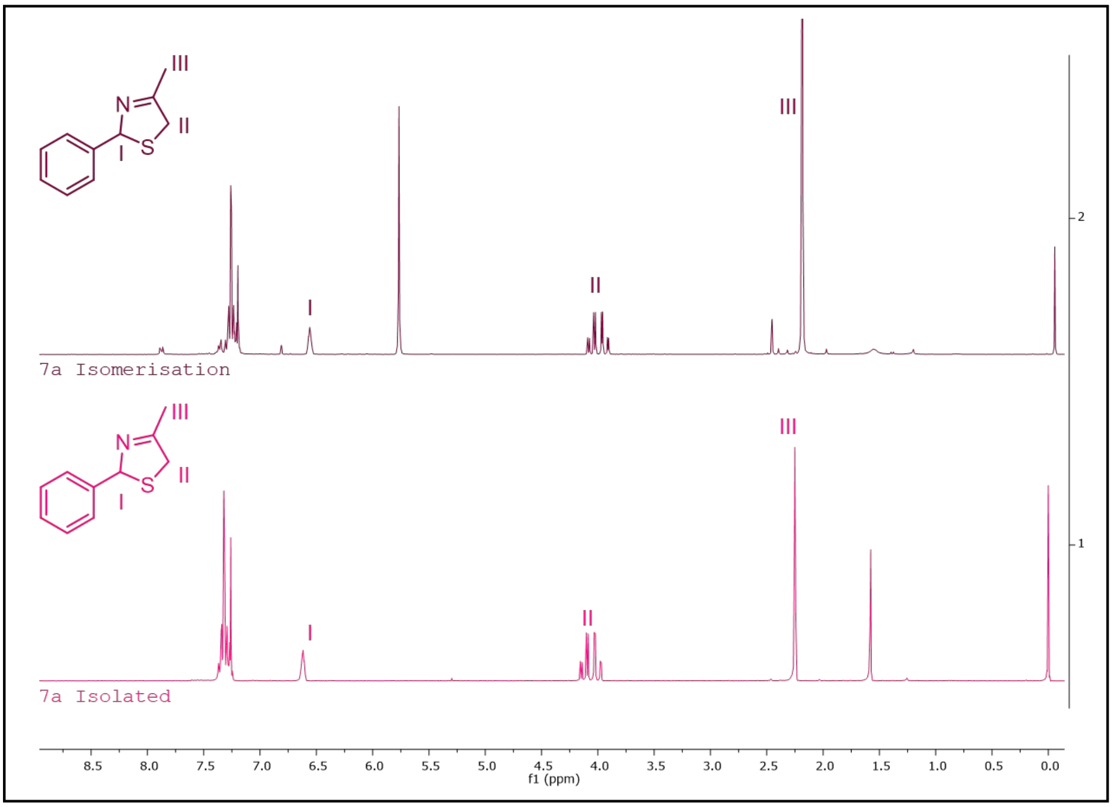

Conditions: 3-thiazoline in HFIP (0.1 M) under nitrogen atmosphere irradiated by microwave to 120°C for 30 minutes.

Result: no visible changes observed

7d Isomerisation HFIP

Isomerisation of **7d** in HFIP at 120°C, 30 min

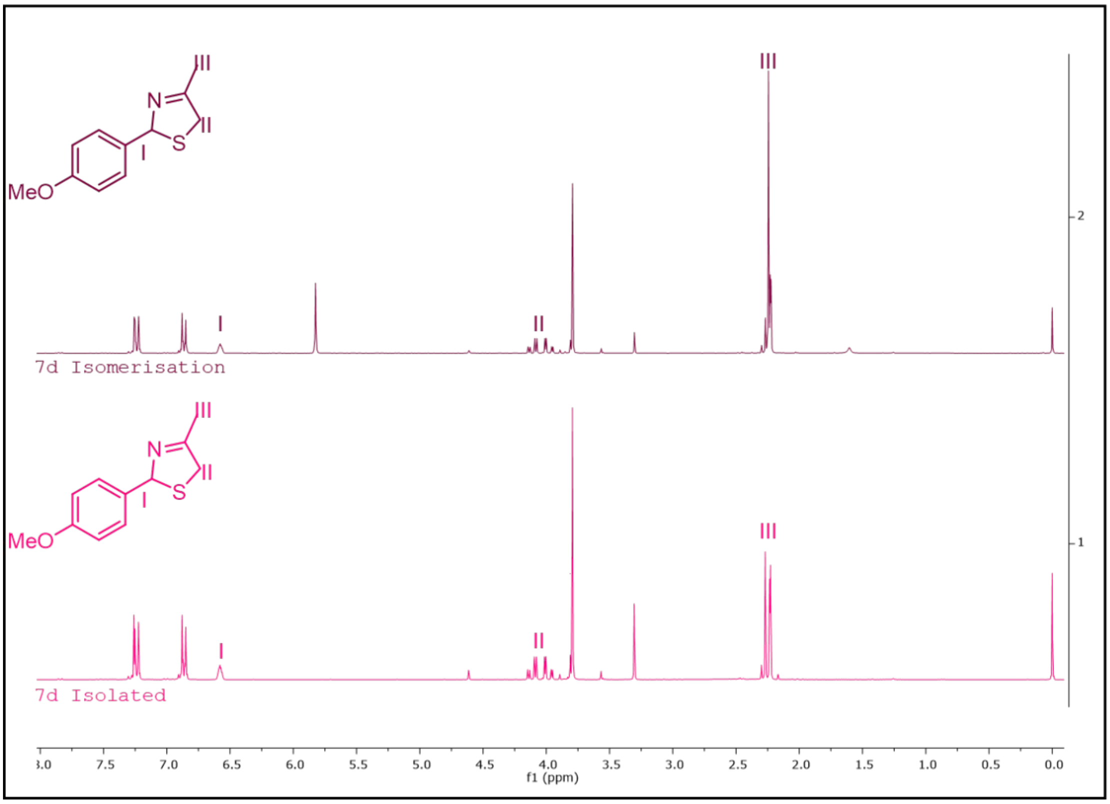

Conditions: 3-thiazoline in HFIP (0.1 M) under nitrogen atmosphere irradiated by microwave to 120°C for 30 minutes.

Result: no visible changes observed

7h Isomerisation HFIP

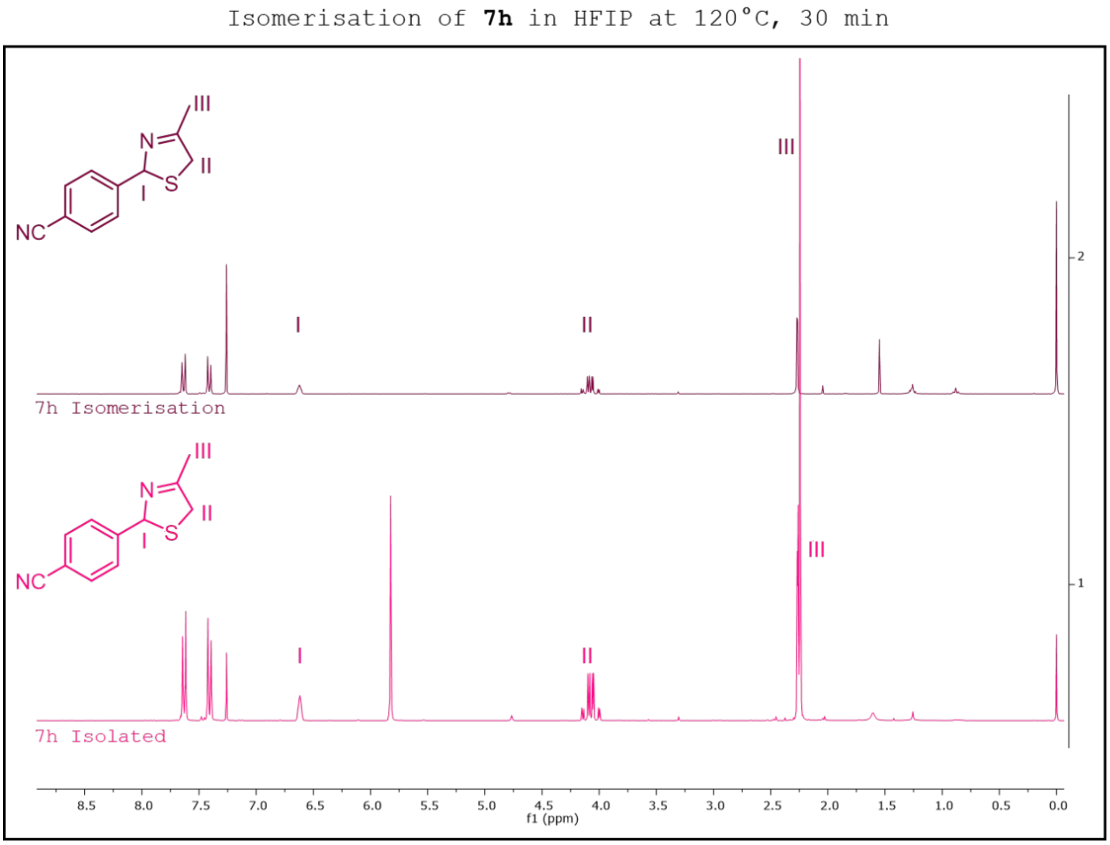

Conditions: 3-thiazoline in HFIP (0.1 M) under nitrogen atmosphere irradiated by microwave to 120°C for 30 minutes.

Result: no visible changes observed

7l isomerisation HFIP

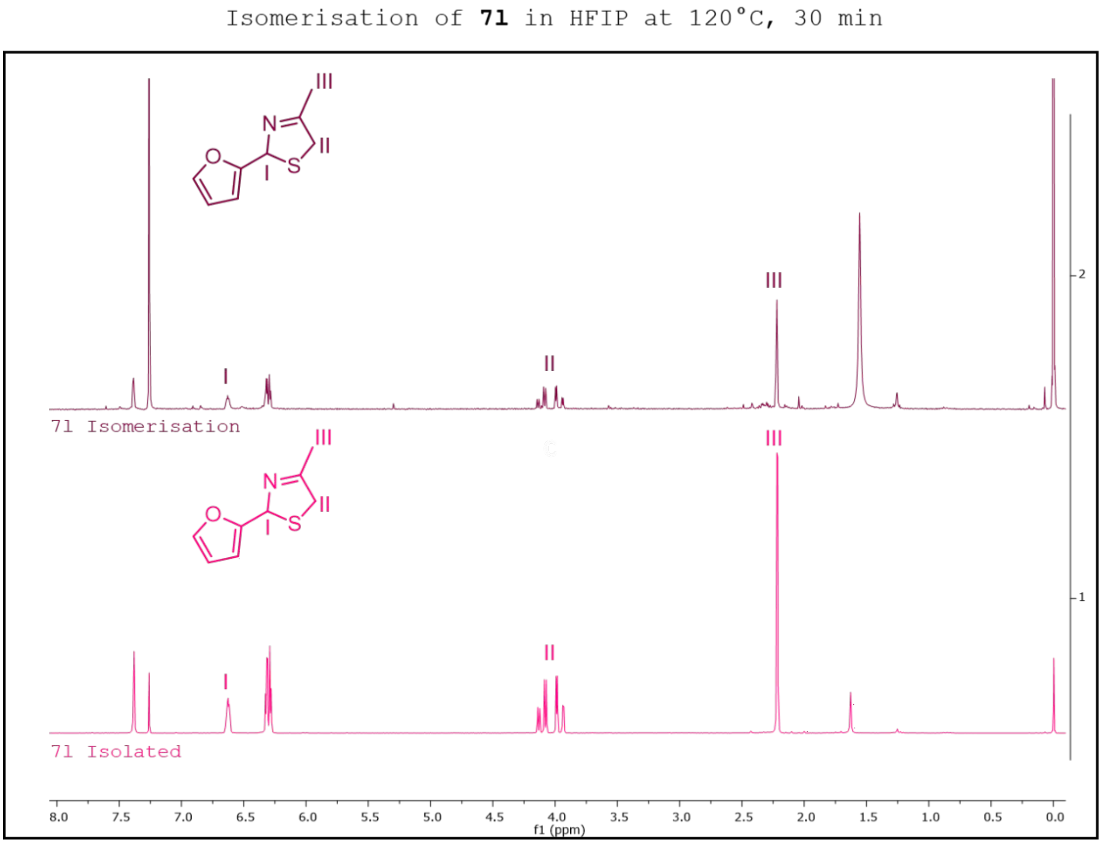

Conditions: 3-thiazoline in HFIP (0.1 M) under nitrogen atmosphere irradiated by microwave to 120°C for 30 minutes.

Result: no visible changes observed

7m Isomerisation HFIP

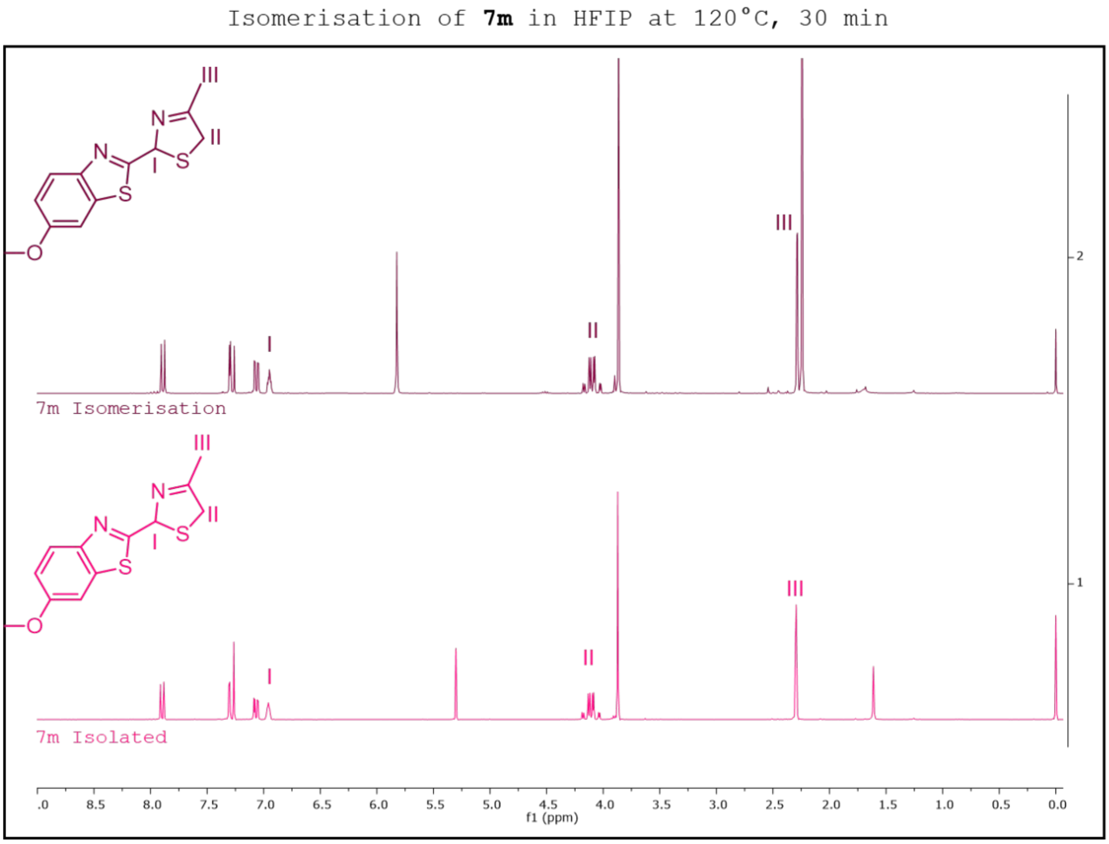

Conditions: 3-thiazoline in HFIP (0.1 M) under nitrogen atmosphere irradiated by microwave to 120°C for 30 minutes.

Result: no visible changes observed

7m Asinger Reaction in HFIP vs. Ethanol

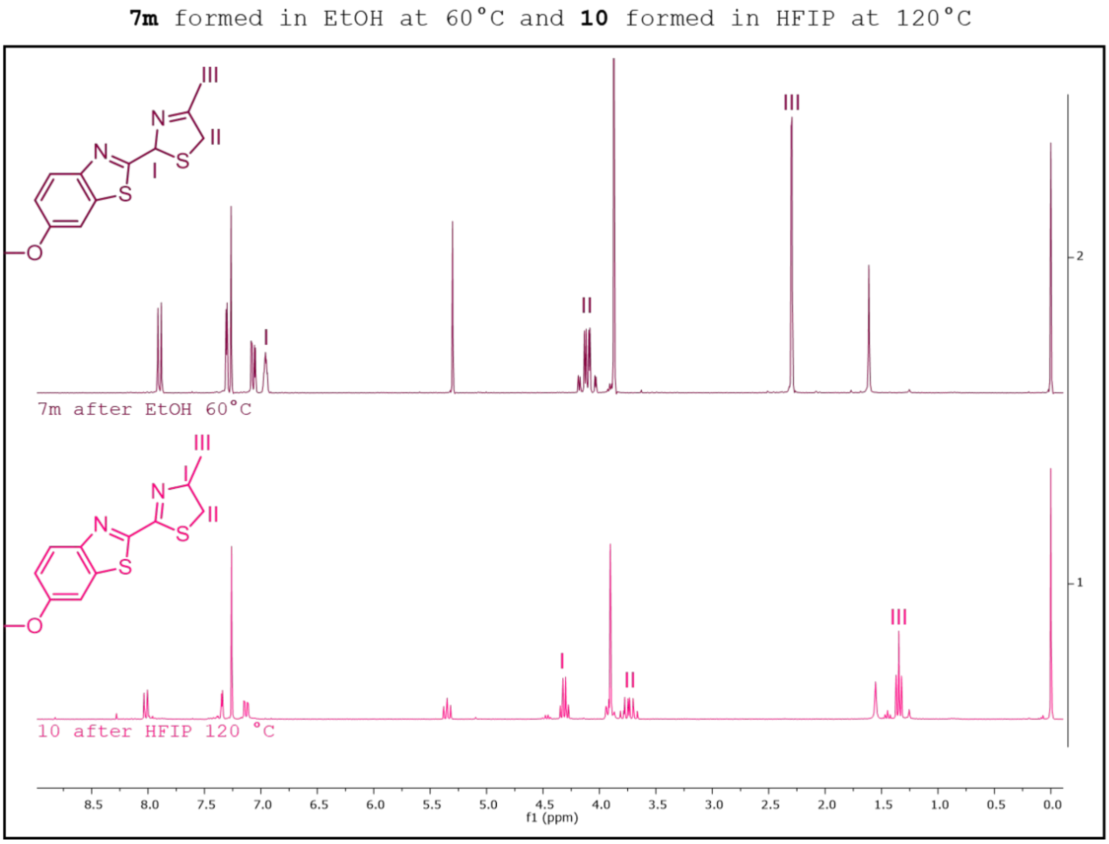

Conditions:

Top: NaSH, chloroacetone and **6m** in EtOH (0.1 M) under nitrogen atmosphere irradiated by microwave to 60°C for 30 minutes.

Bottom: NaSH, chloroacetone and **6m** in HFIP (0.1 M) under nitrogen atmosphere irradiated by microwave to 120°C for 30 minutes.

Result:

Top: 3-thiazoline compound observed and isolated (**7m**)

Bottom: 2-thiazoline compound observed and isolated (**10**)

7m prolonged heating to 10

Isomerisation of **7m** to **10** at various temperatures and time

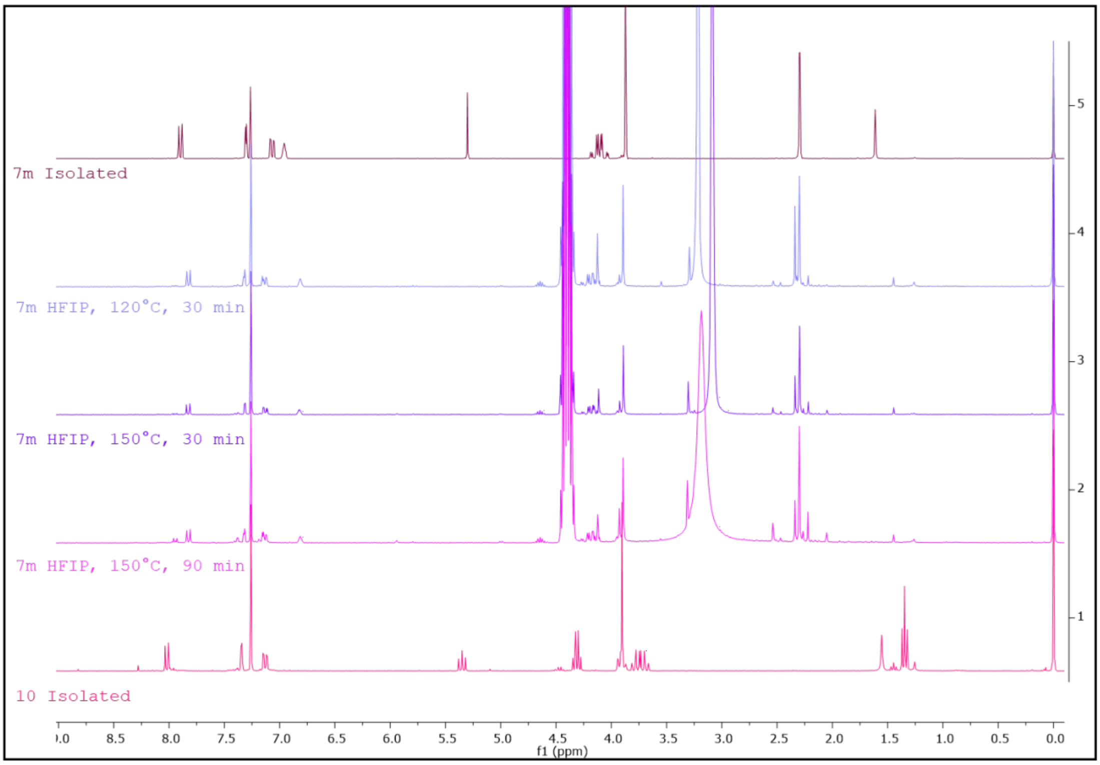

Conditions: Isolated **7m**, dissolved in HFIP (0.1 M) under nitrogen atmosphere. Times and temperature as indicated above. Heating performed by Microwave irradiation.

Result: over time and temperature no conversion of 3-thiazoline (**7m**), to 2-thiazoline (**10**) was observed.

7m Isomerisation to 10 with TMS-Cl

Isomerisation of **7m** to **10** in HFIP at 120°C with 1 equiv of TMS-Cl

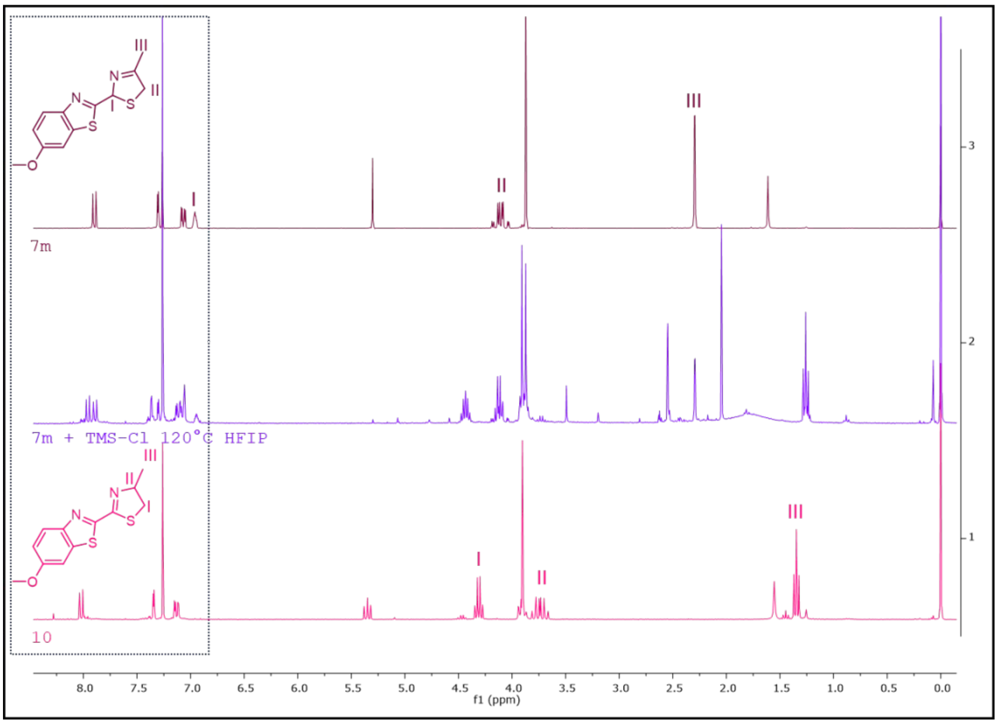

Zoomed in Section between 8.5-7.2 ppm

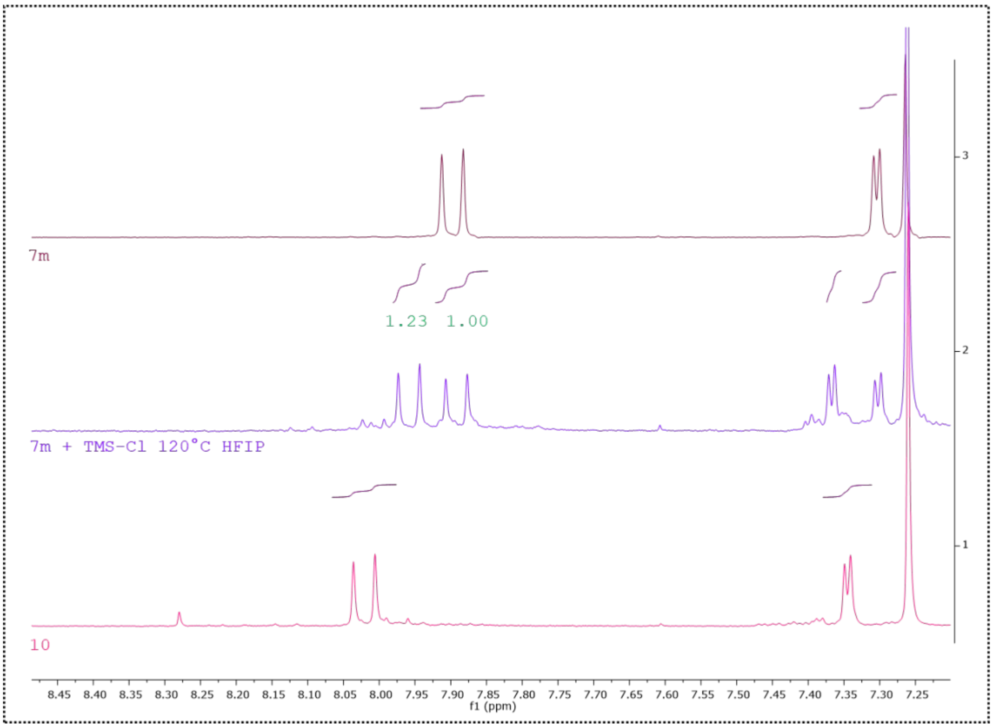

Conditions: Isolated **7m**, dissolved in HFIP (0.1 M) under nitrogen atmosphere, 1 equiv. of TMS-Cl was added and heated to 120°C for 30 min.

Result: over time 55 % conversion of 3-thiazoline (**7m**), to 2-thiazoline (**10**) was observed based on integration.

6. Spectral Data TMS-Imines

5a ^{1}H

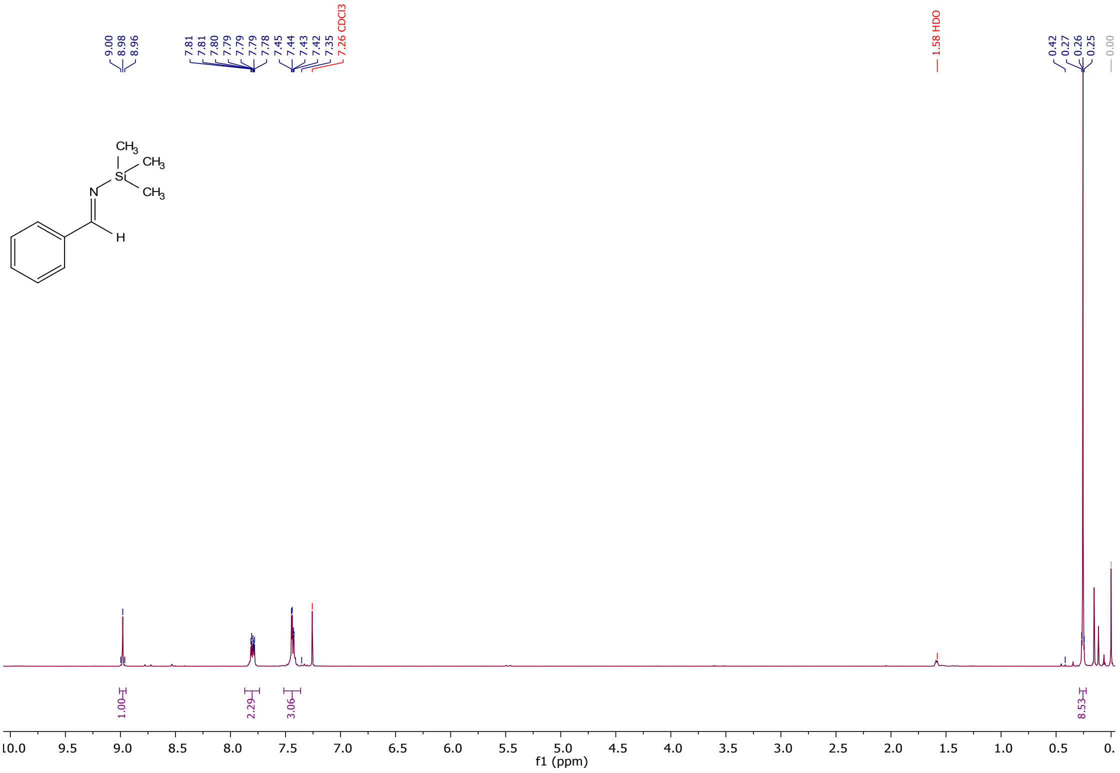

^{1}H NMR Spectra of **5a, 300 MHz (CDCl$_3$)**

5a $^{13}C\ \{^{1}H\}$

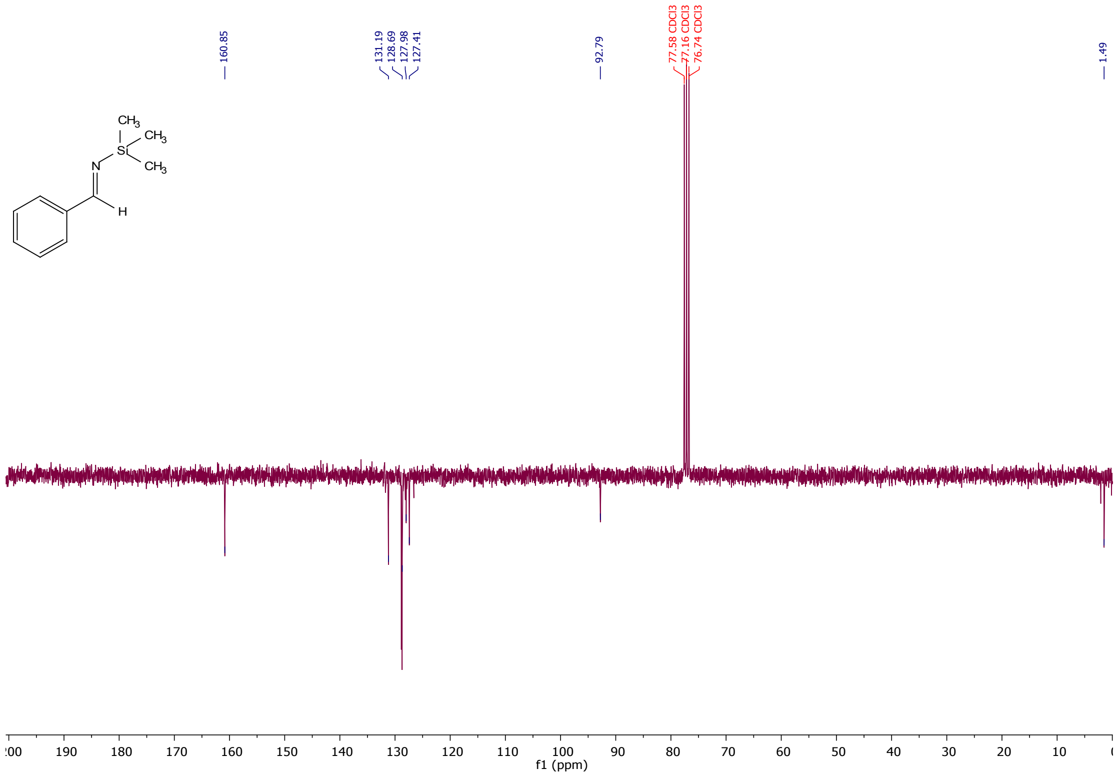

$^{13}C\{^1H\}$ NMR Spectra of **5a, 75 MHz** **($CDCl_3$)**

5b ^1H

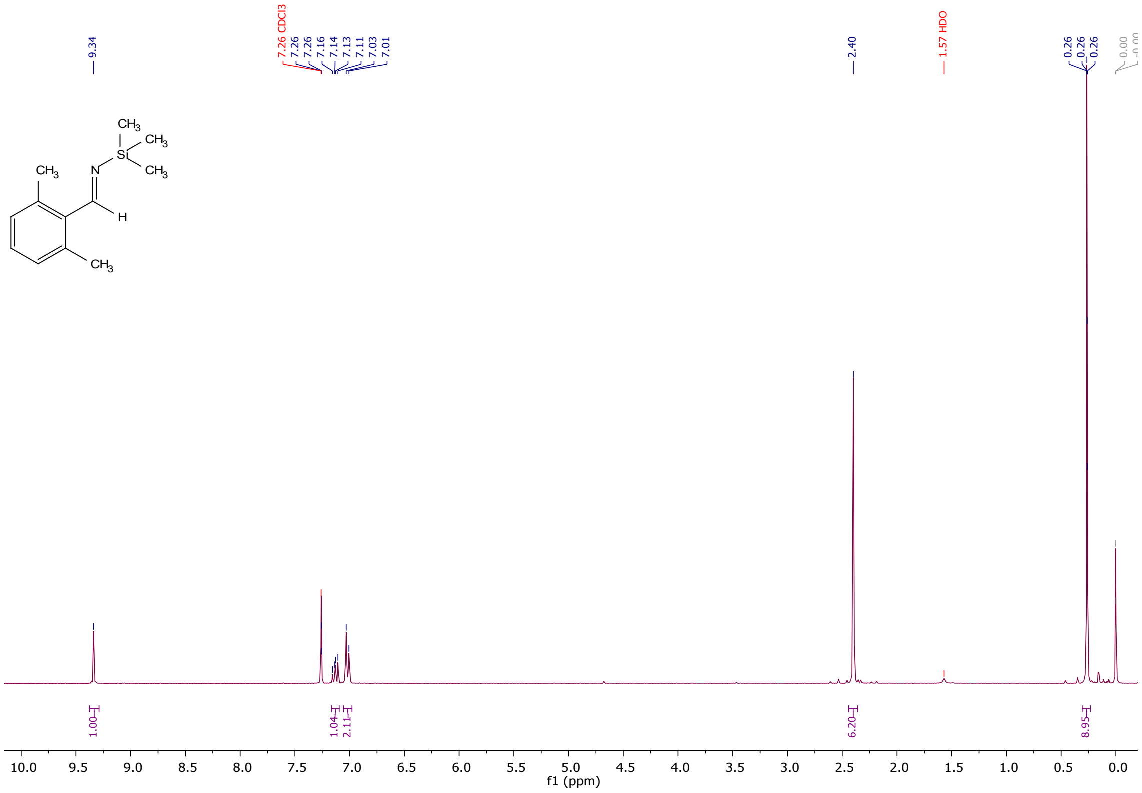

^1H NMR Spectra of **5b, 300 MHz** **($CDCl_3$)**

5b ^{13}C $\{^1H\}$

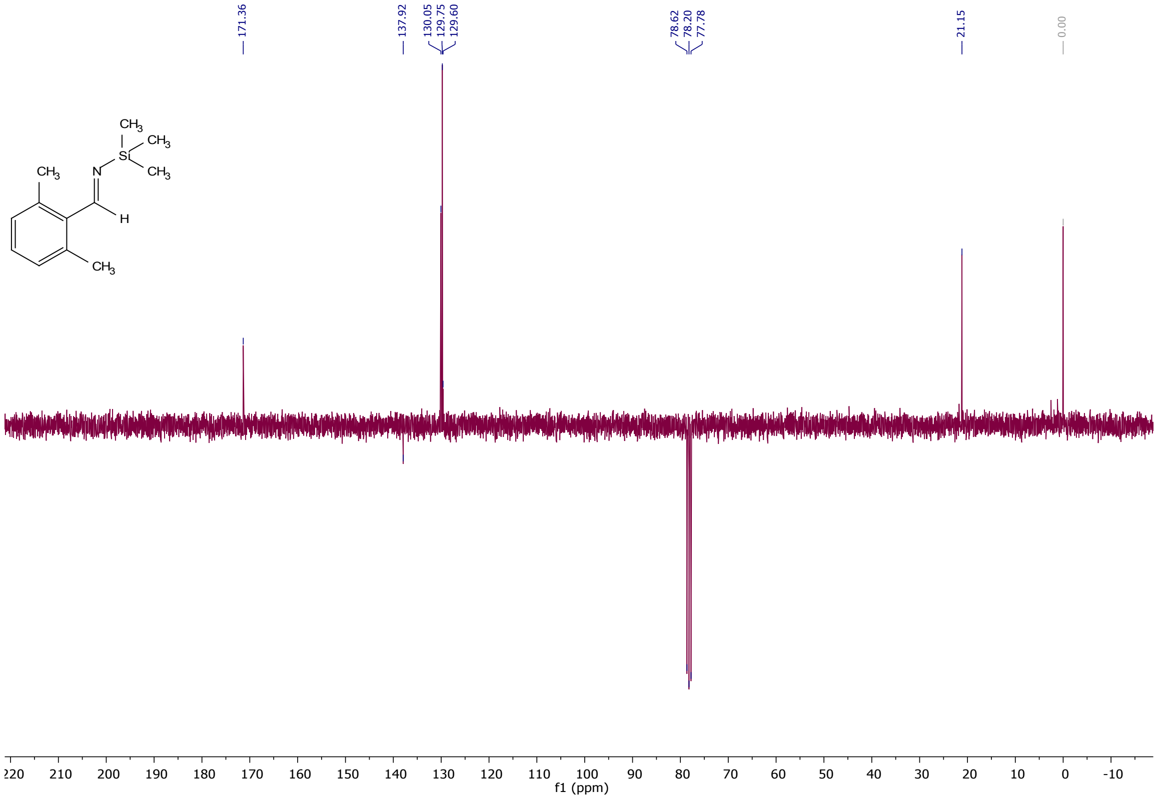

^{13}C {^{1}H} NMR Spectra of **5b,75 MHz (CDCl$_3$)**

5c ^{1}H

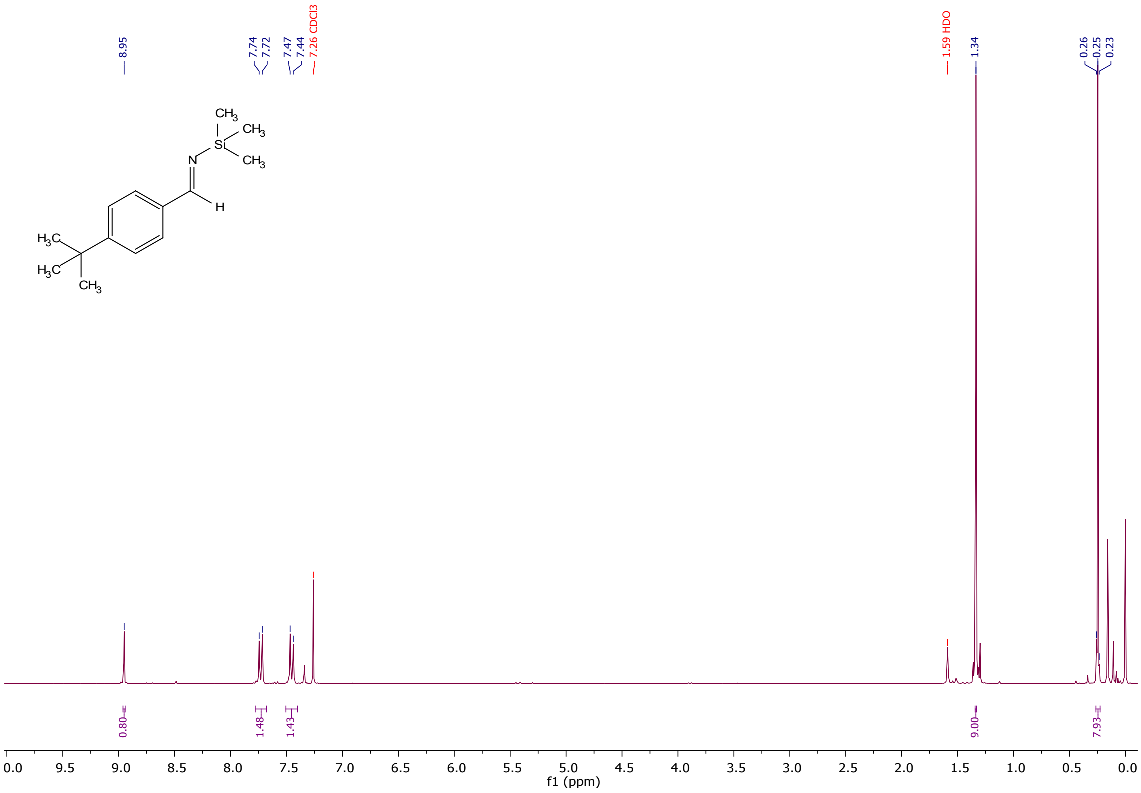

^{1}H NMR Spectra of **5c, 300 MHz (CDCl$_3$)**

5c ^{13}C {^{1}H}

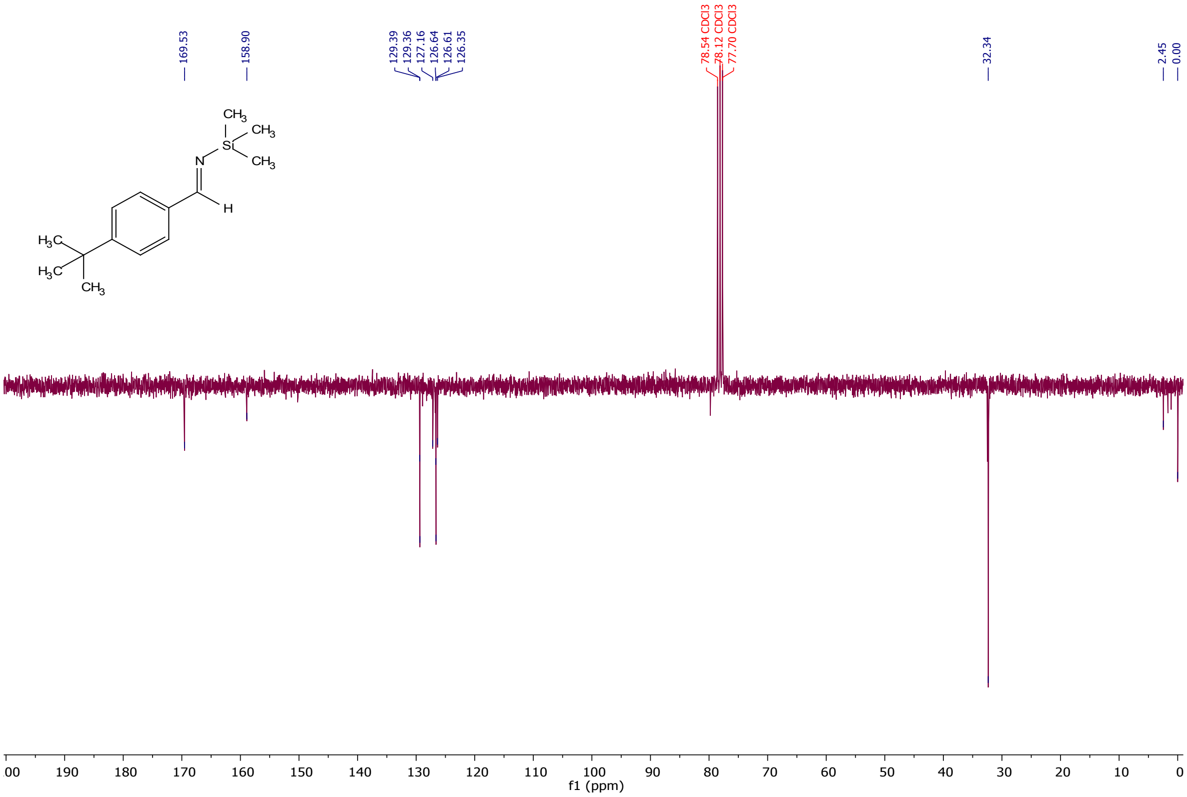

^{13}C {^{1}H}NMR Spectra of **5c,75 MHz ($CDCl_3$)**

5d ^{1}H

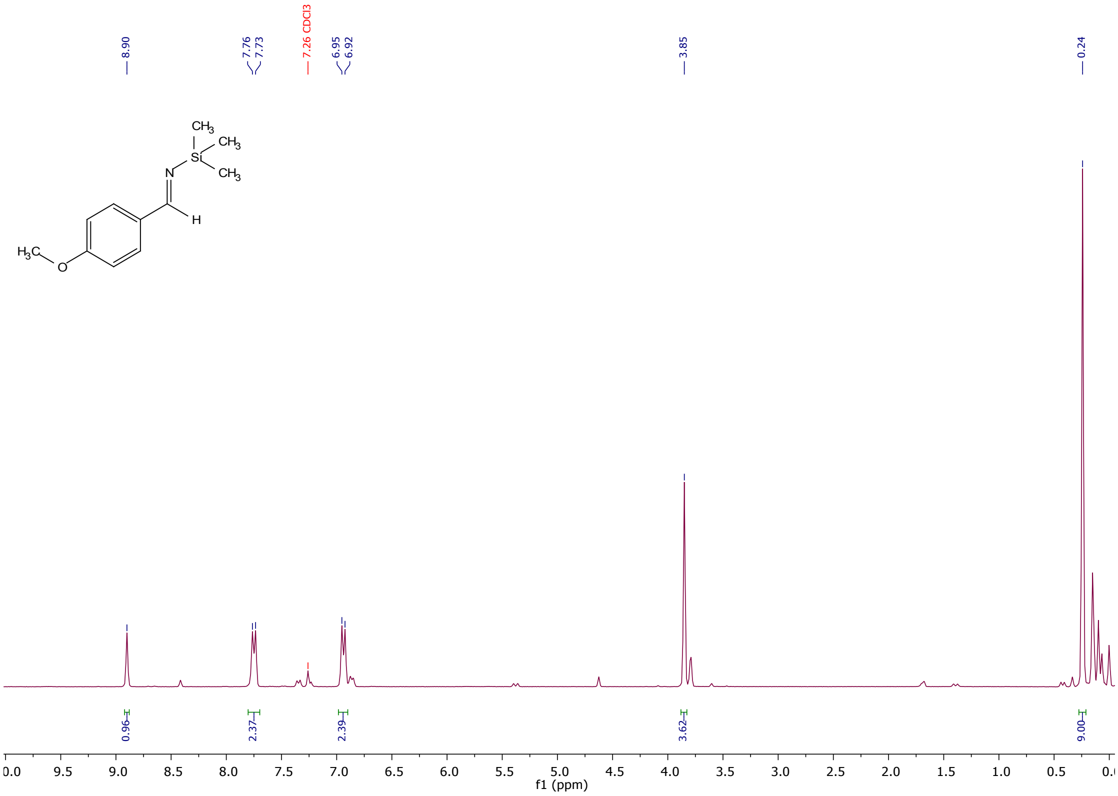

^{1}H NMR Spectra of **5d, 300 MHz (CDCl$_3$)**

5d ^{13}C {^{1}H}

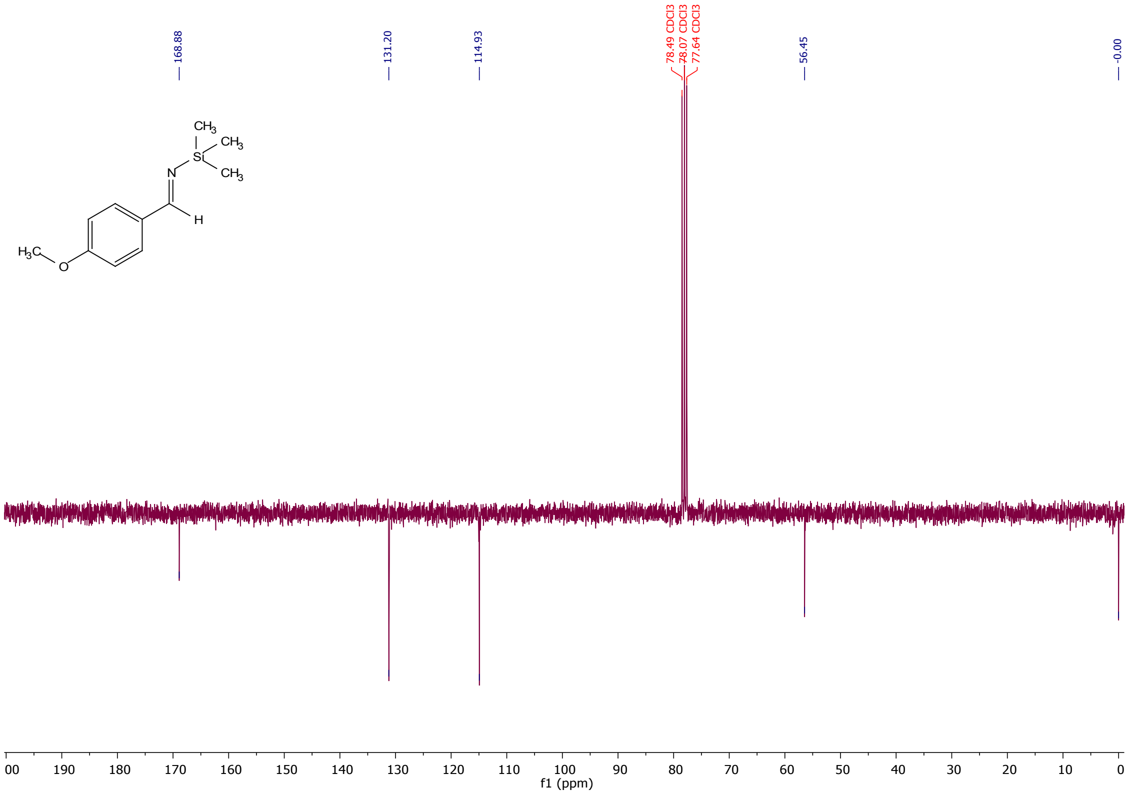

^{13}C {^{1}H} NMR Spectra of **5d,75 MHz (CDCl$_3$)**

5e ^{1}H

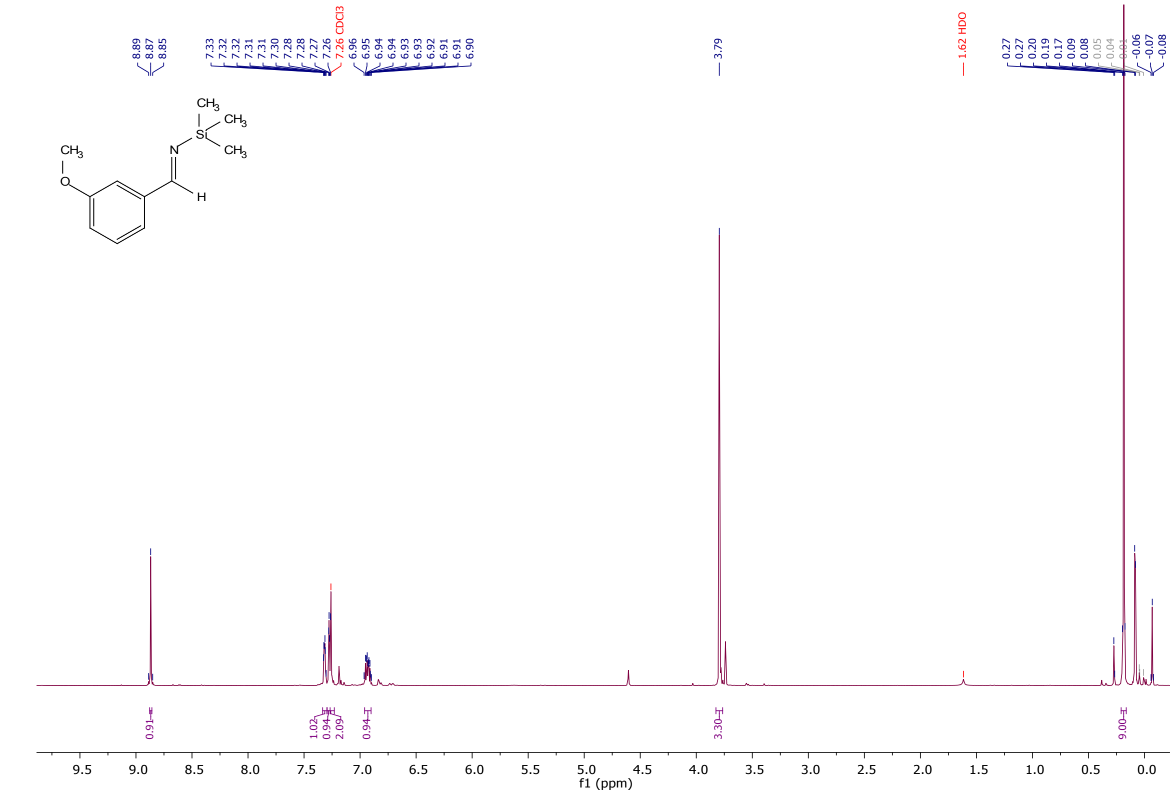

^{1}H NMR Spectra of **5e, 300 MHz (CDCl$_3$)**

5e ^{13}C {^{1}H}

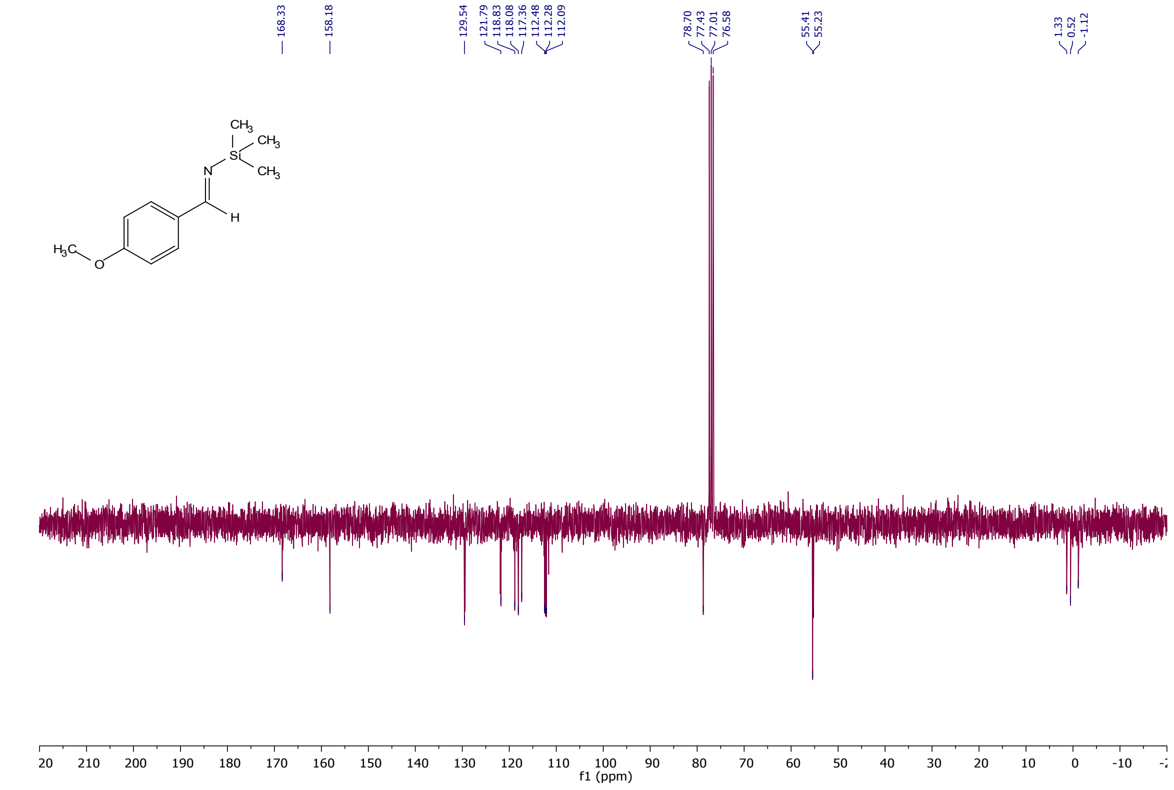

^{13}C {^{1}H} NMR Spectra of **5e,75 MHz (CDCl$_3$)**

5f ^{1}H

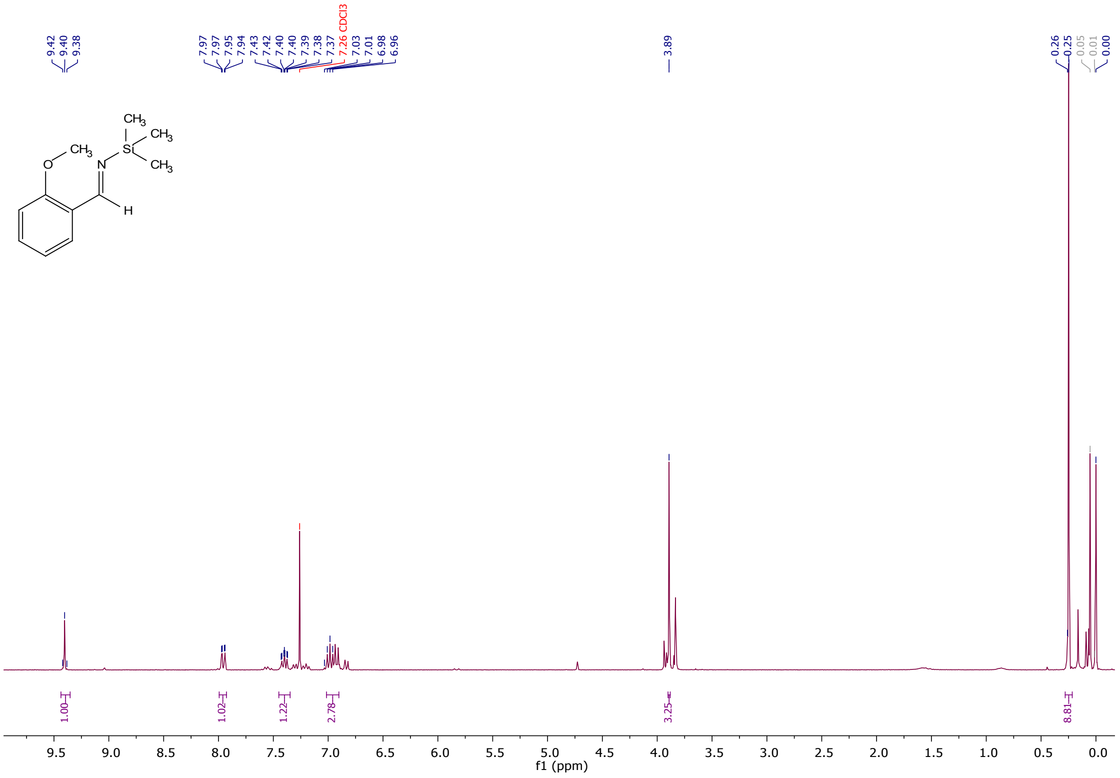

^{1}H NMR Spectra of **5f, 300 MHz (CDCl$_3$)**

5f ^{13}C {^{1}H}

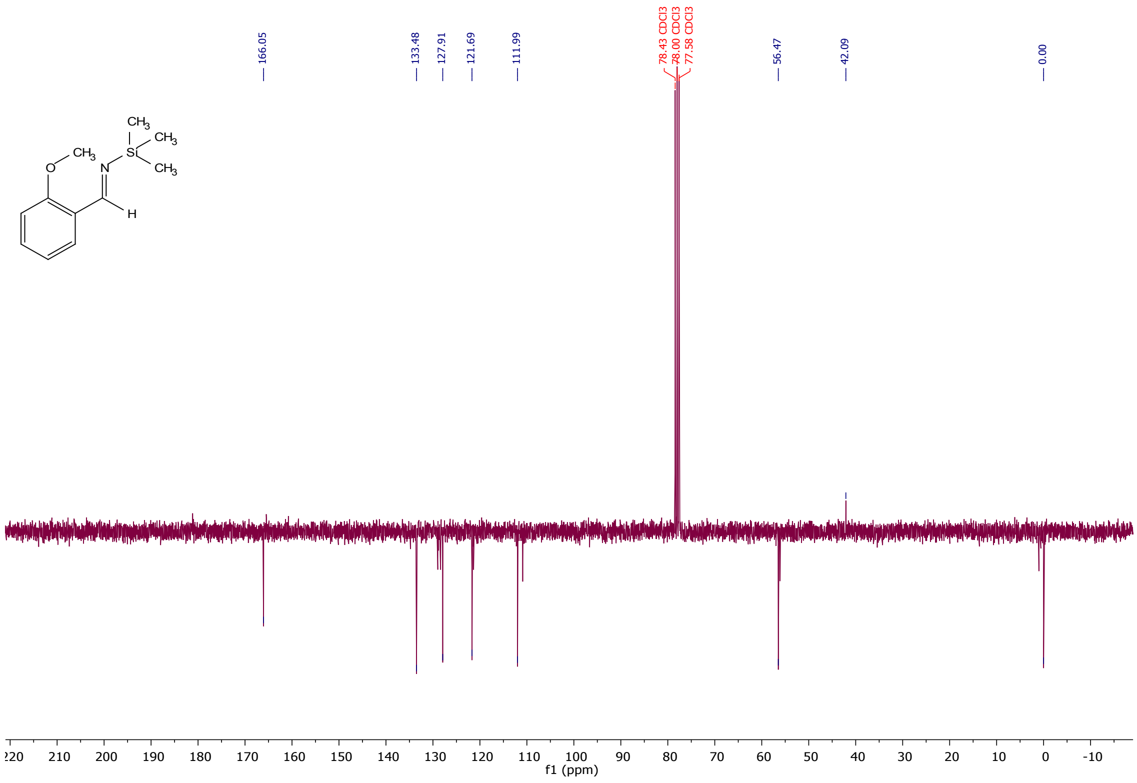

^{13}C {^{1}H} NMR Spectra of **5f,75 MHz (CDCl$_3$)**

5g ^{1}H

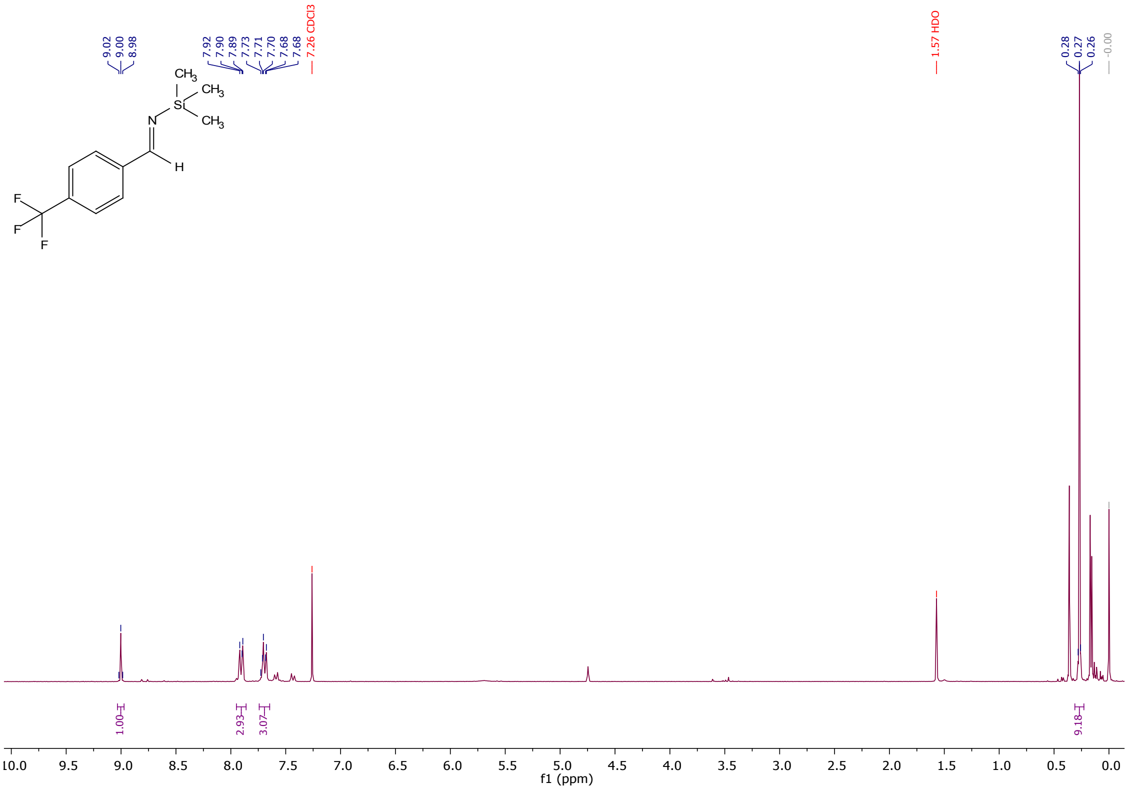

^{1}H NMR Spectra of **5g, 300 MHz (CDCl$_3$)**

5g ^{13}C {^{1}H}

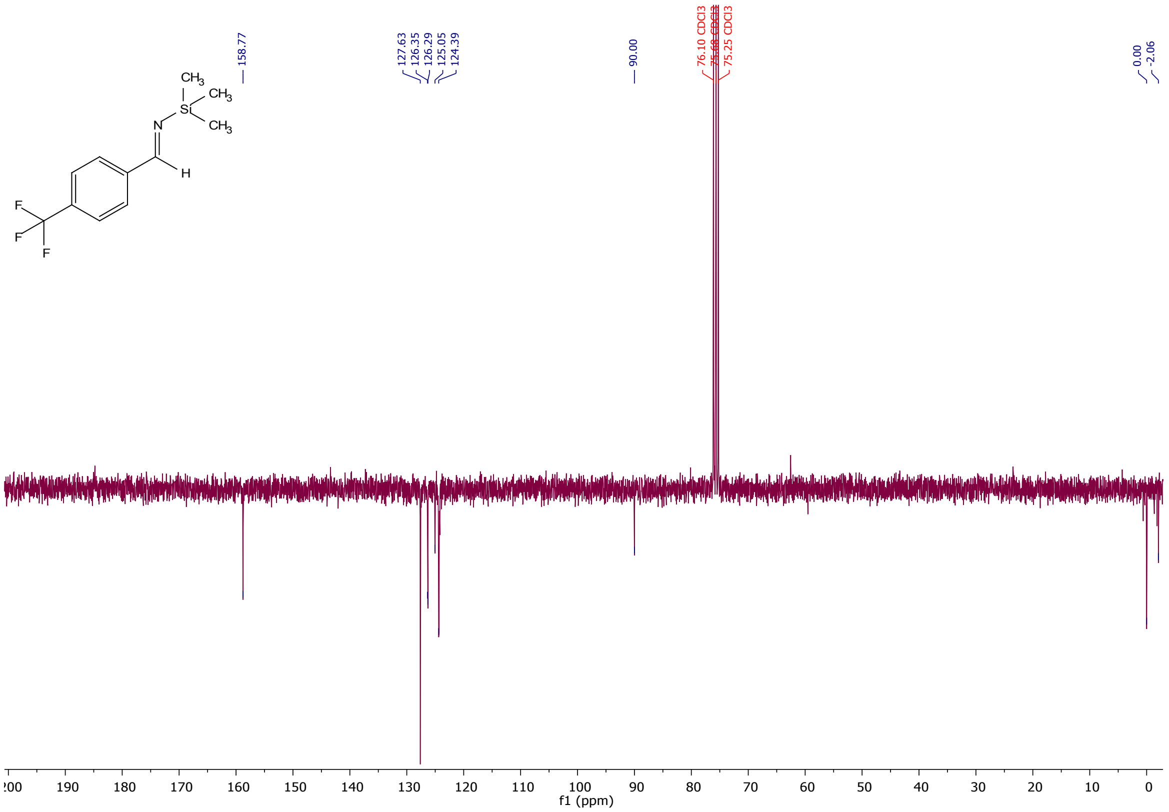

^{13}C {^{1}H} NMR Spectra of **5g,75 MHz (CDCl$_3$)**

5h ^{1}H

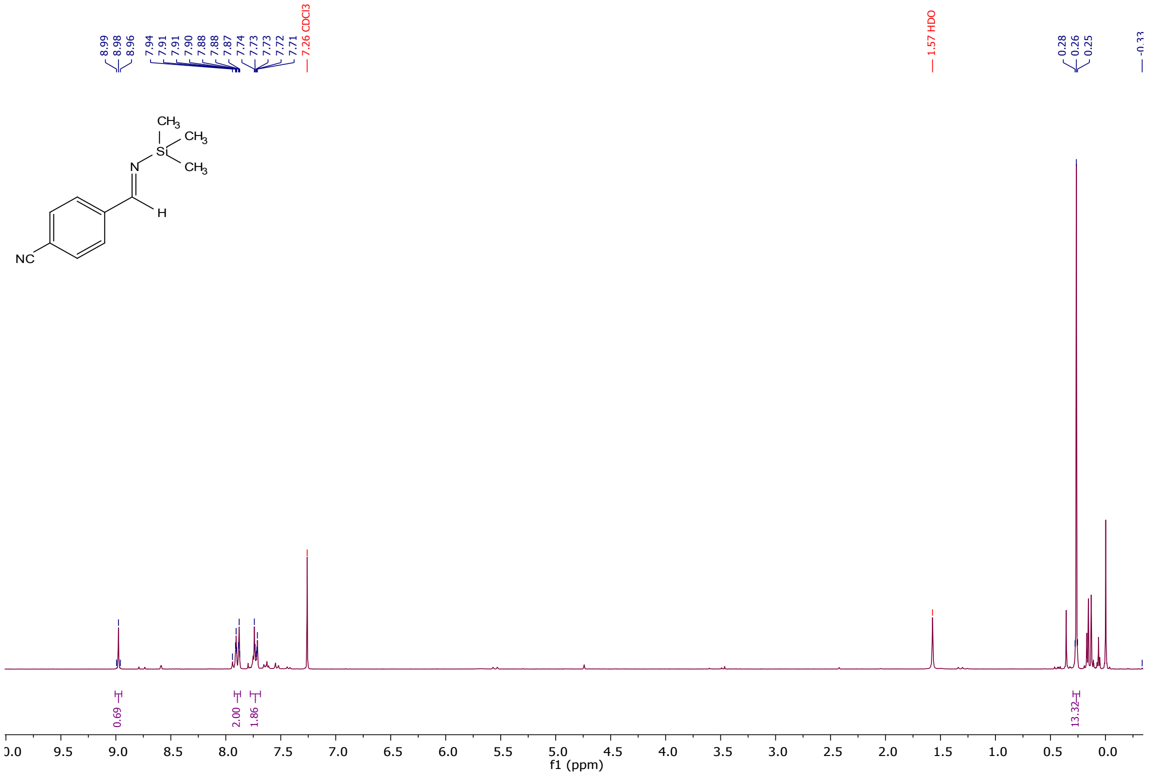

^{1}H NMR Spectra of **5h, 300 MHz (CDCl$_3$)**

5h ^{13}C {^{1}H}

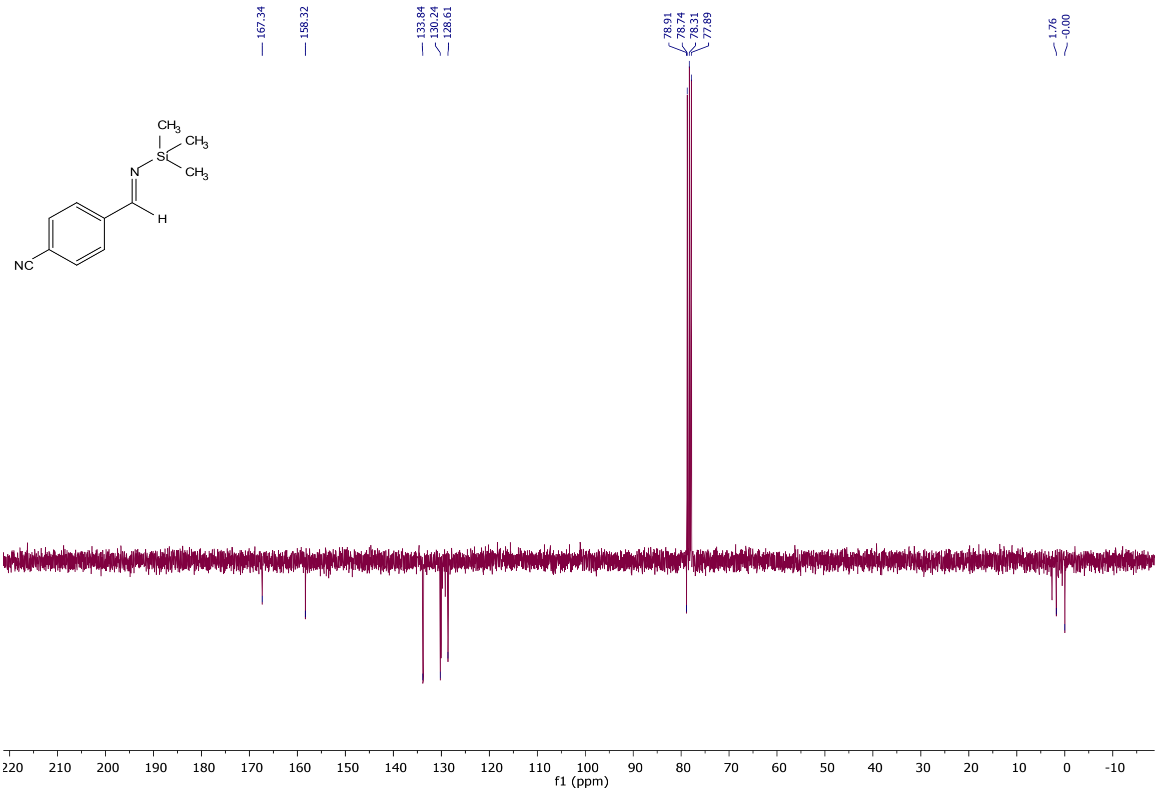

$^{13}C\{^{1}H\}$ NMR Spectra of **5h,75 MHz (CDCl$_3$)**

5i ^{1}H

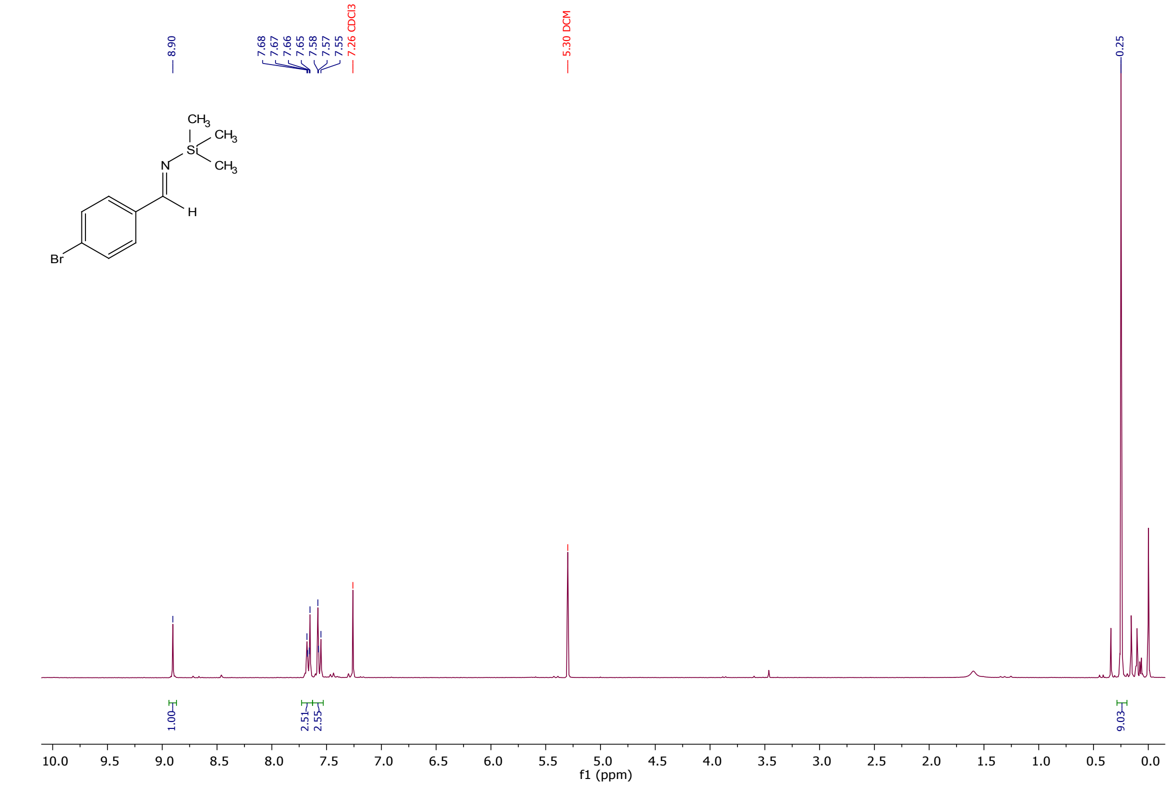

^{1}H NMR Spectra of **5i, 300 MHz (CDCl$_3$)**

5i $^{13}C\{^{1}H\}$

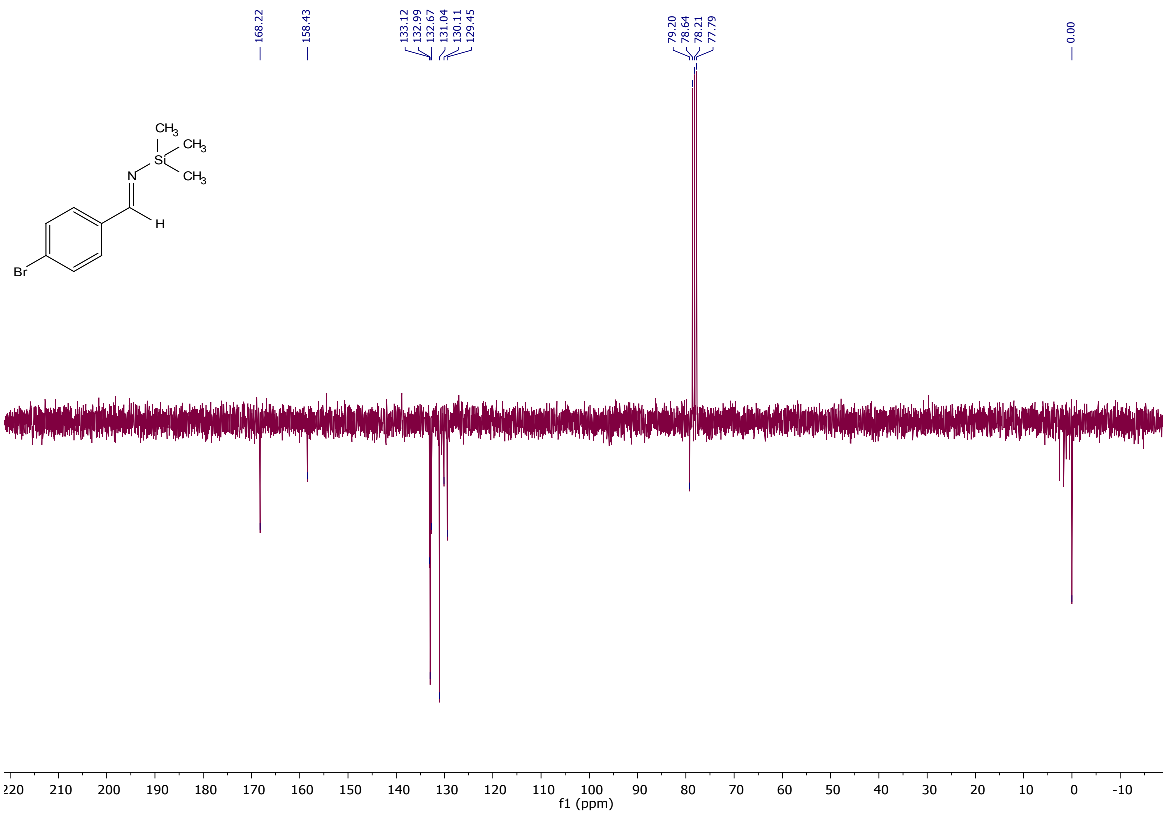

^{13}C {^{1}H} NMR Spectra of **5i,75 MHz (CDCl$_3$)**

5j ^{1}H

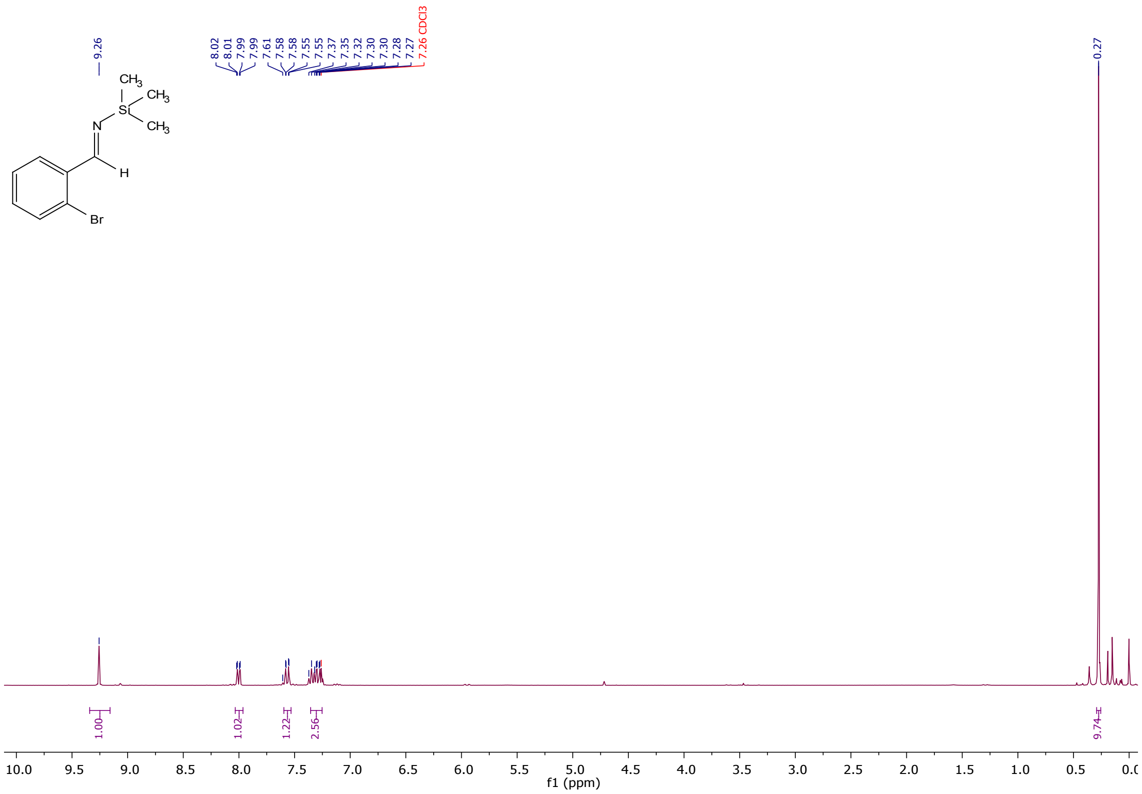

^{1}H NMR Spectra of **5j, 300 MHz (CDCl$_3$)**

5j $^{13}C\{^1H\}$

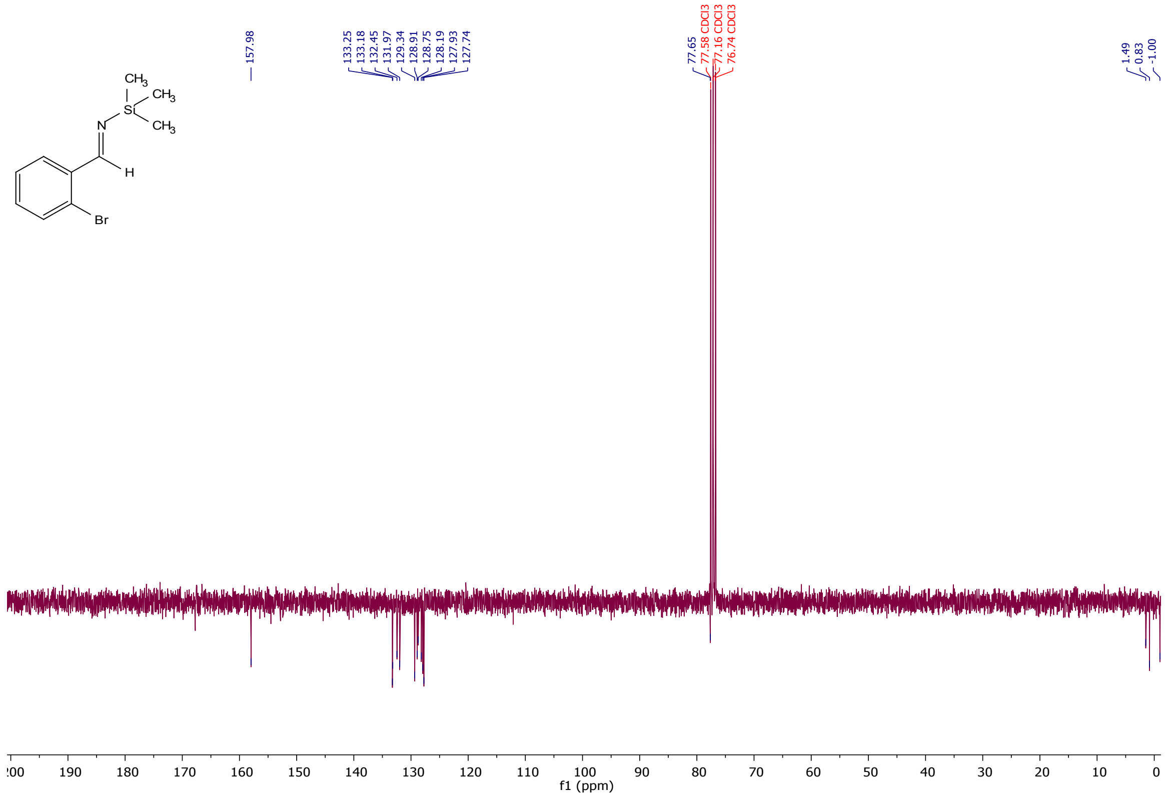

$^{13}C\{^1H\}$ NMR Spectra of **5j,75 MHz** **($CDCl_3$)**

5l ^1H

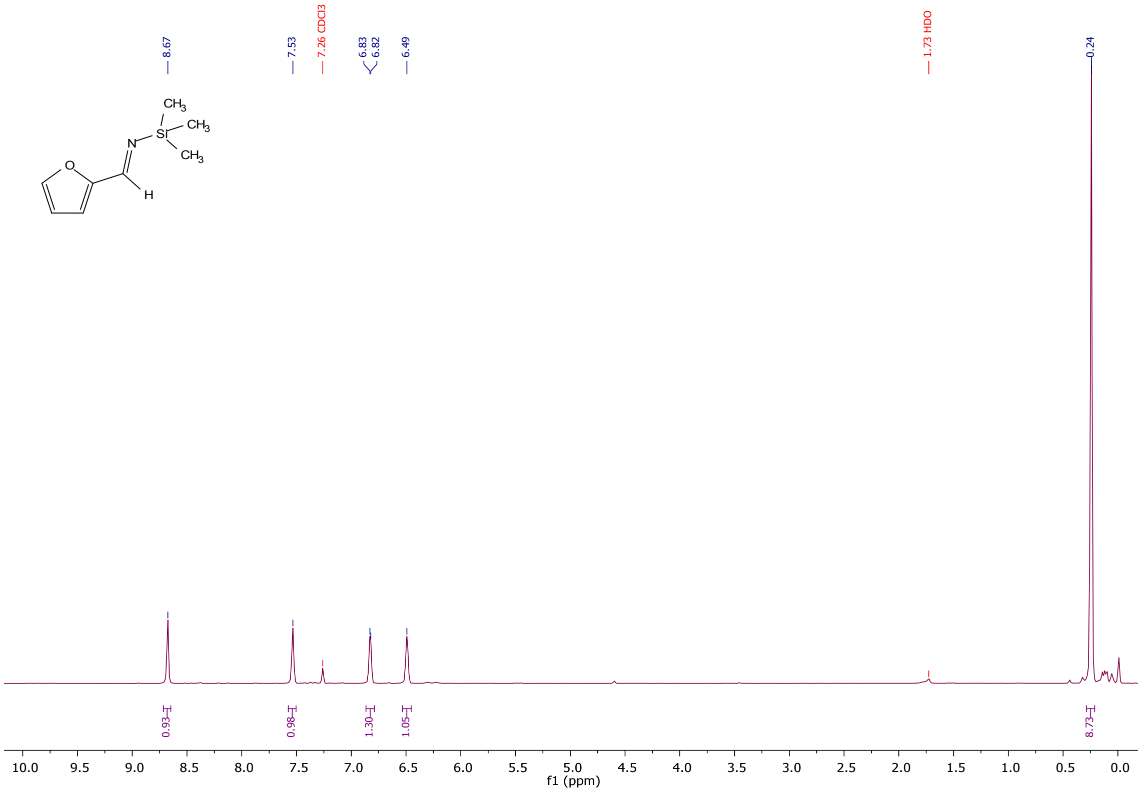

^{1}H NMR Spectra of **5l, 300 MHz (CDCl$_3$)**

5l ^{13}C {^{1}H}

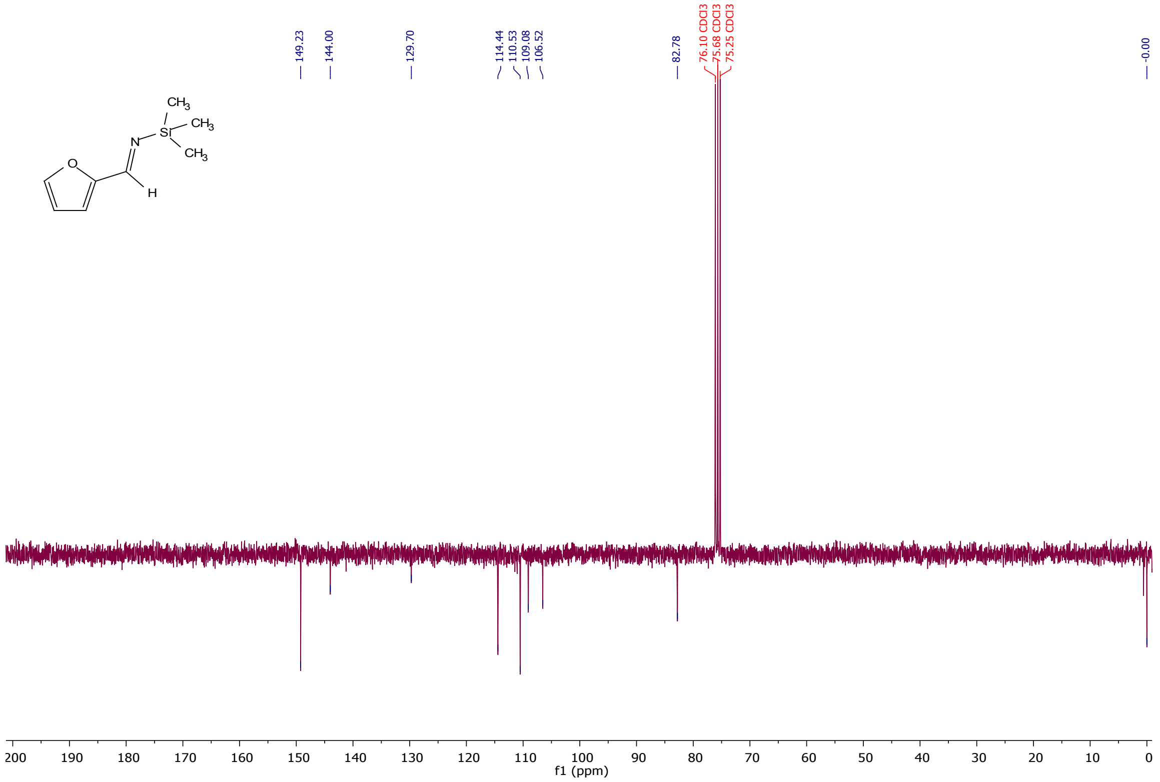

^{13}C {^{1}H} NMR Spectra of **5l,75 MHz (CDCl$_3$)**

5o ^{1}H

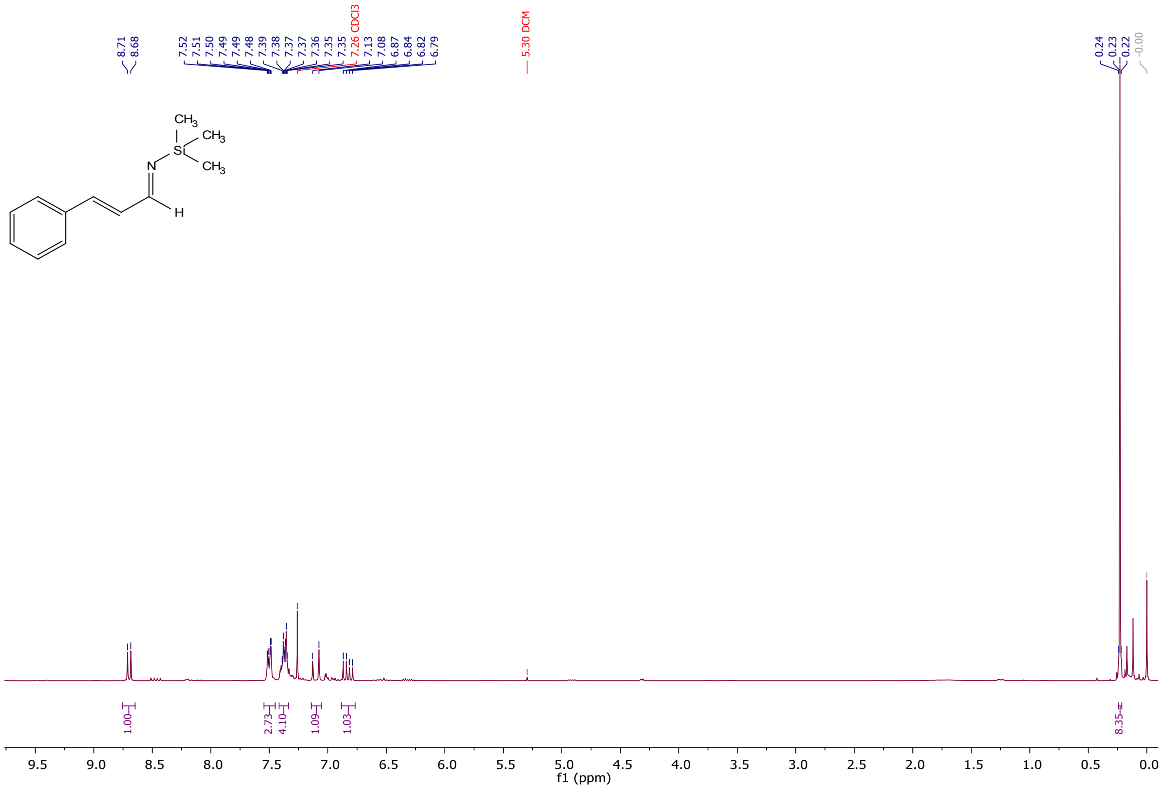

^{1}H NMR Spectra of **5o, 300 MHz (CDCl$_3$)**

5o ^{13}C {^{1}H}

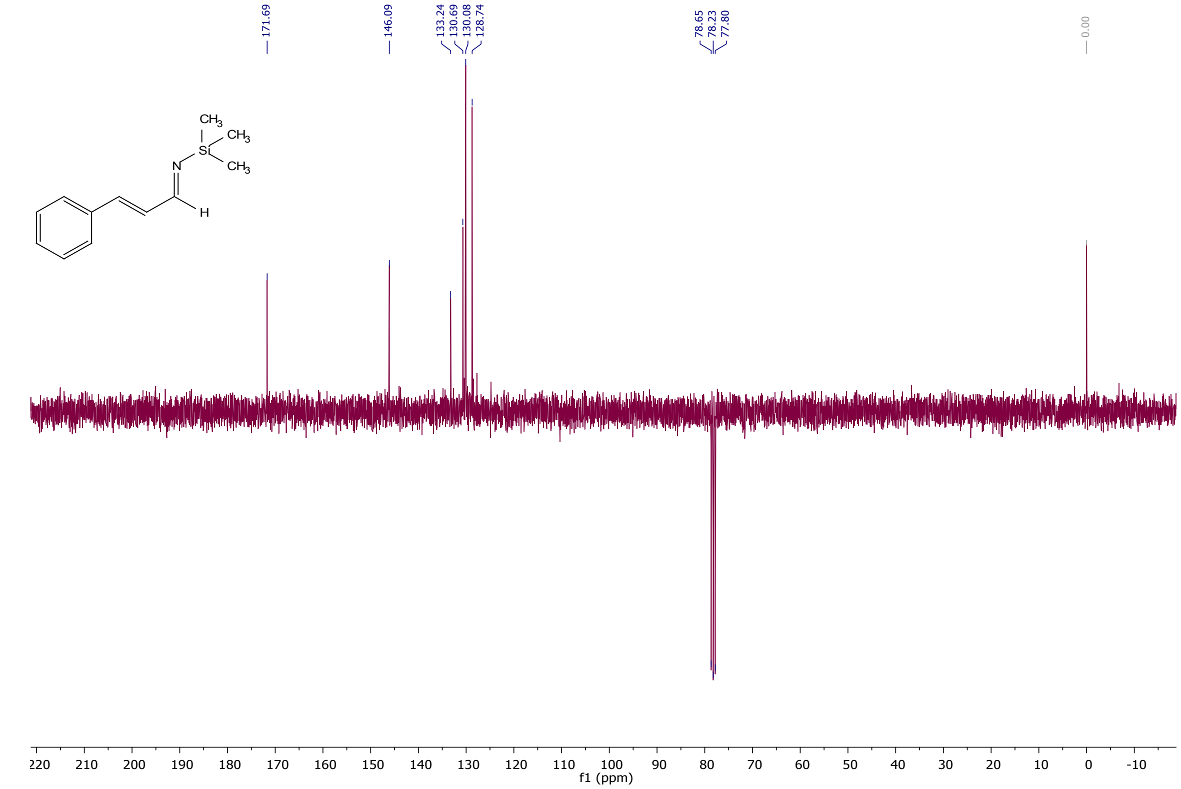

^{13}C {^{1}H} NMR Spectra of **5o,75 MHz (CDCl$_3$)**

5p ^{1}H

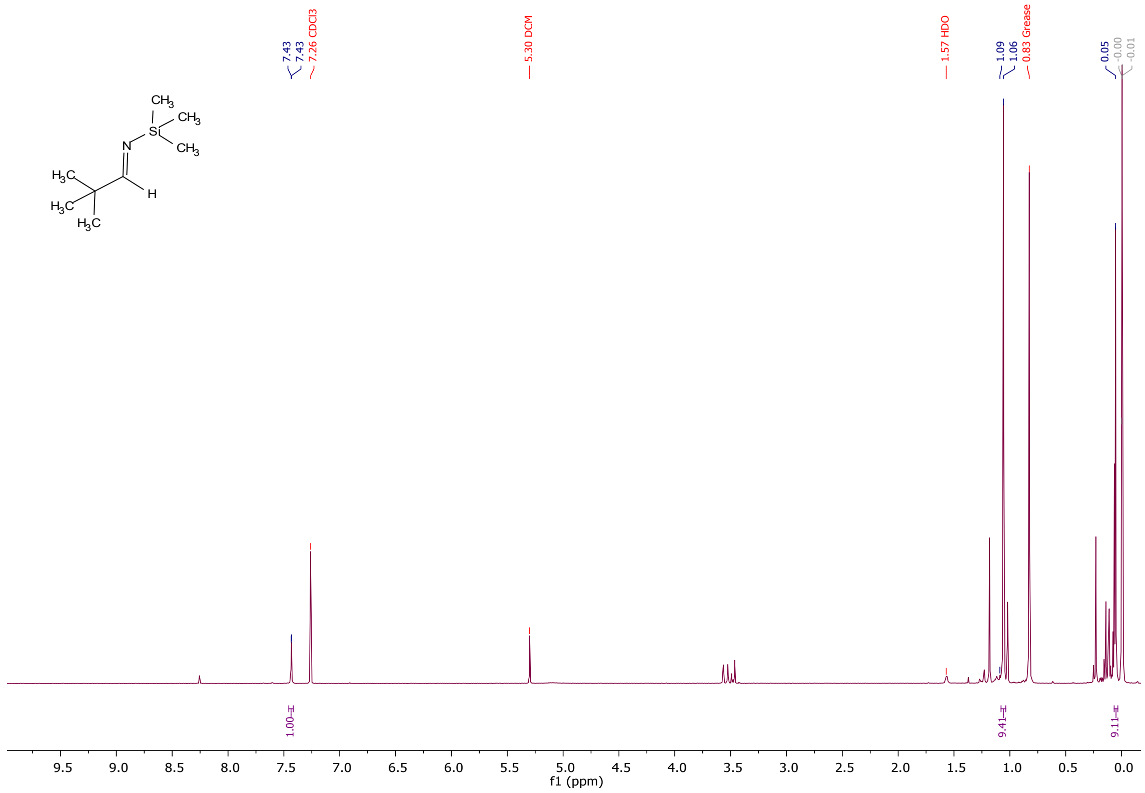

^{1}H NMR Spectra of **5p, 300 MHz (CDCl$_3$)**

5p ^{13}C {^{1}H}

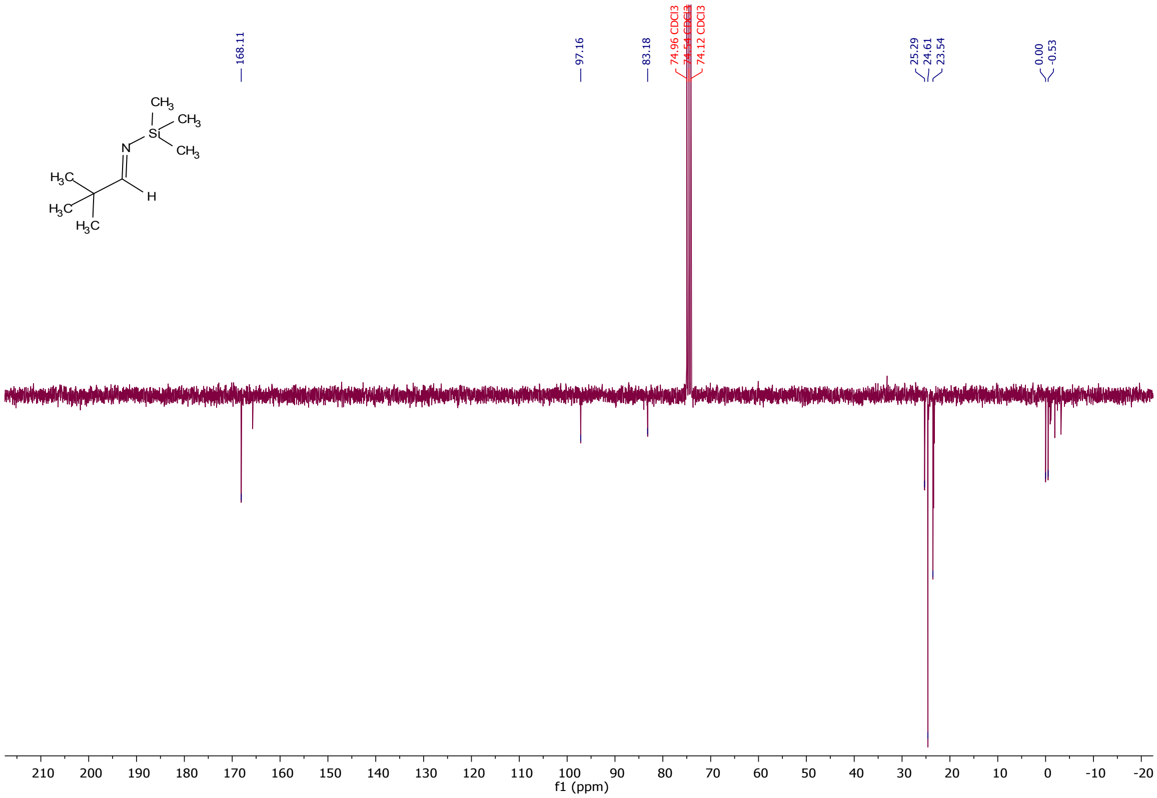

^{13}C {^{1}H} NMR Spectra of **5p,75 MHz (CDCl$_3$)**

7. Spectral Data Thiazolines

7a ¹H

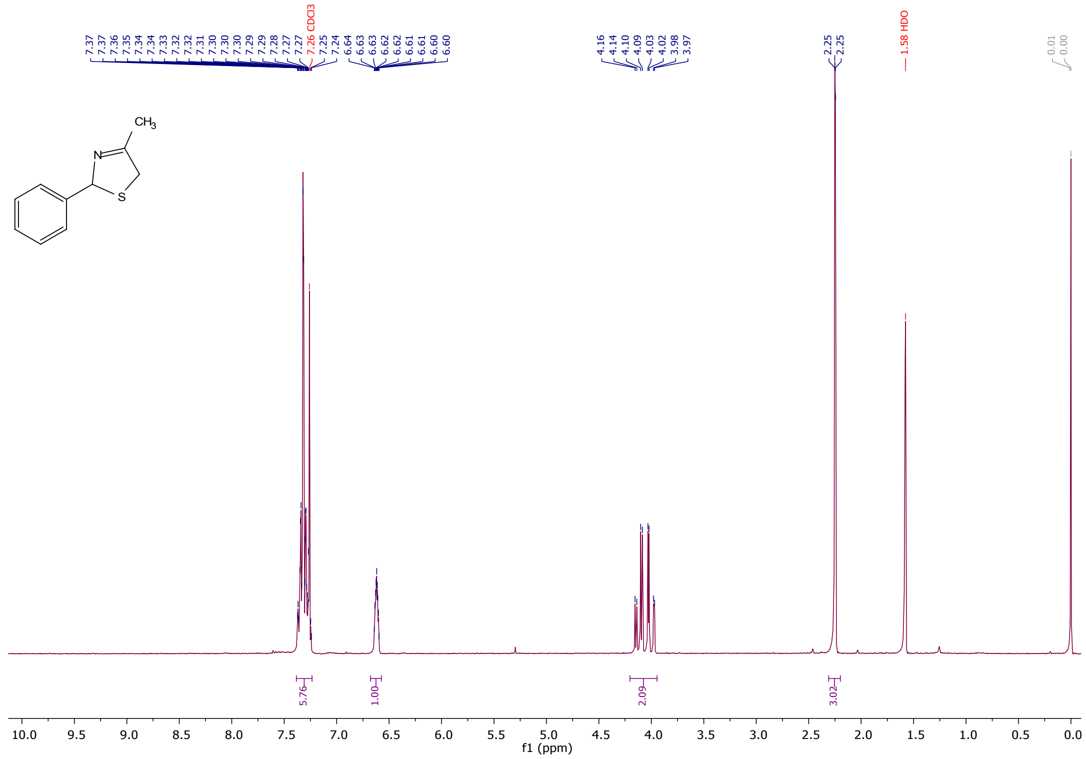

^{1}H NMR Spectra of **7a, 300 MHz (CDCl$_3$)**

7a ^{13}C {^{1}H}

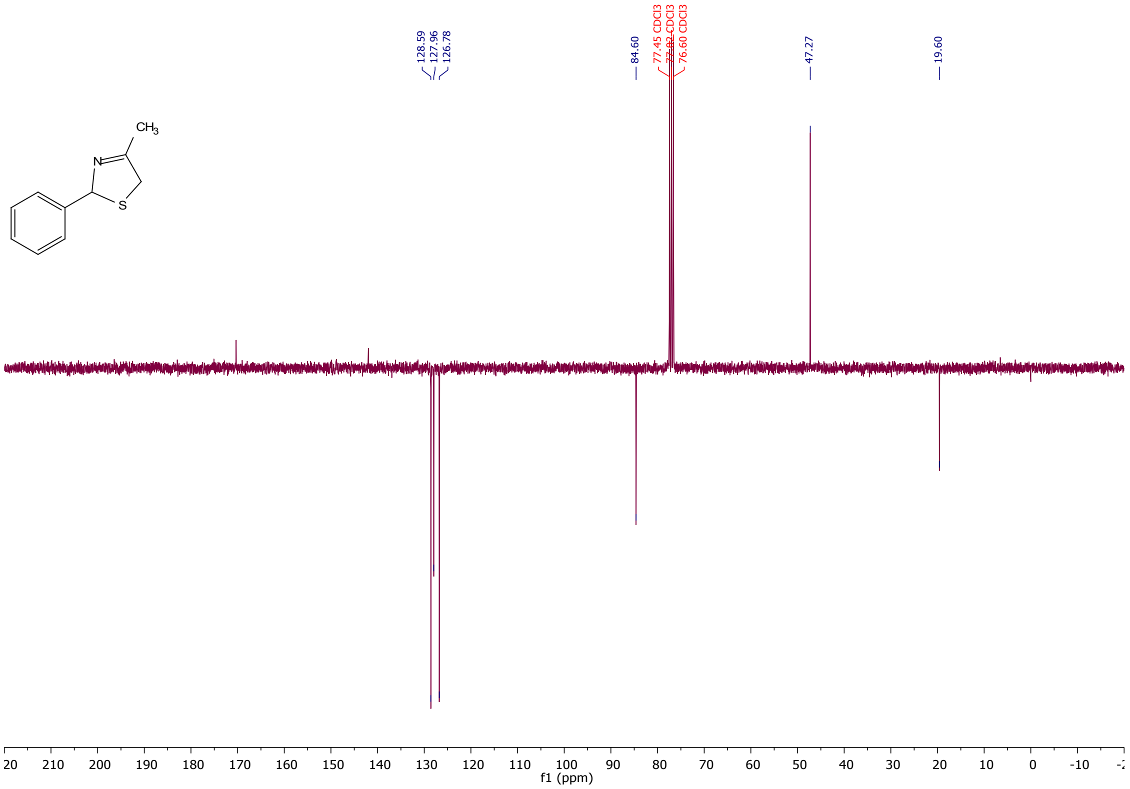

^{13}C {^{1}H} NMR Spectra of **7a,75 MHz (CDCl$_3$)**

7b ^{1}H

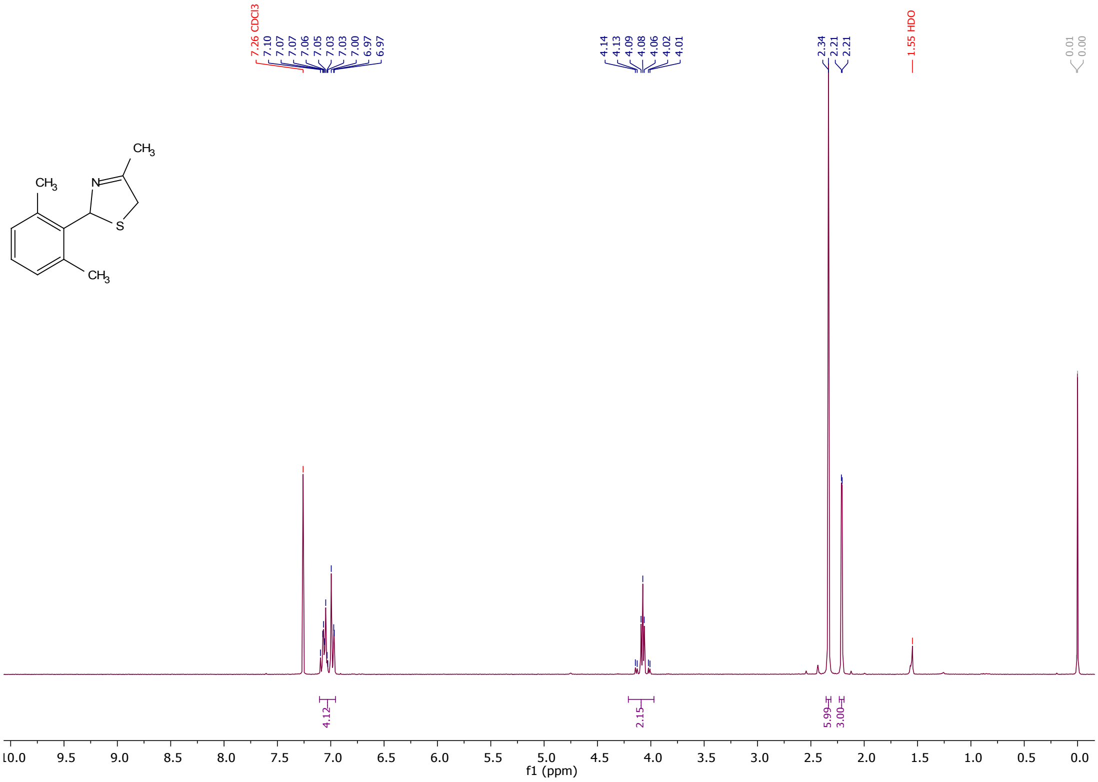

^{1}H NMR Spectra of **7b, 300 MHz (CDCl$_3$)**

7b ^{13}C {^{1}H}

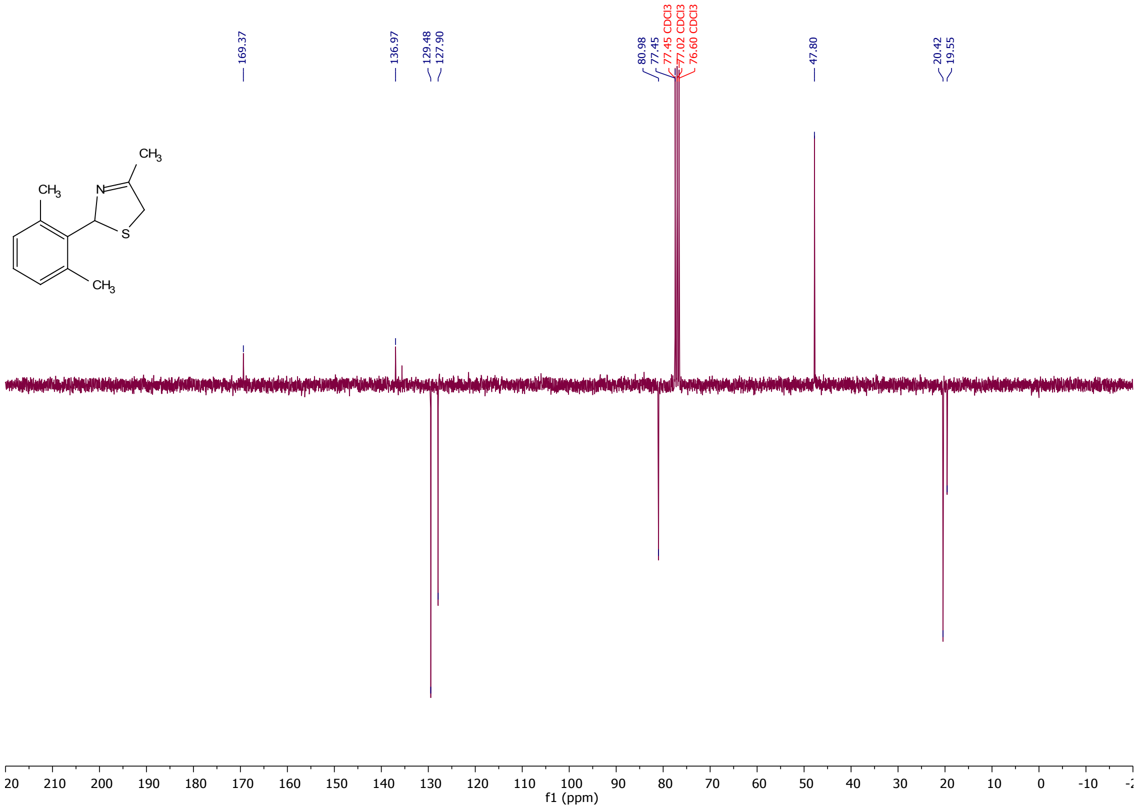

$^{13}C\{^1H\}$ NMR Spectra of **7b,75 MHz (CDCl$_3$)**

7c ^1H

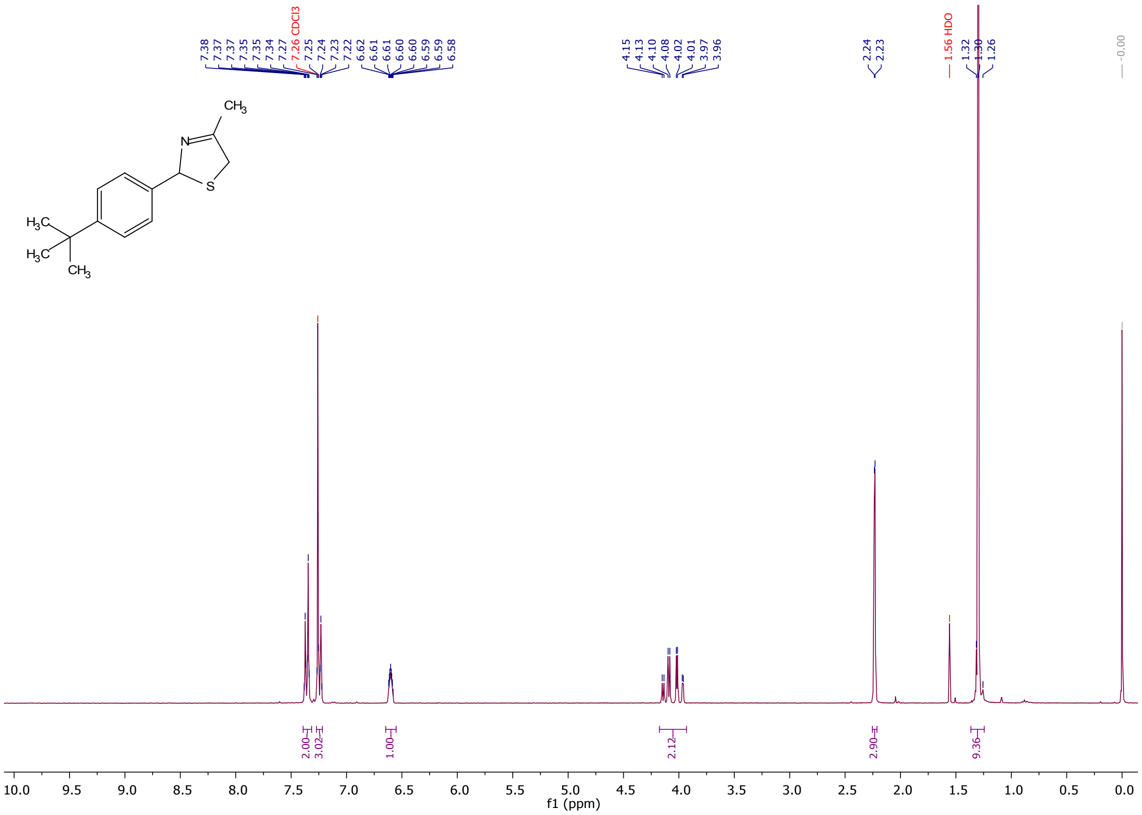

^1H NMR Spectra of **7c, 300 MHz (CDCl$_3$)**

7c ^{13}C {^{1}H}

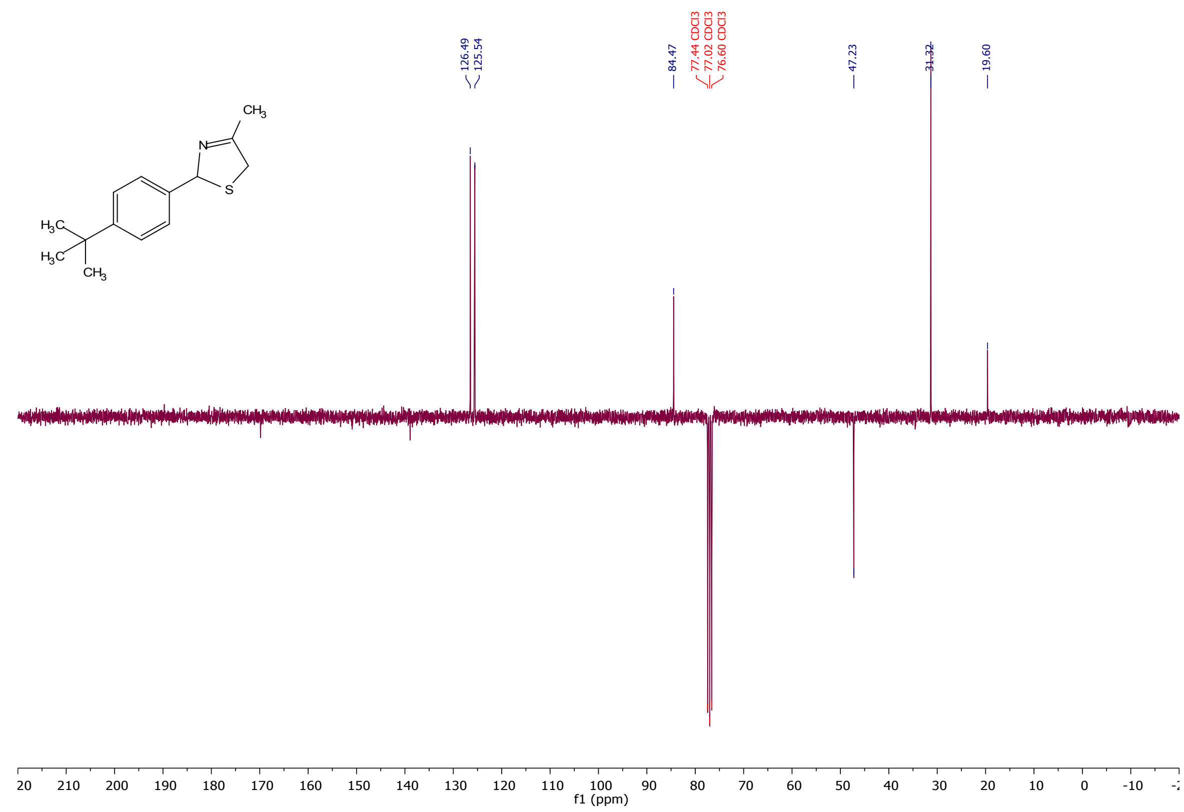

^{13}C {^{1}H} NMR Spectra of **7c,75 MHz (CDCl$_3$)**

7d ^{1}H

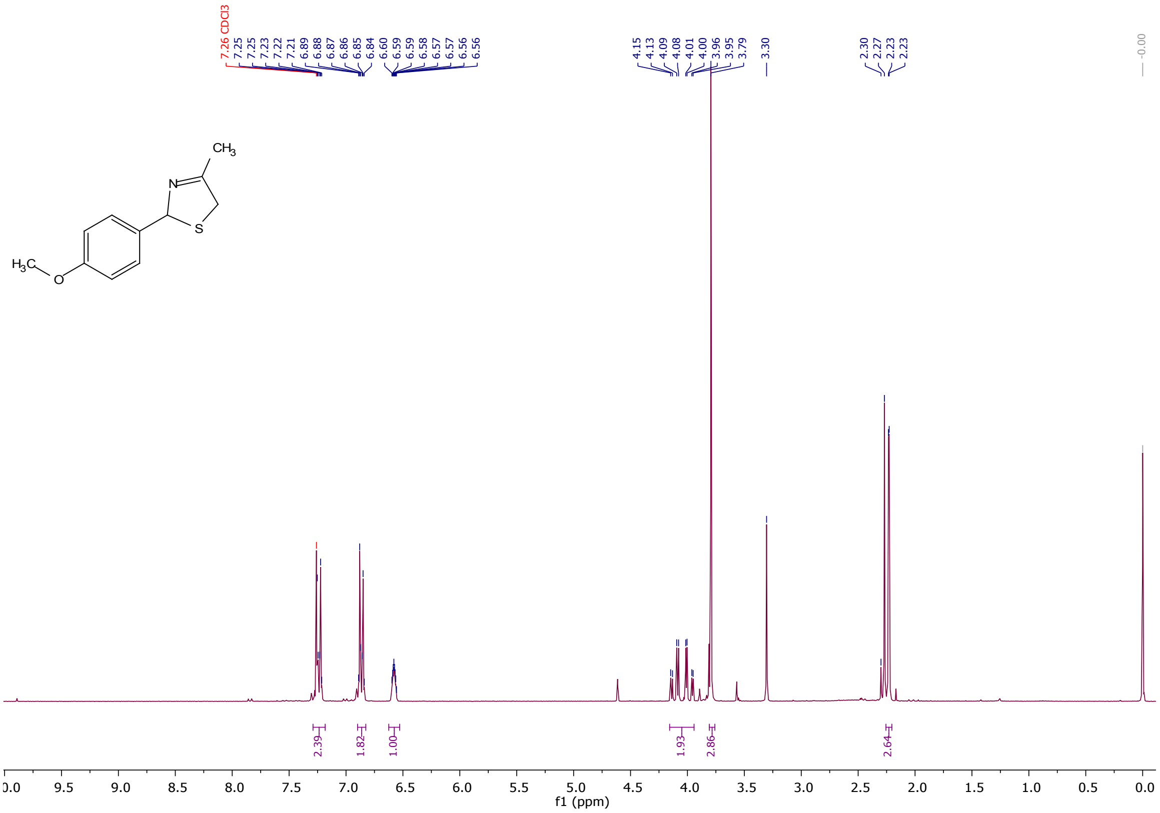

^{1}H NMR Spectra of **7d, 300 MHz (CDCl$_3$)**

7d ^{13}C {^{1}H}

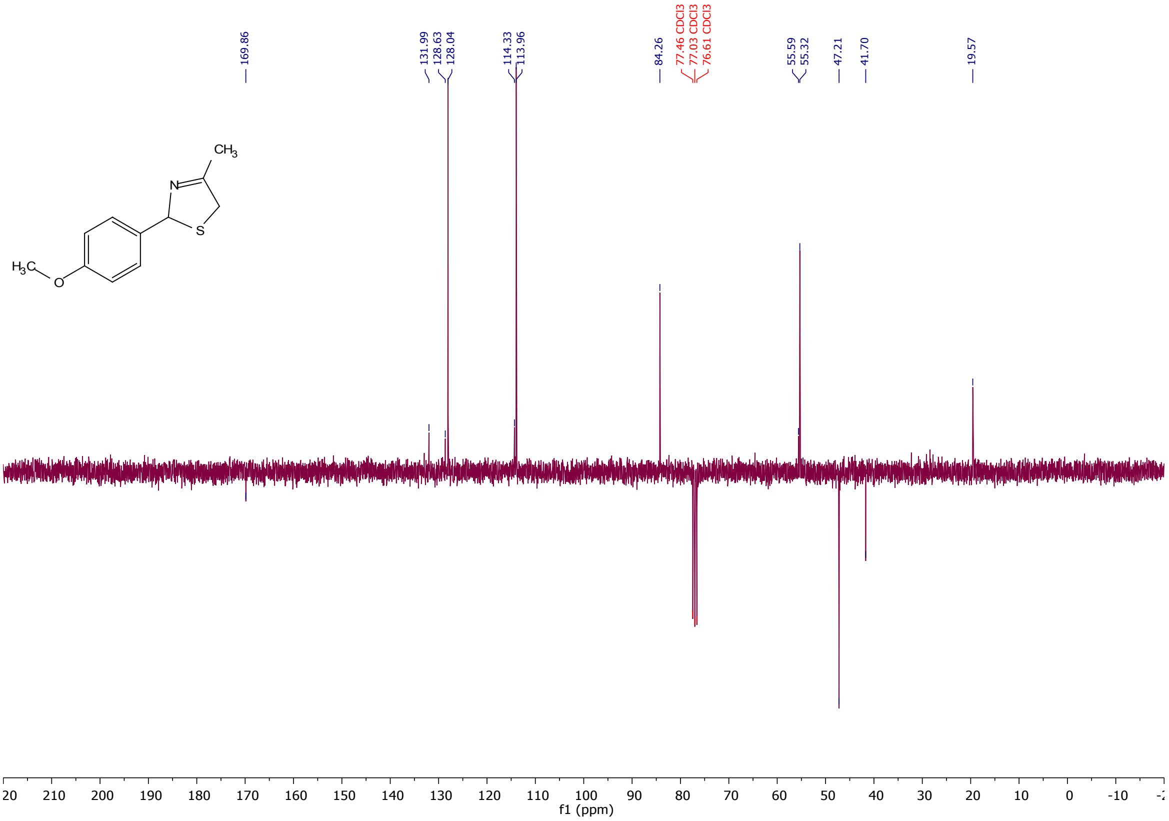

^{13}C {^{1}H} NMR Spectra of **7d,75 MHz (CDCl$_3$)**

7e ^{1}H

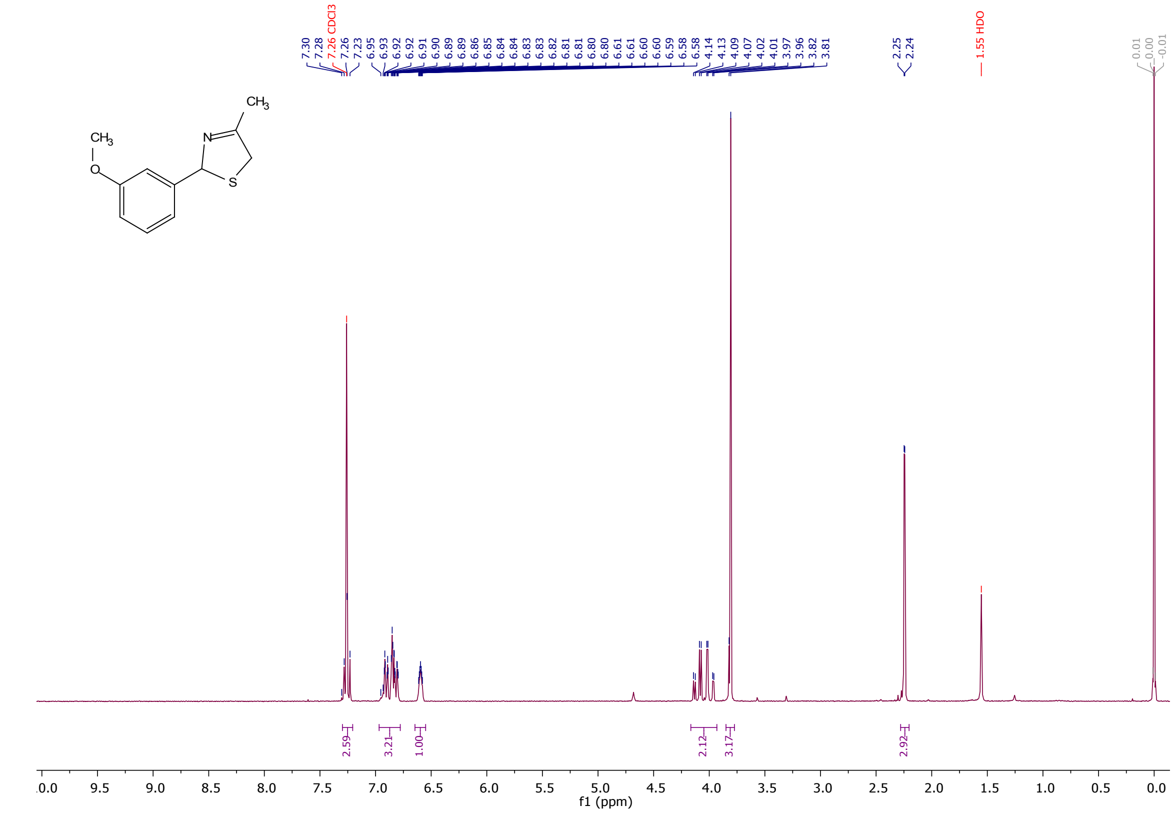

^{1}H NMR Spectra of **7e, 300 MHz (CDCl$_3$)**

7e ^{13}C {^{1}H}

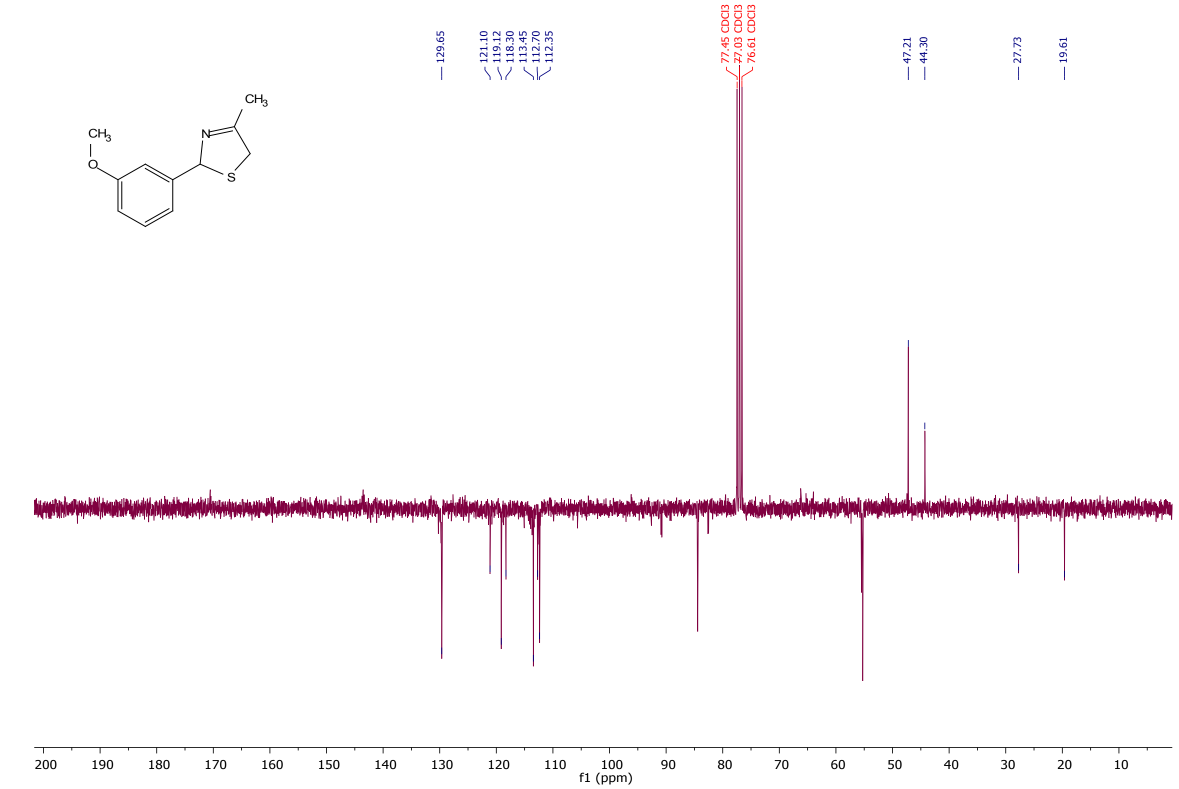

^{13}C {^{1}H} NMR Spectra of **7e,75 MHz (CDCl$_3$)**

7f ¹H

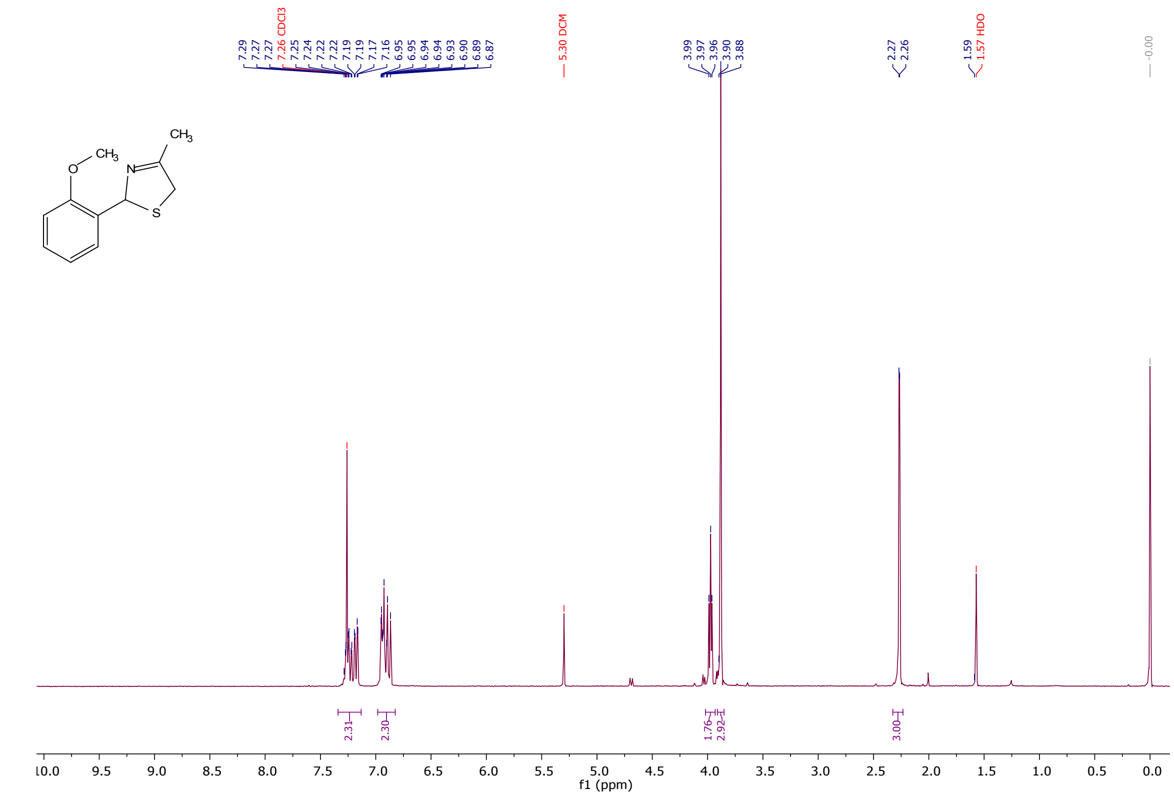

^{1}H NMR Spectra of **7f, 300 MHz (CDCl$_3$)**

7f ^{13}C {^{1}H}

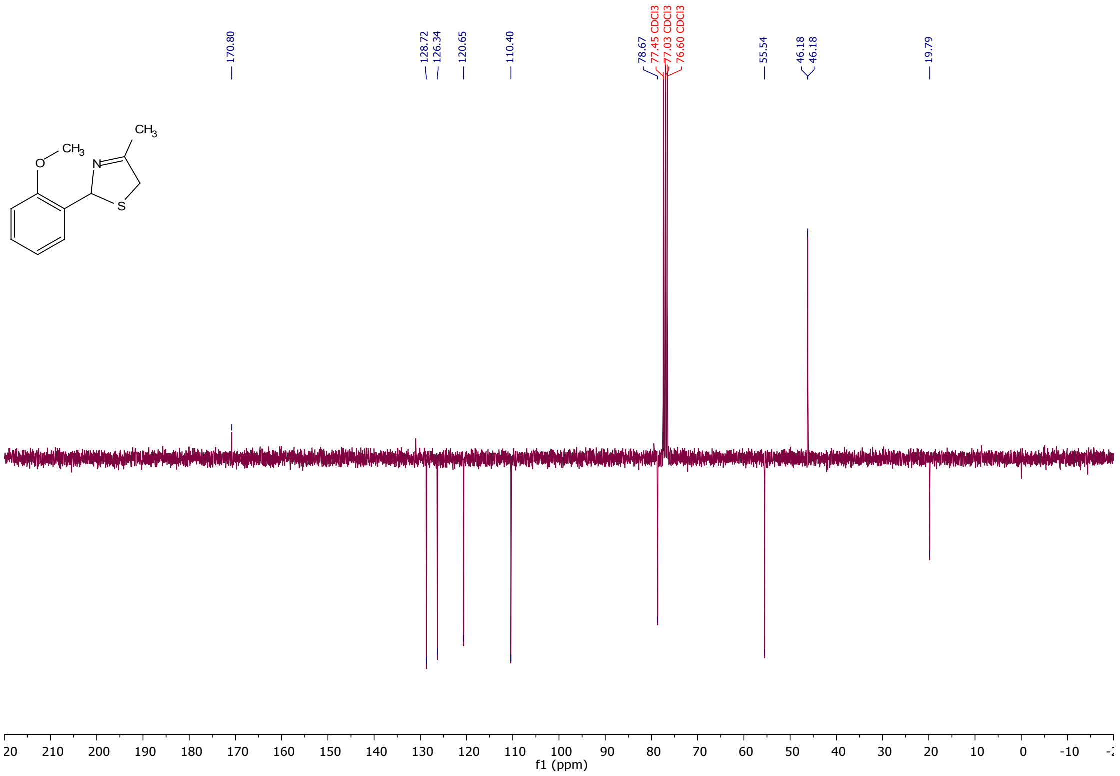

^{13}C {^{1}H} NMR Spectra of **7f,75 MHz (CDCl$_3$)**

7g ¹H

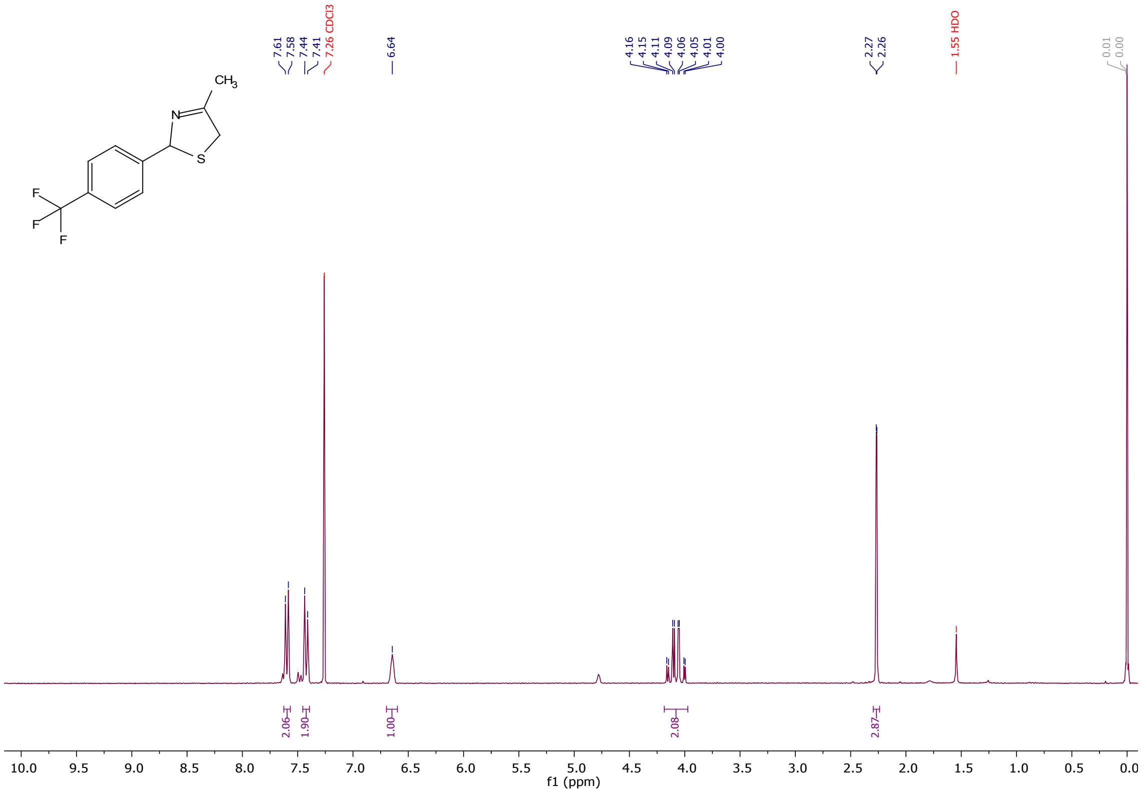

^{1}H NMR Spectra of **7g, 300 MHz (CDCl$_3$)**

7g ¹³C {¹H}

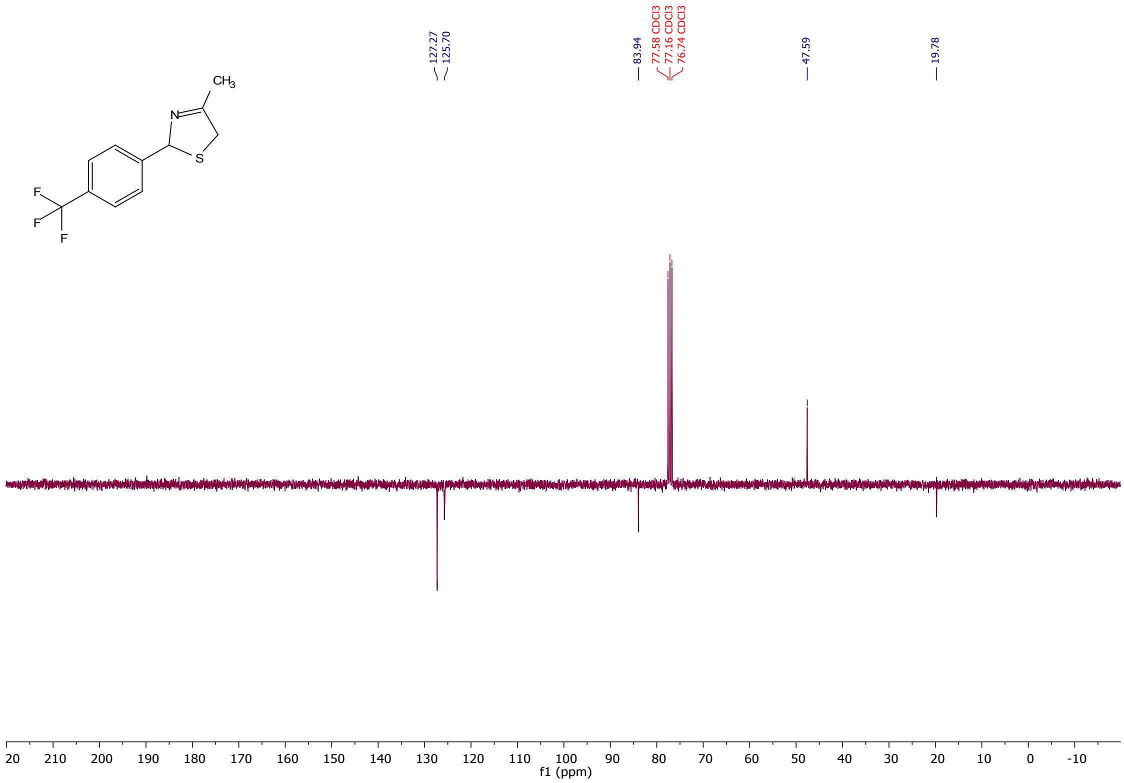

^{13}C {^{1}H} NMR Spectra of **7g,75 MHz (CDCl$_3$)**

7h ^{1}H

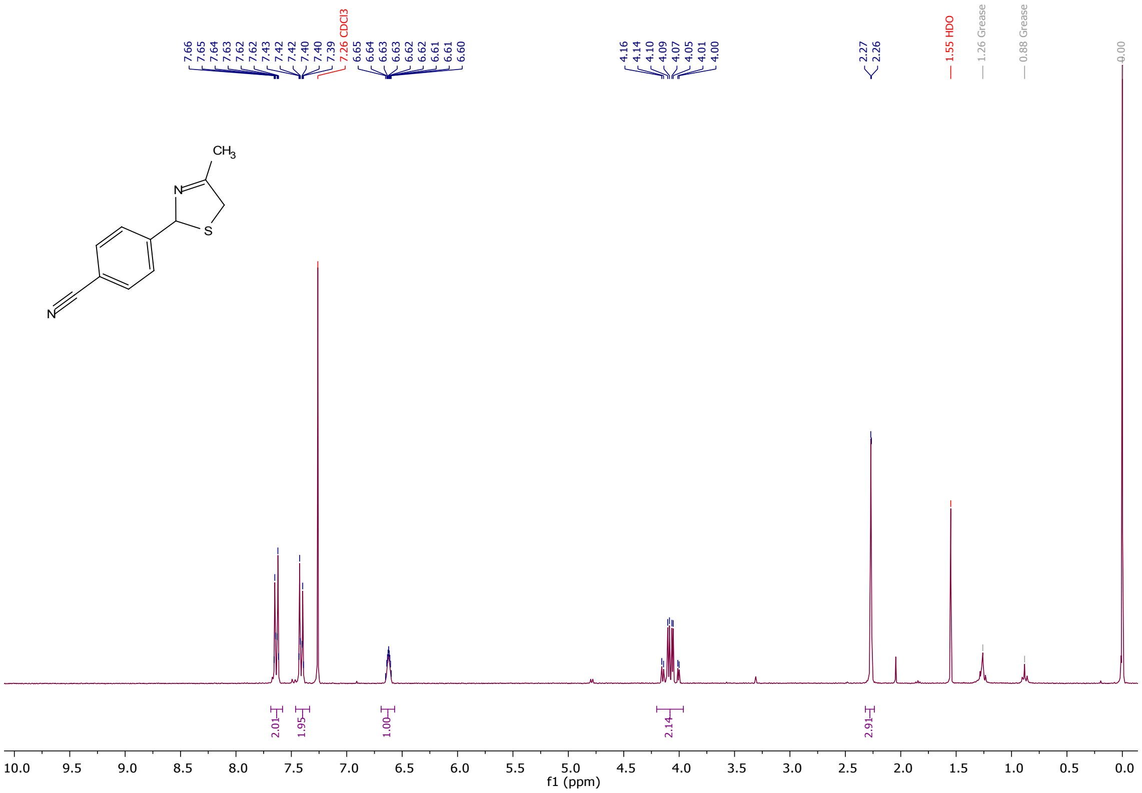

^{1}H NMR Spectra of **7h, 300 MHz (CDCl$_3$)**

7h ^{13}C {^{1}H}

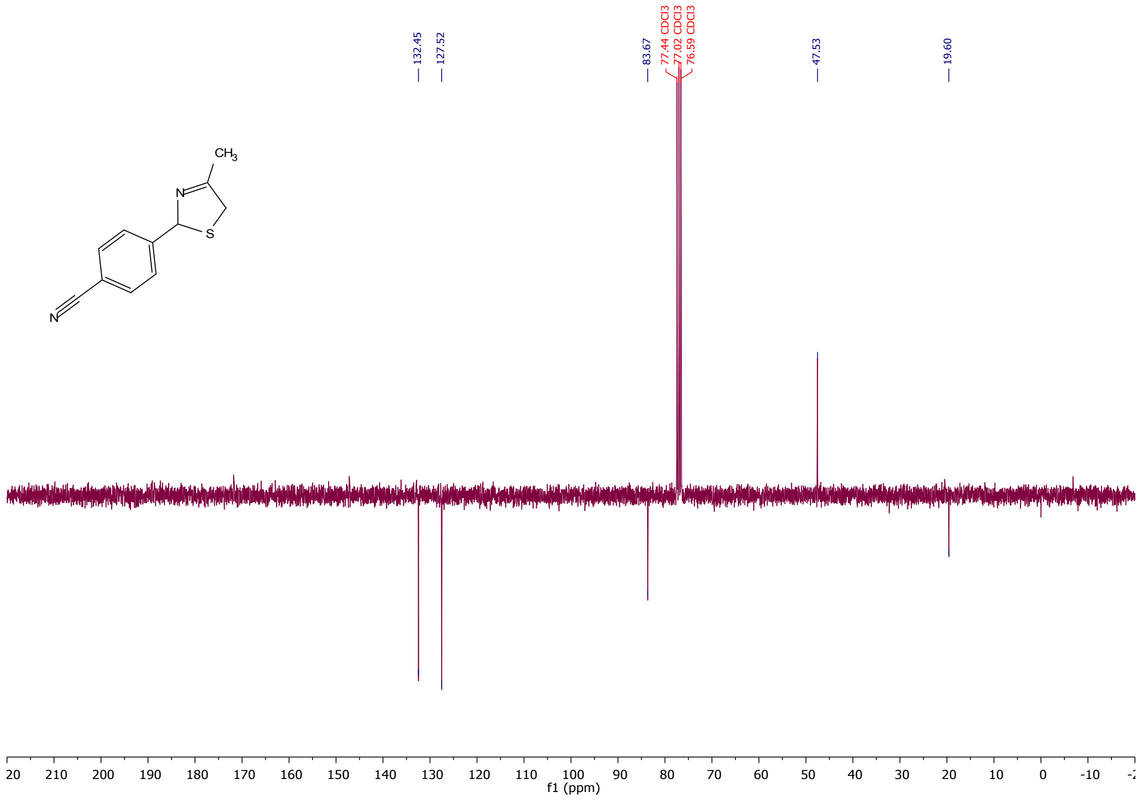

^{13}C {^{1}H} NMR Spectra of **7h,75 MHz (CDCl$_3$)**

7i ^{1}H

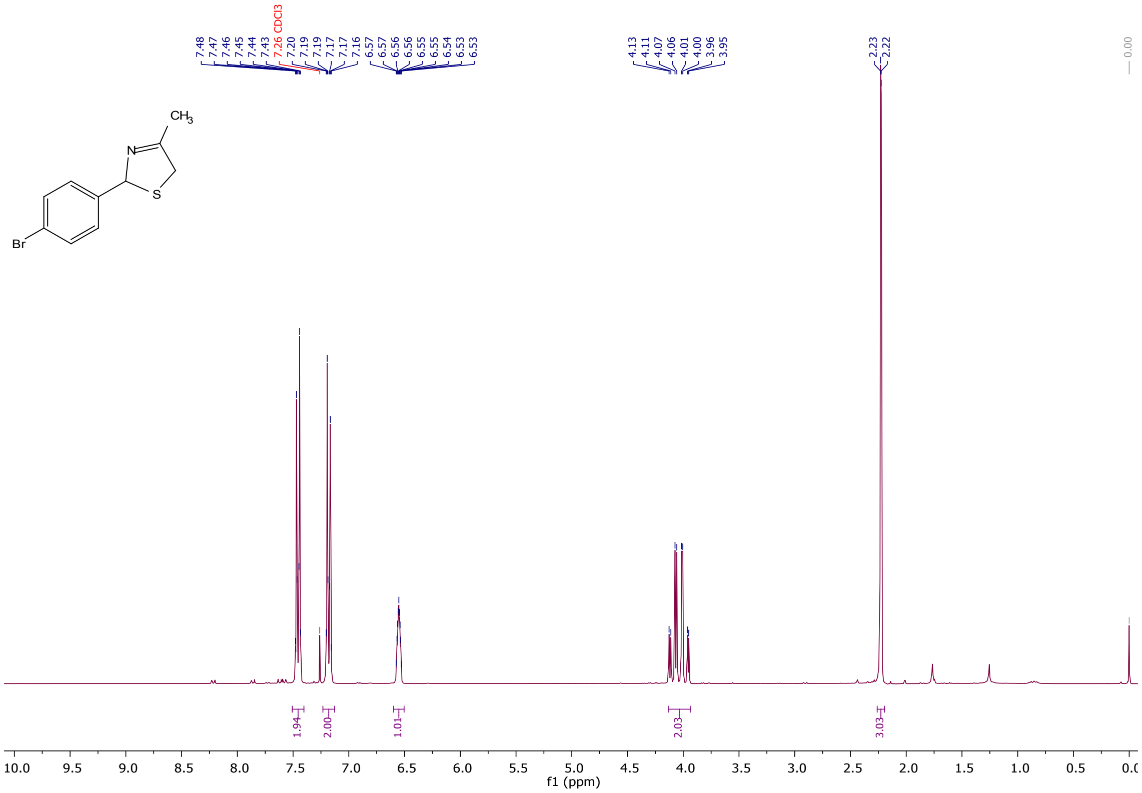

^{1}H NMR Spectra of **7i, 300 MHz (CDCl$_3$)**

7i ^{13}C {^{1}H}

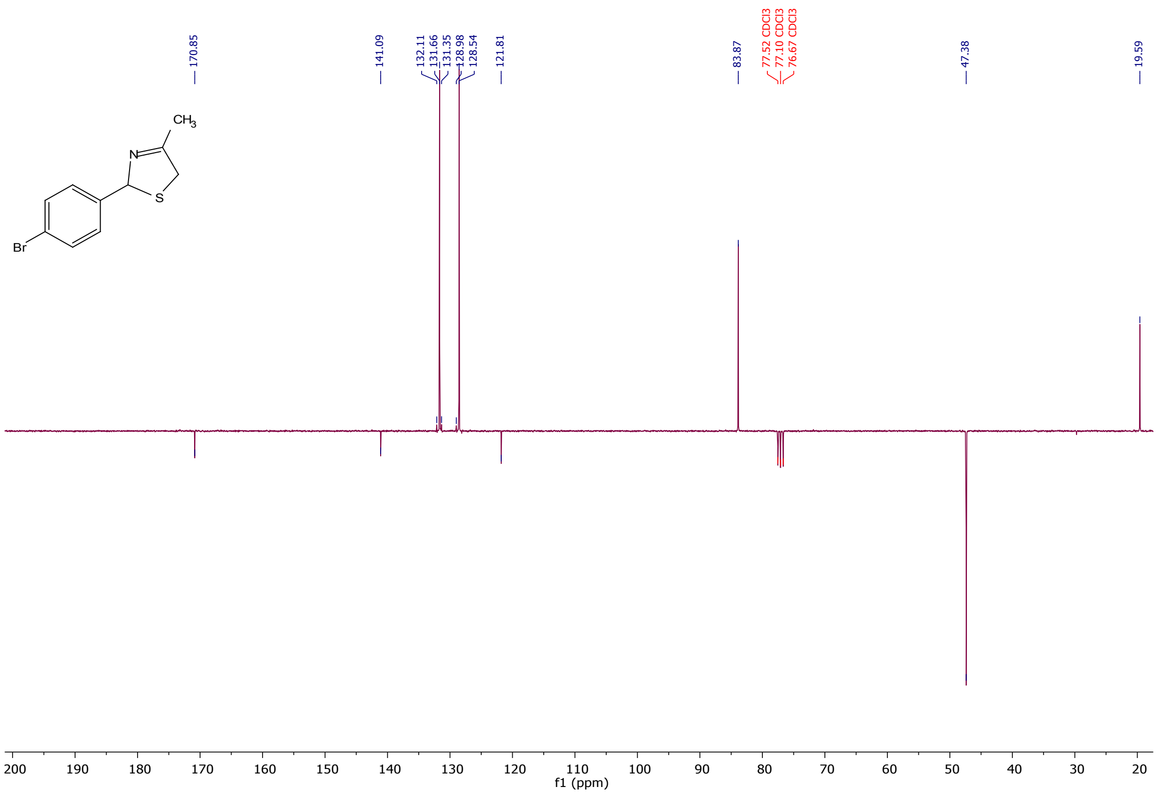

$^{13}C\{^1H\}$ NMR Spectra of **7i,75 MHz (CDCl$_3$)**

7j ¹H

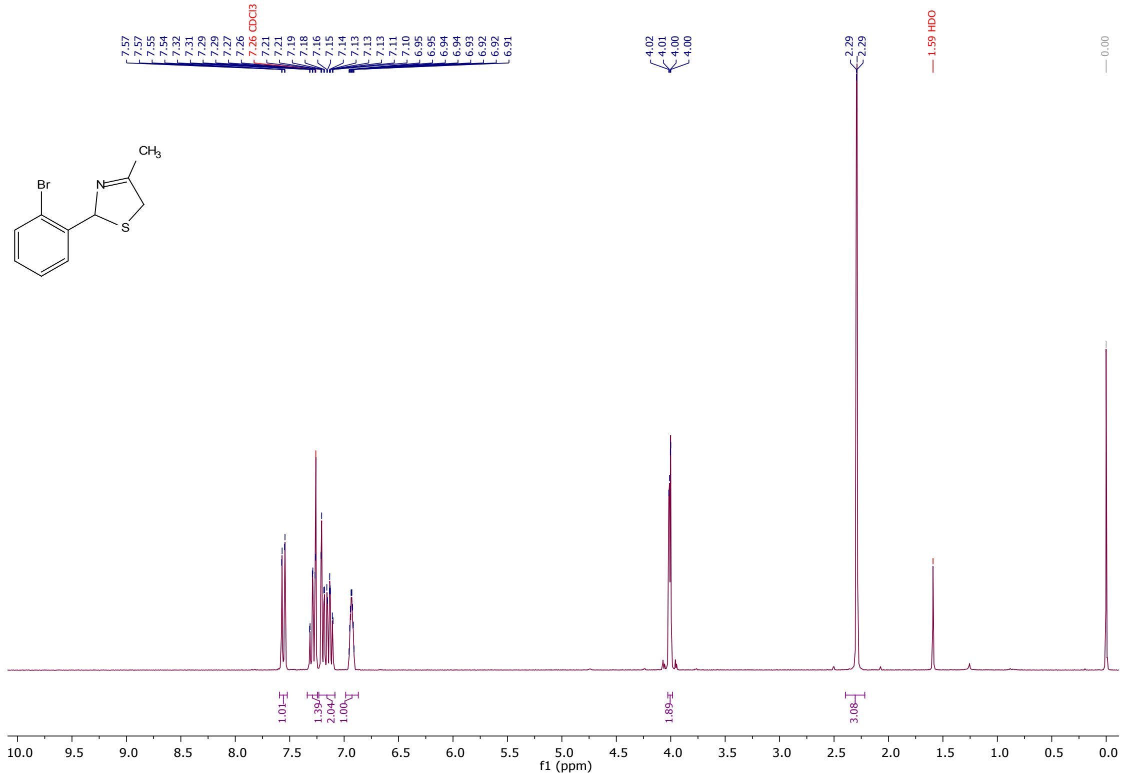

^1H NMR Spectra of **7j, 300 MHz (CDCl$_3$)**

7j ¹³C {¹H}

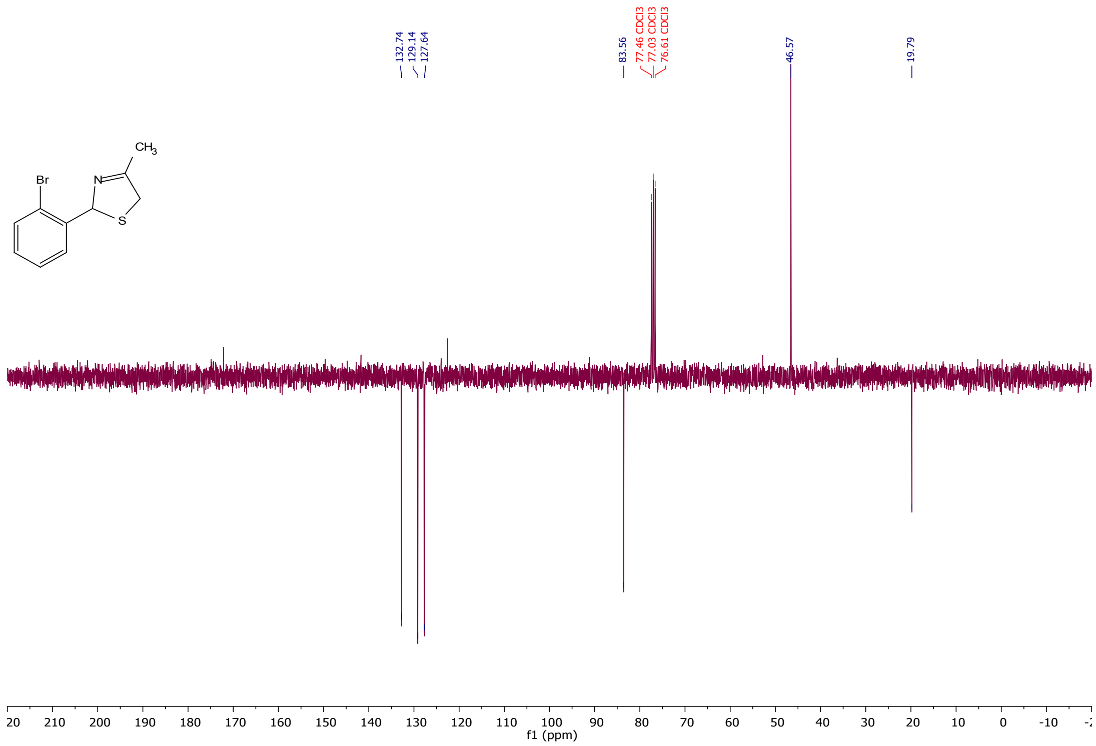

^{13}C {^1H} NMR Spectra of **7j,75 MHz (CDCl$_3$)**

7k ¹H

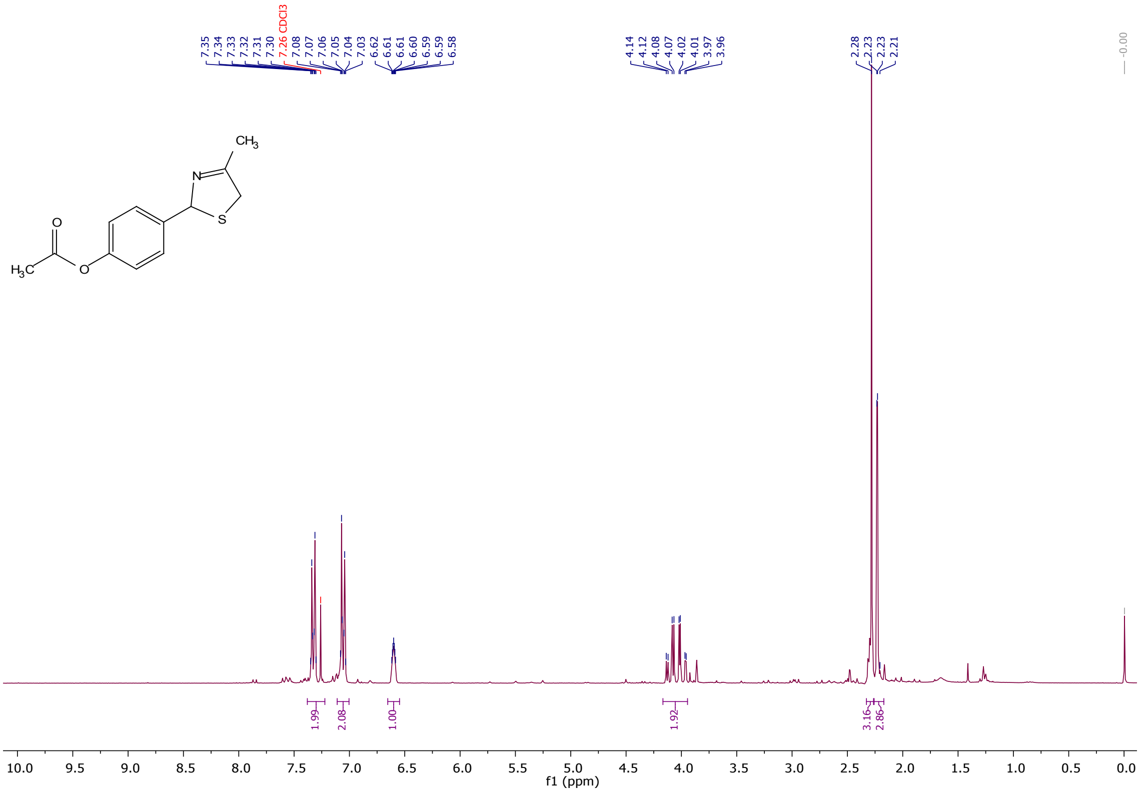

^1H NMR Spectra of **7k, 300 MHz (CDCl$_3$)**

7k $^{13}C\{^1H\}$

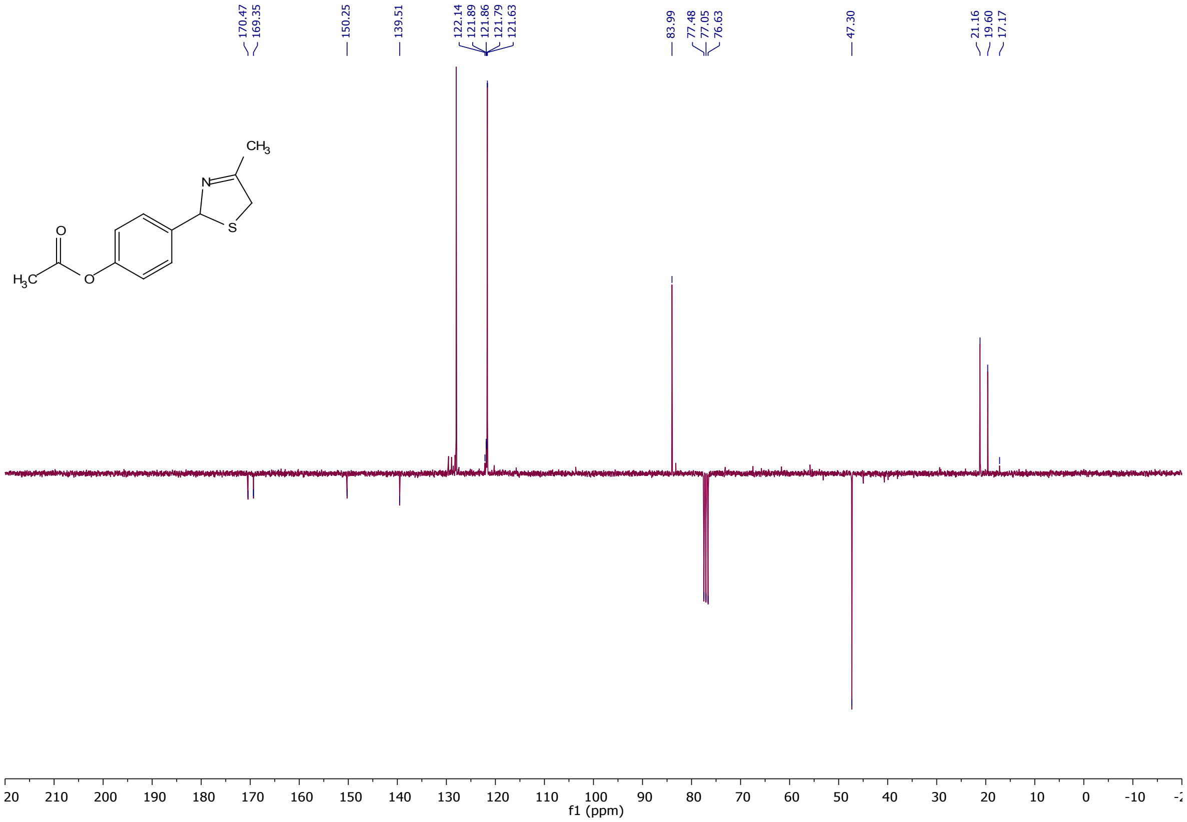

$^{13}C\{^1H\}$ NMR Spectra of **7k, 75 MHz ($CDCl_3$)**

7l ^1H

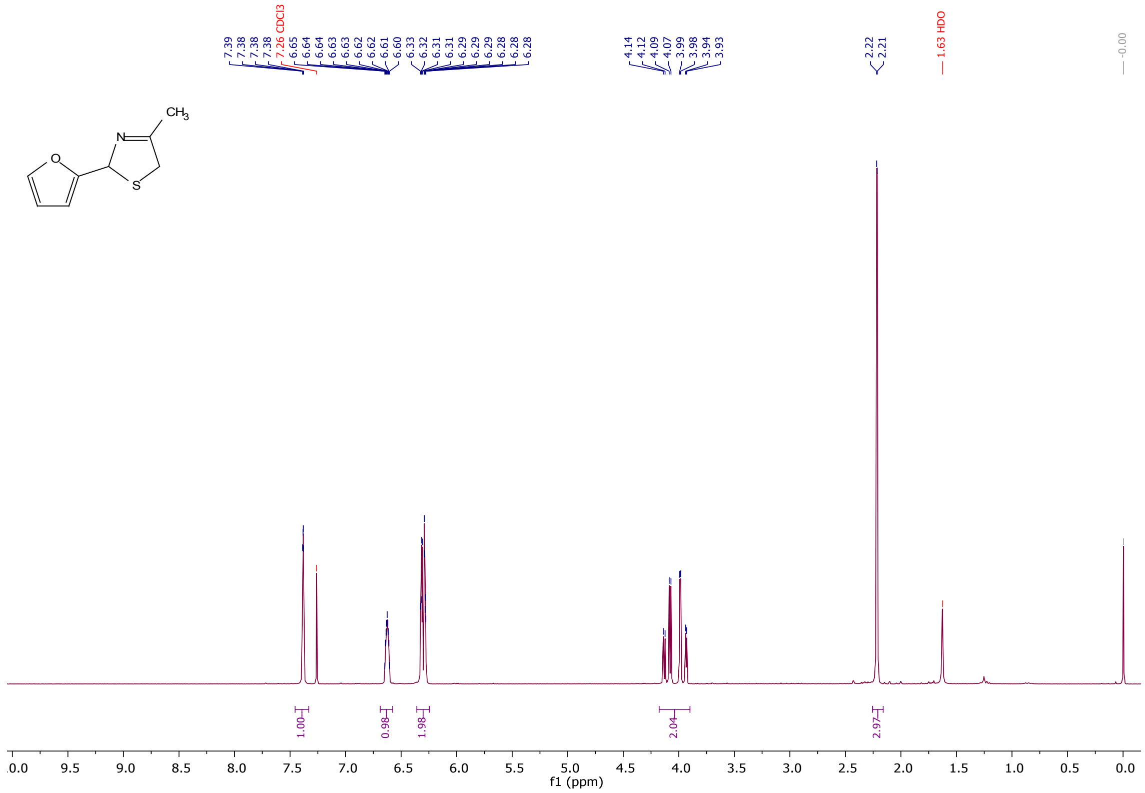

^{1}H NMR Spectra of **7l, 300 MHz (CDCl$_3$)**

7l ^{13}C {^{1}H}

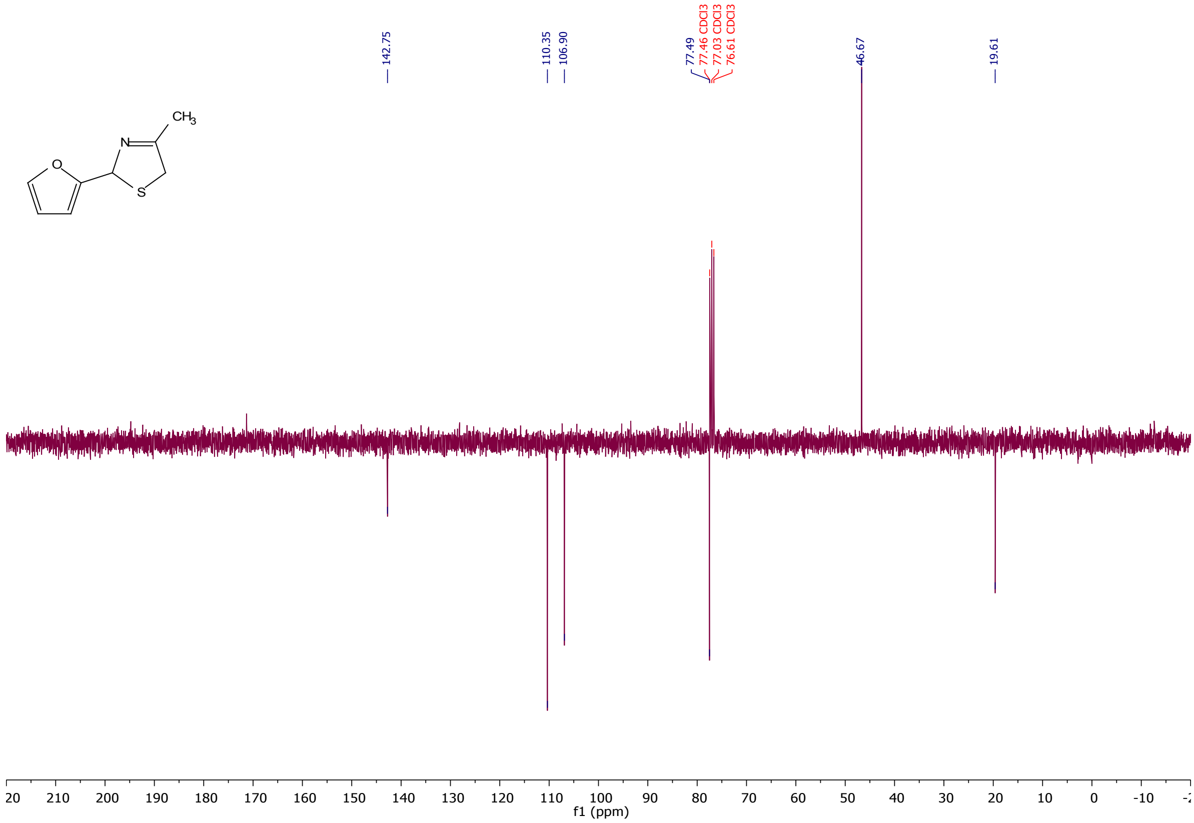

^{13}C {^{1}H} NMR Spectra of **7l,75 MHz (CDCl$_3$)**

7m ^{1}H

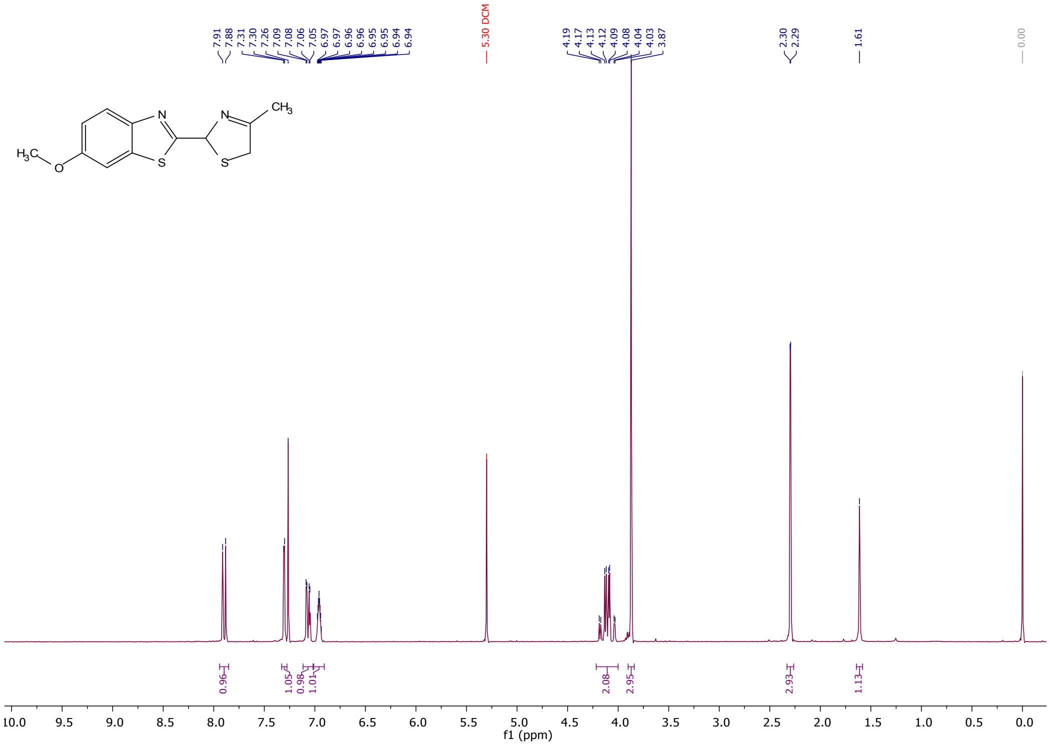

^{1}H NMR Spectra of **7m, 300 MHz (CDCl$_3$)**

7m ^{13}C {^{1}H}

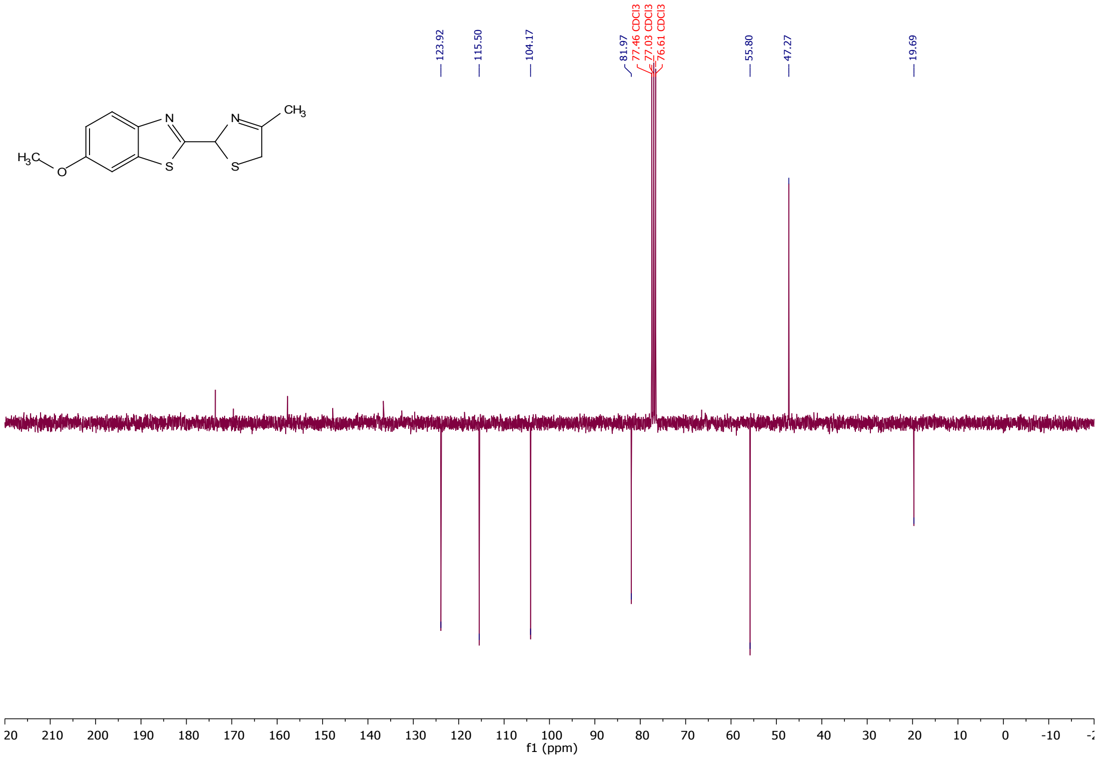

^{13}C {^{1}H} NMR Spectra of **7m,75 MHz (CDCl$_3$)**

7n ^{1}H

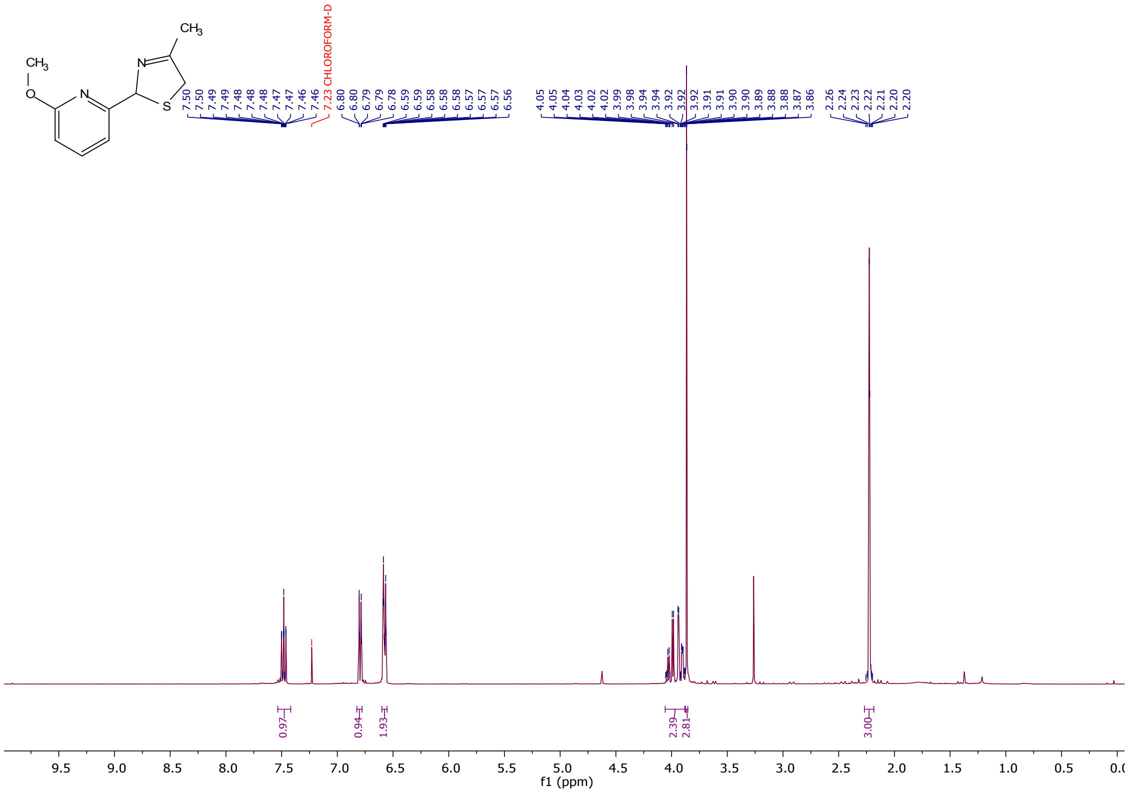

^{1}H NMR Spectra of **7n, 400 MHz (CDCl$_3$)**

7n ^{13}C {^{1}H}

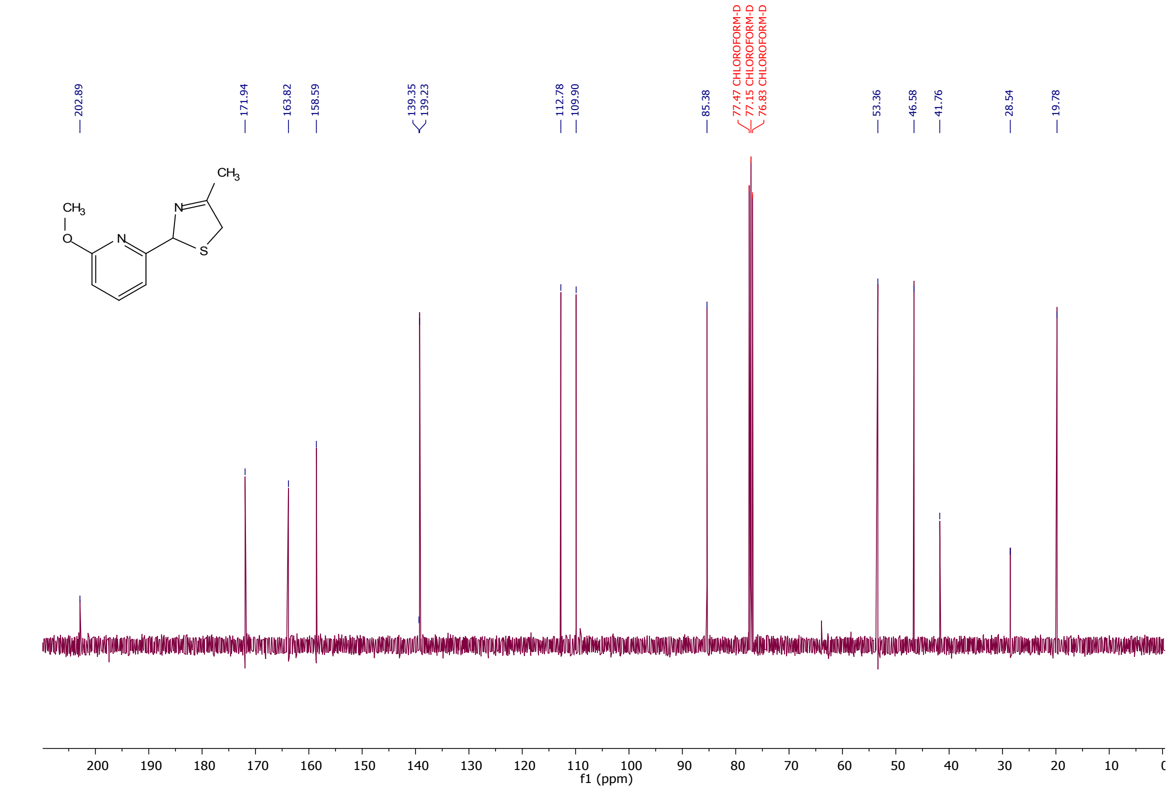

^{13}C {^{1}H} NMR Spectra of **7n,101 MHz (CDCl$_3$)**

7p ^{1}H

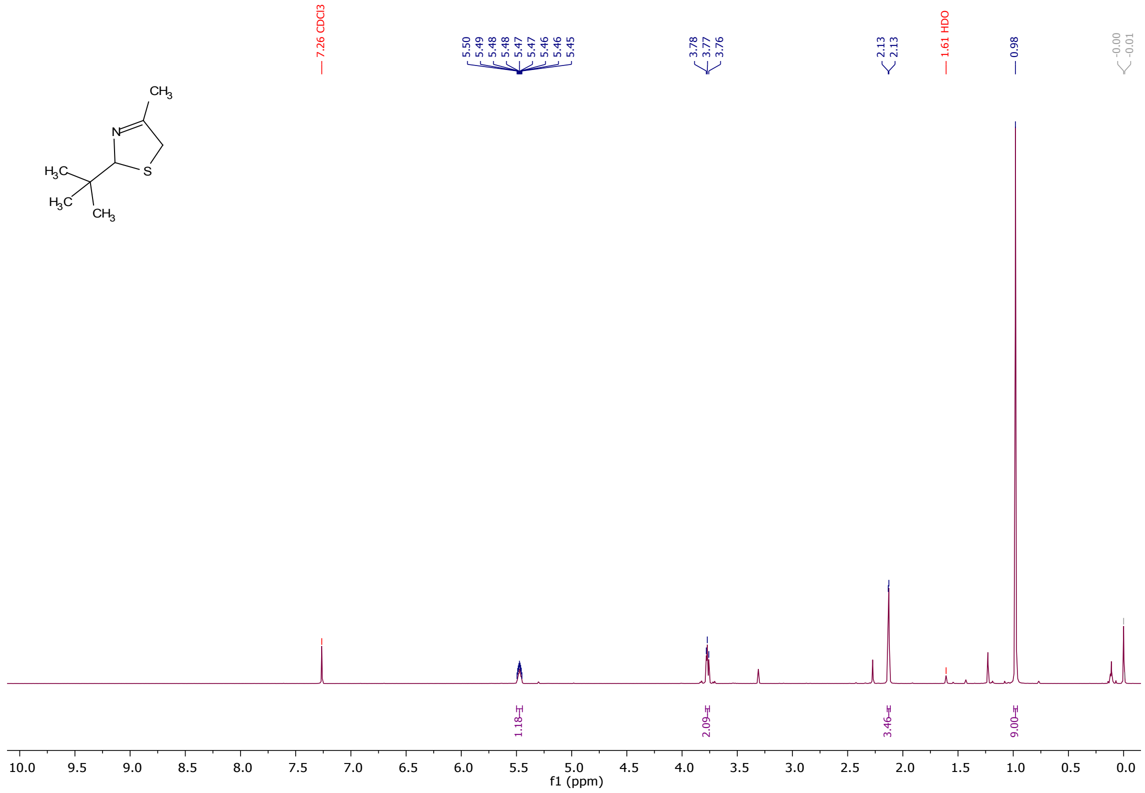

^{1}H NMR Spectra of **7p, 300 MHz (CDCl$_3$)**

7p ^{13}C {^{1}H}

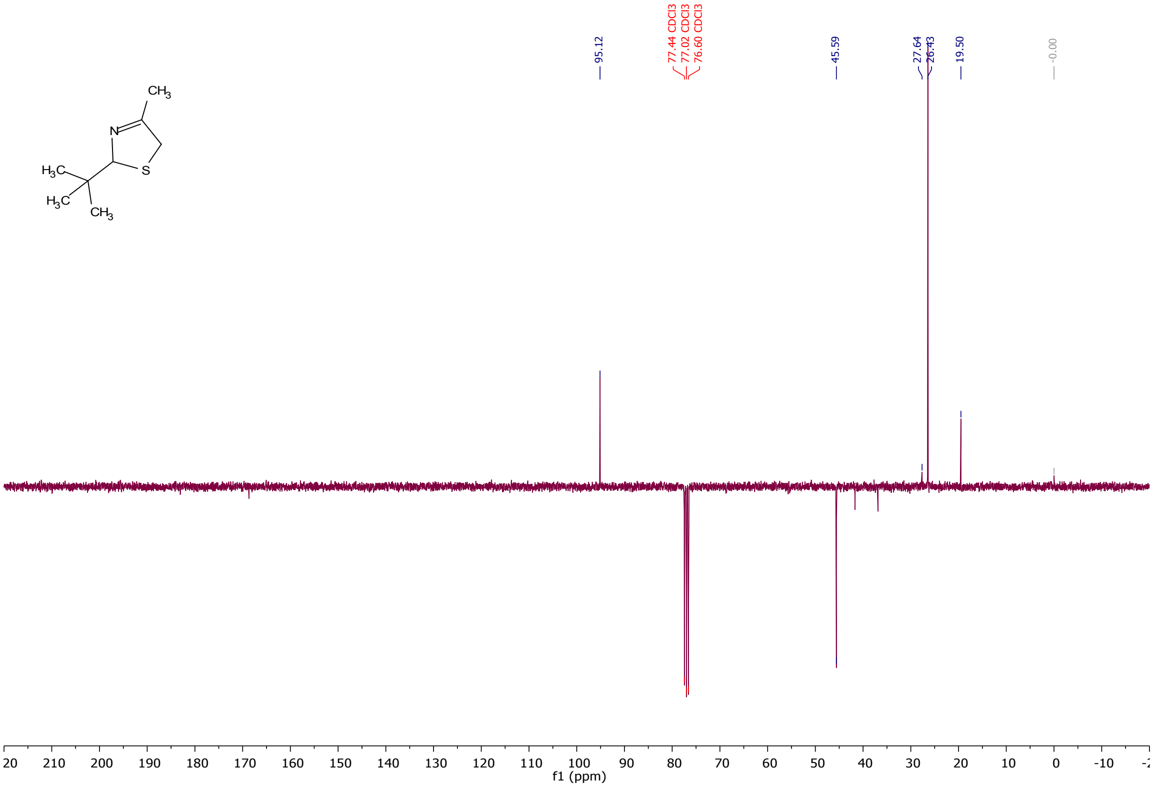

^{13}C {^{1}H} NMR Spectra of **7p,75 MHz (CDCl$_3$)**

7q ^{1}H

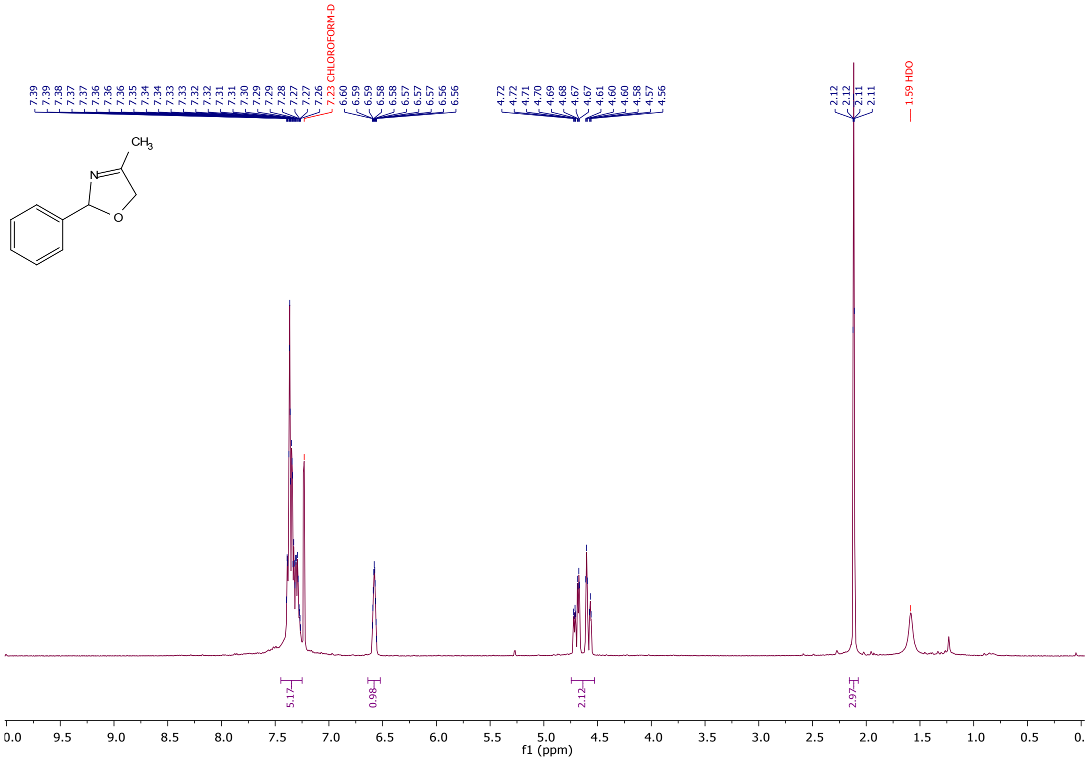

^{1}H NMR Spectra of **7q, 400 MHz (CDCl$_3$)**

7q 13C {1H}

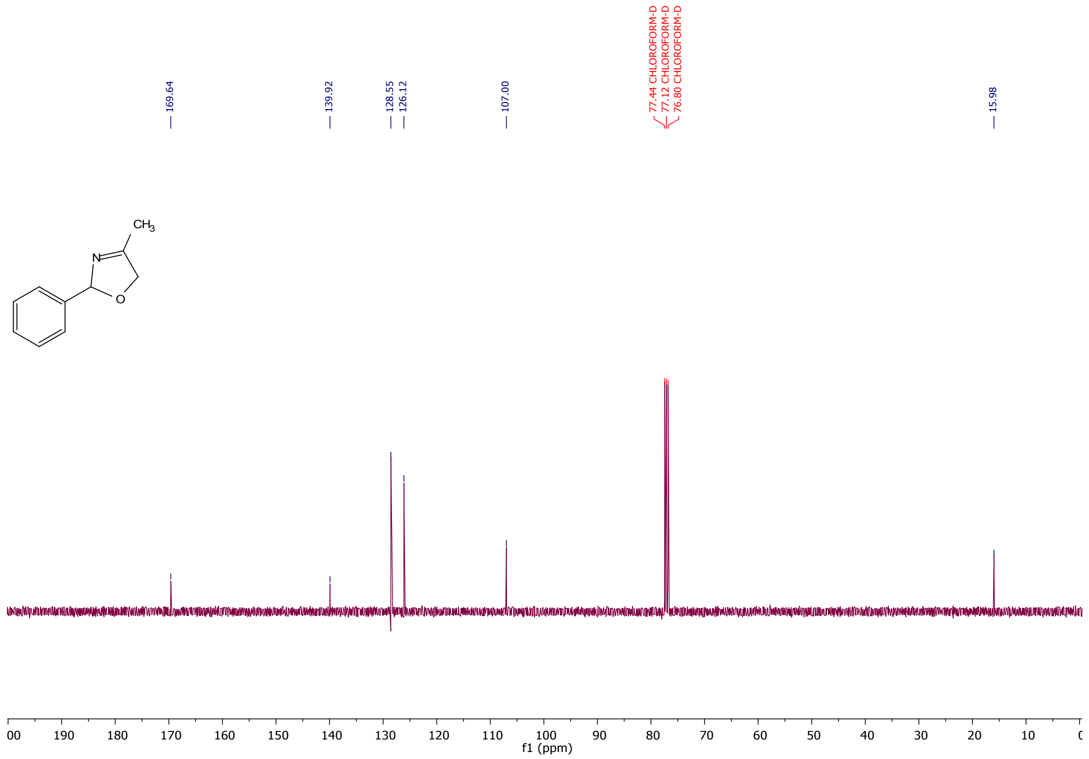

^{13}C{^{1}H} NMR Spectra of **7q, 101 MHz (CDCl$_3$)**

7r ¹H

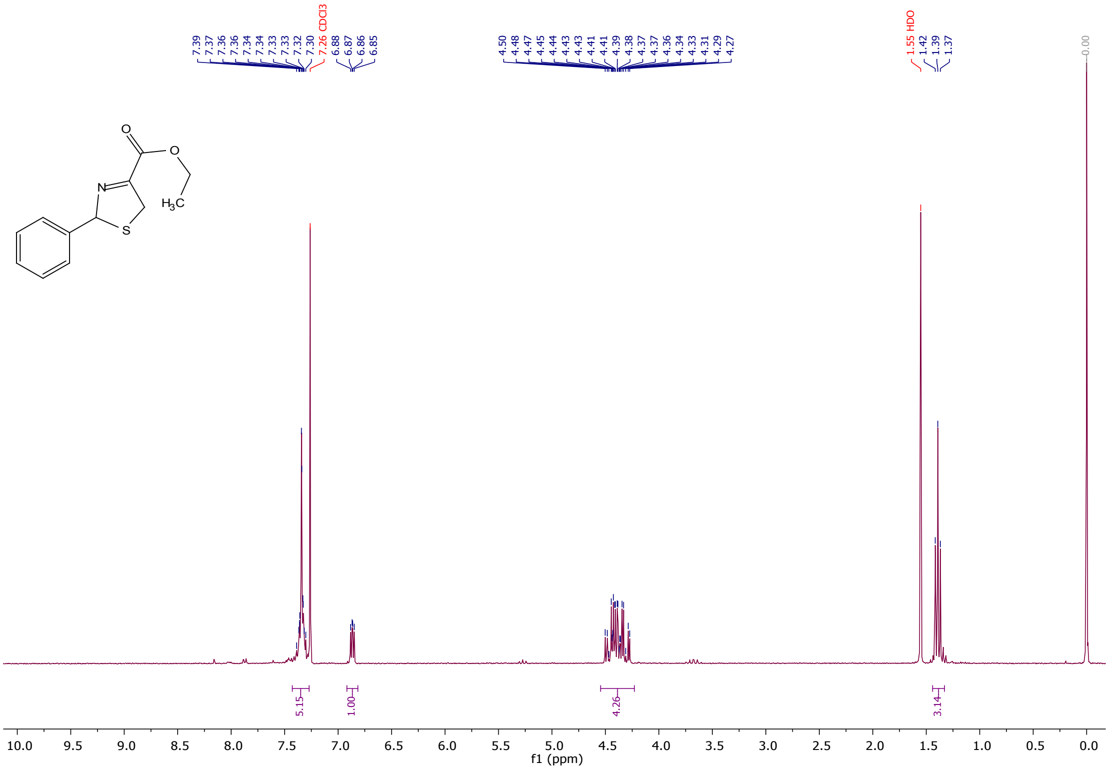

^{1}H NMR Spectra of **7r, 300 MHz (CDCl$_3$)**

7r ¹³C {¹H}

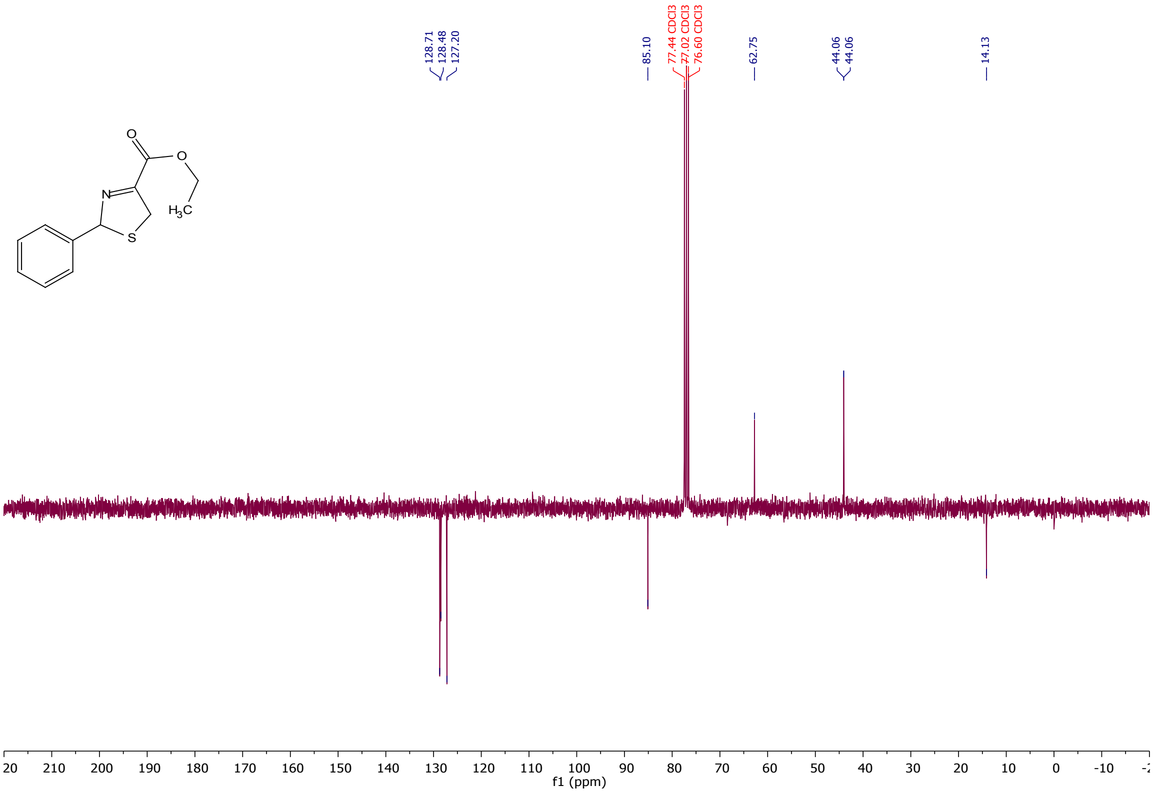

^{13}C {^{1}H} NMR Spectra of **7r,75 MHz ($CDCl_3$)**

7s ^{1}H

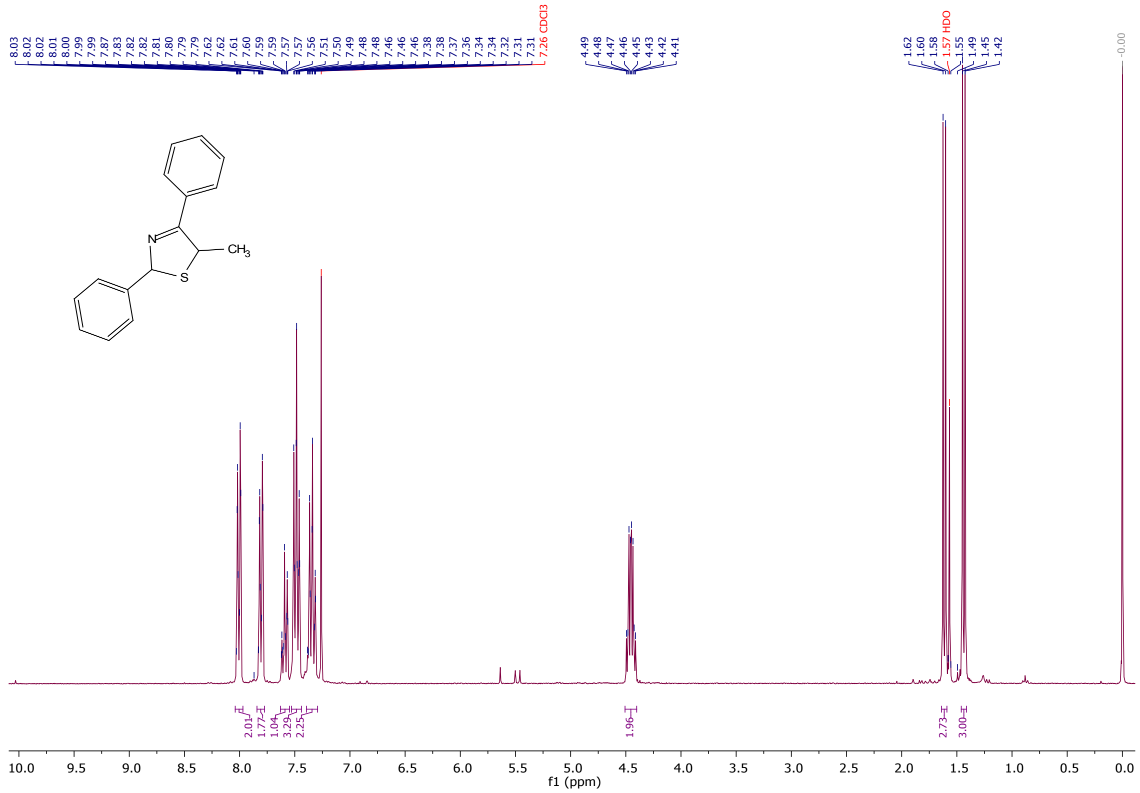

^{1}H NMR Spectra of **7s, 300 MHz ($CDCl_3$)**

7s ^{13}C {^{1}H}

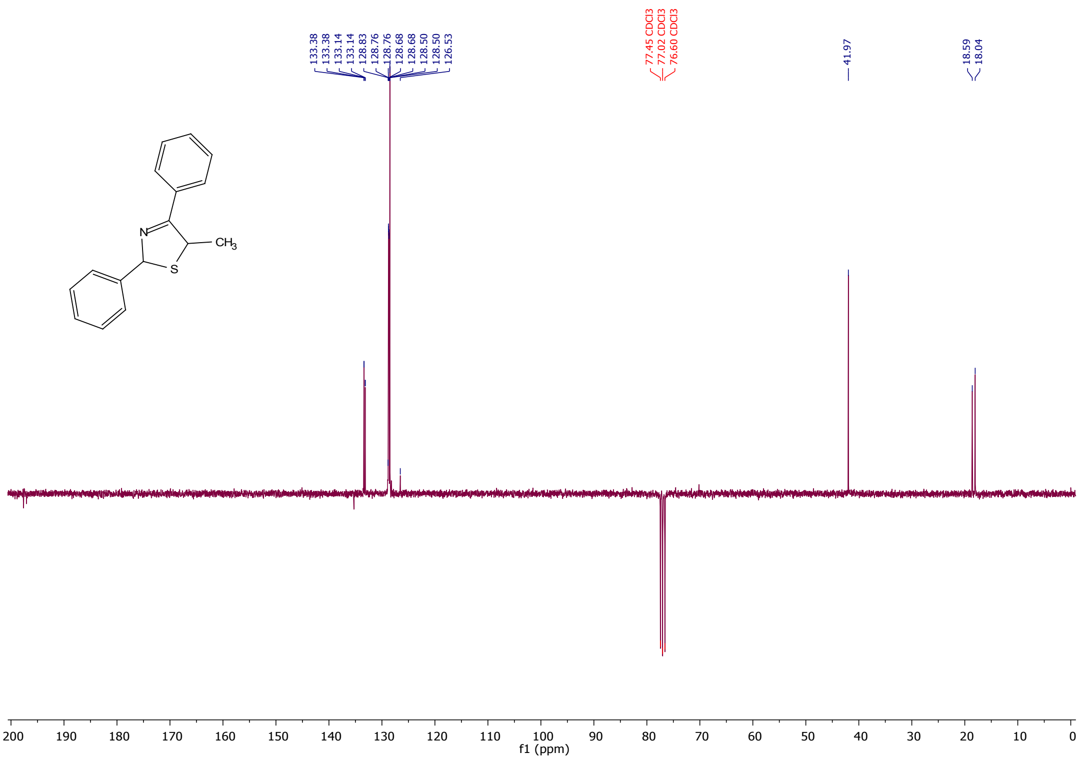

^{13}C {^{1}H} NMR Spectra of **7s,75 MHz (CDCl$_3$)**

7t ^{1}H

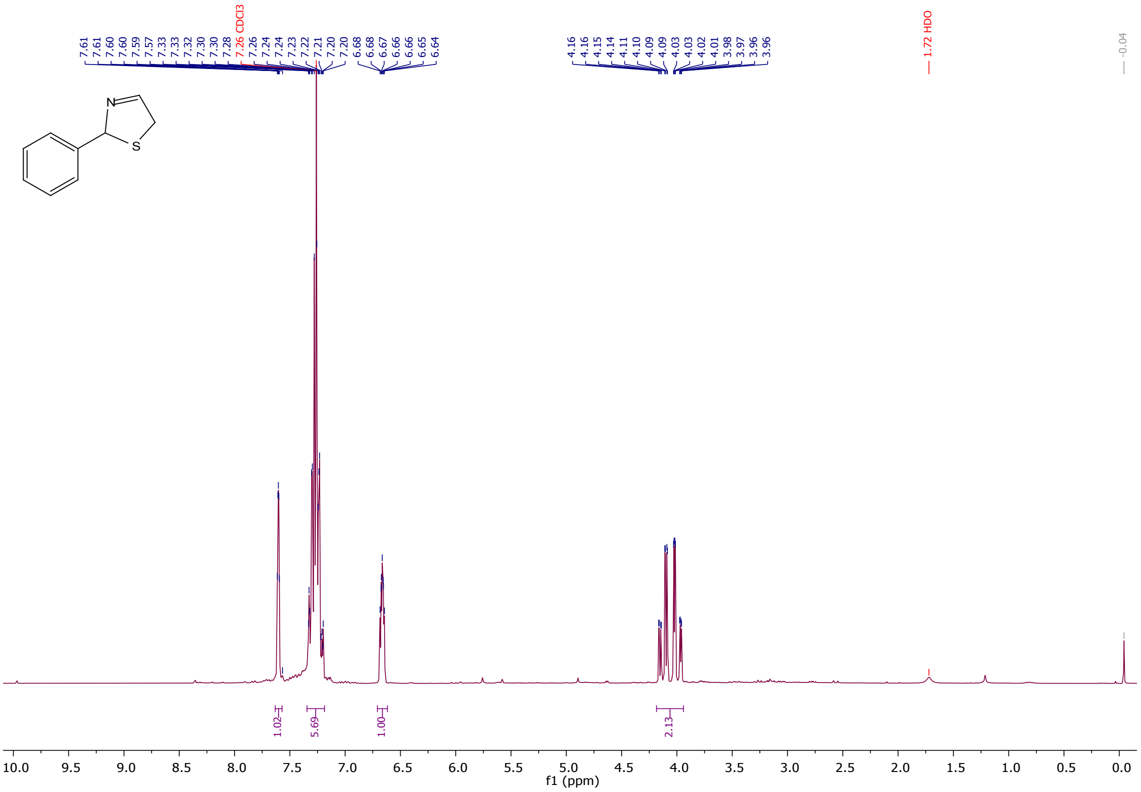

^{1}H NMR Spectra of **7t, 300 MHz (CDCl$_3$)**

7t ^{13}C {^{1}H}

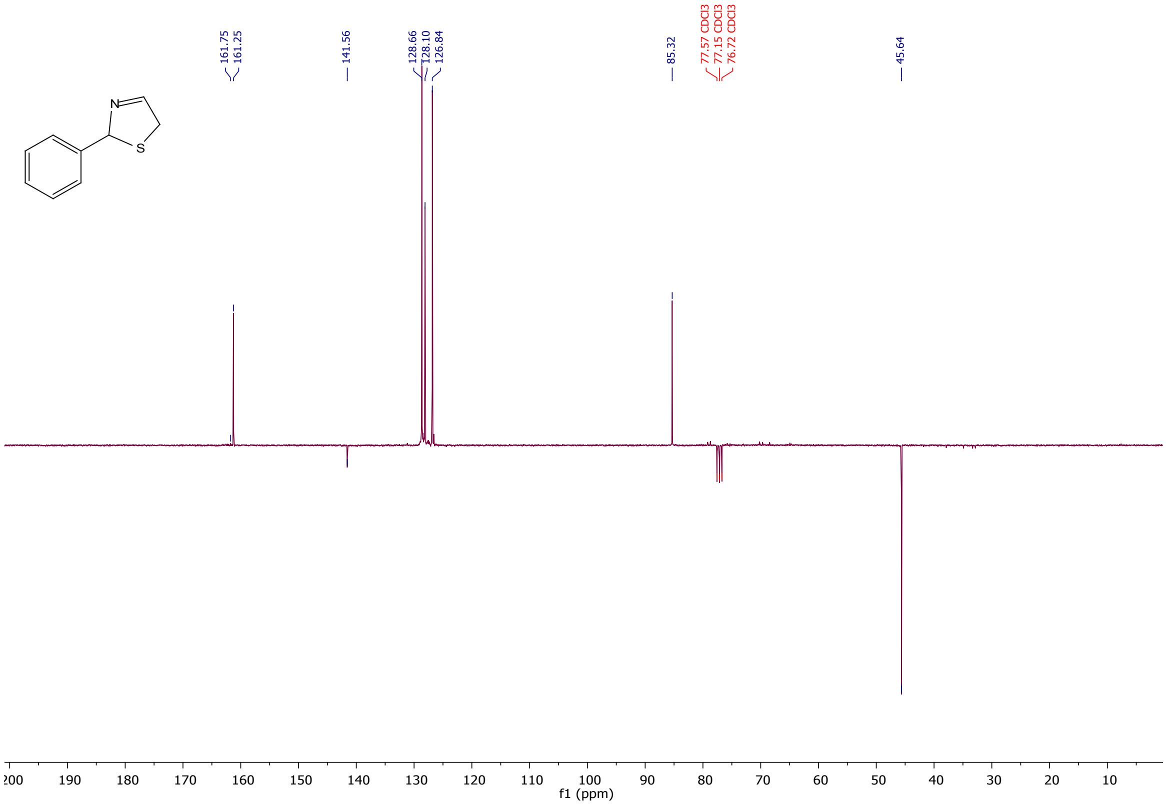

$^{13}C\{^1H\}$ NMR Spectra of **7t,75 MHz (CDCl$_3$)**

8. Spectral data α-keto-thiol, 2-thiazolines, precursors and Luciferin Analogs

6b ¹H

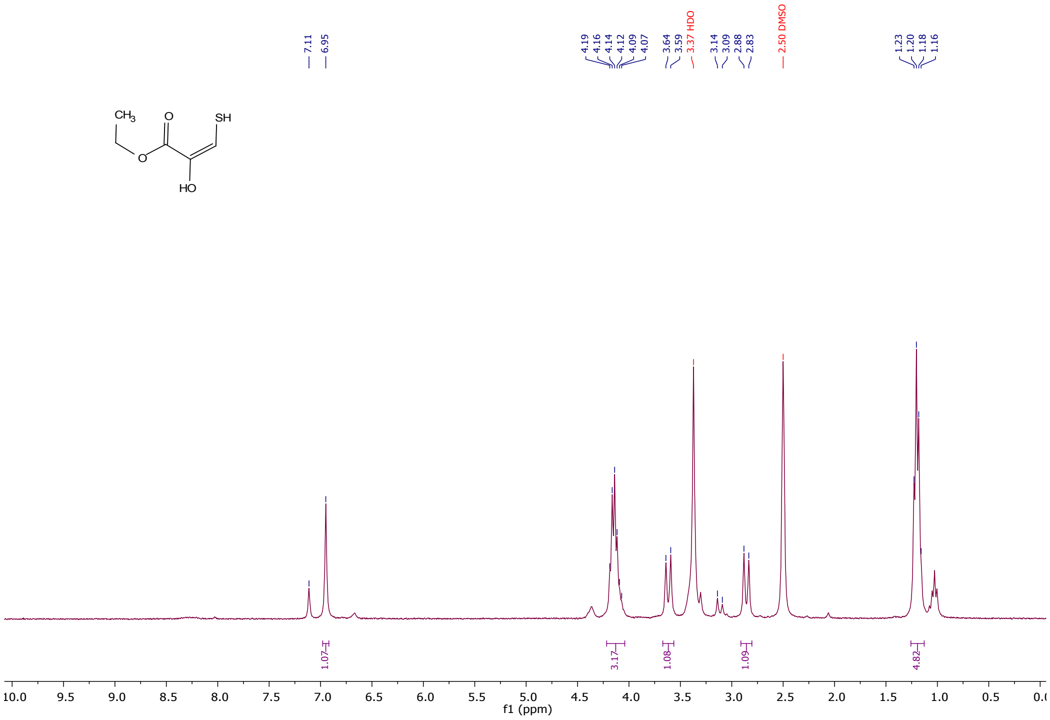

^{1}H NMR Spectra of **6b, 300 MHz (DMSO-d6)**

6b ^{13}C {^{1}H}

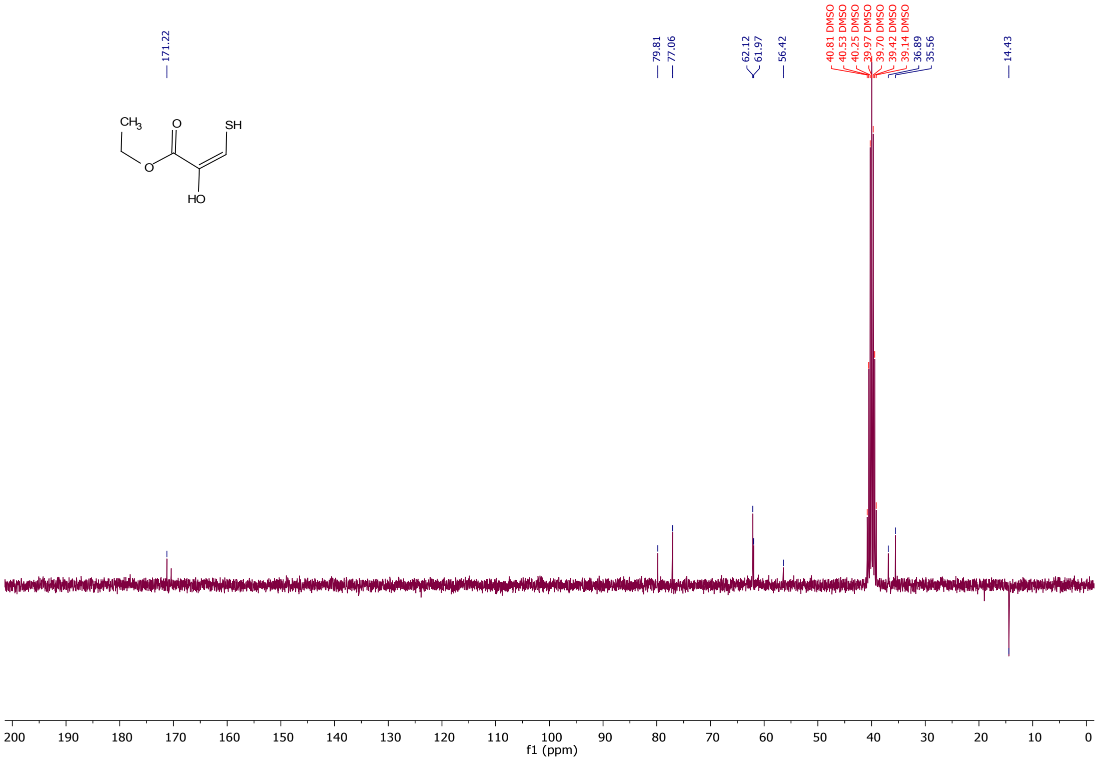

^{13}C {^{1}H} NMR Spectra of **6b,75 MHz (DMSO-d6)**

15 ^{1}H

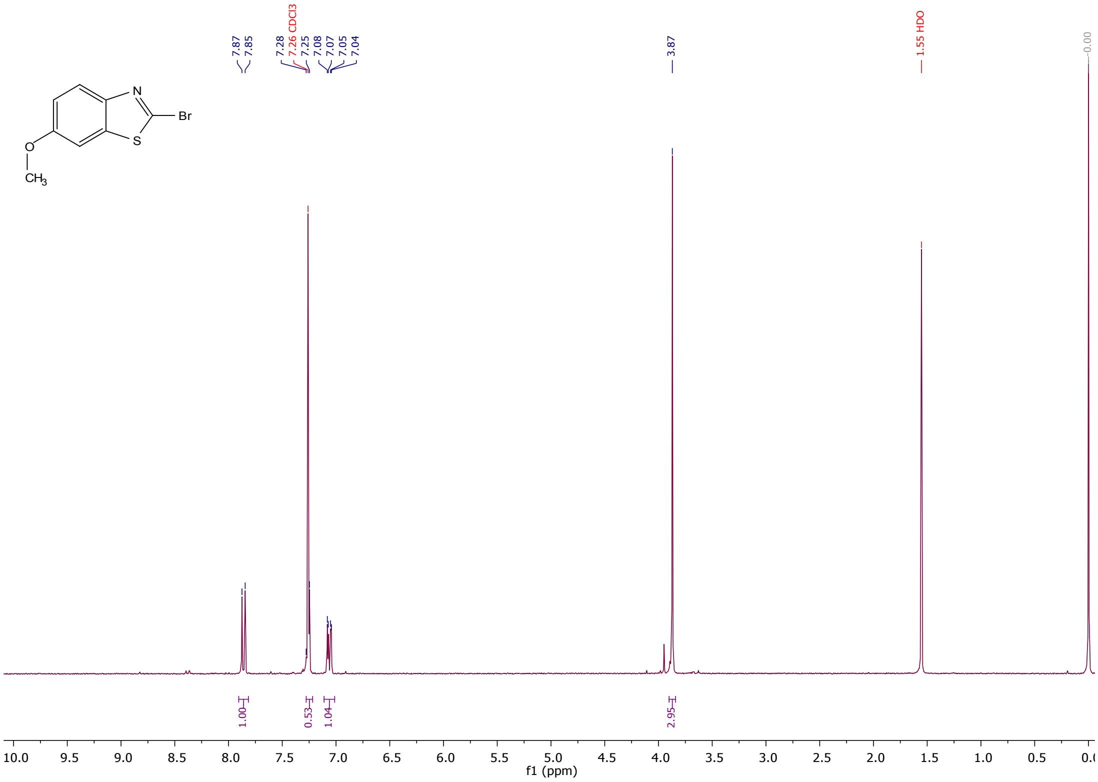

^{1}H NMR Spectra of **15, 300 MHz ($CDCl_3$)**

15 ^{13}C {^{1}H}

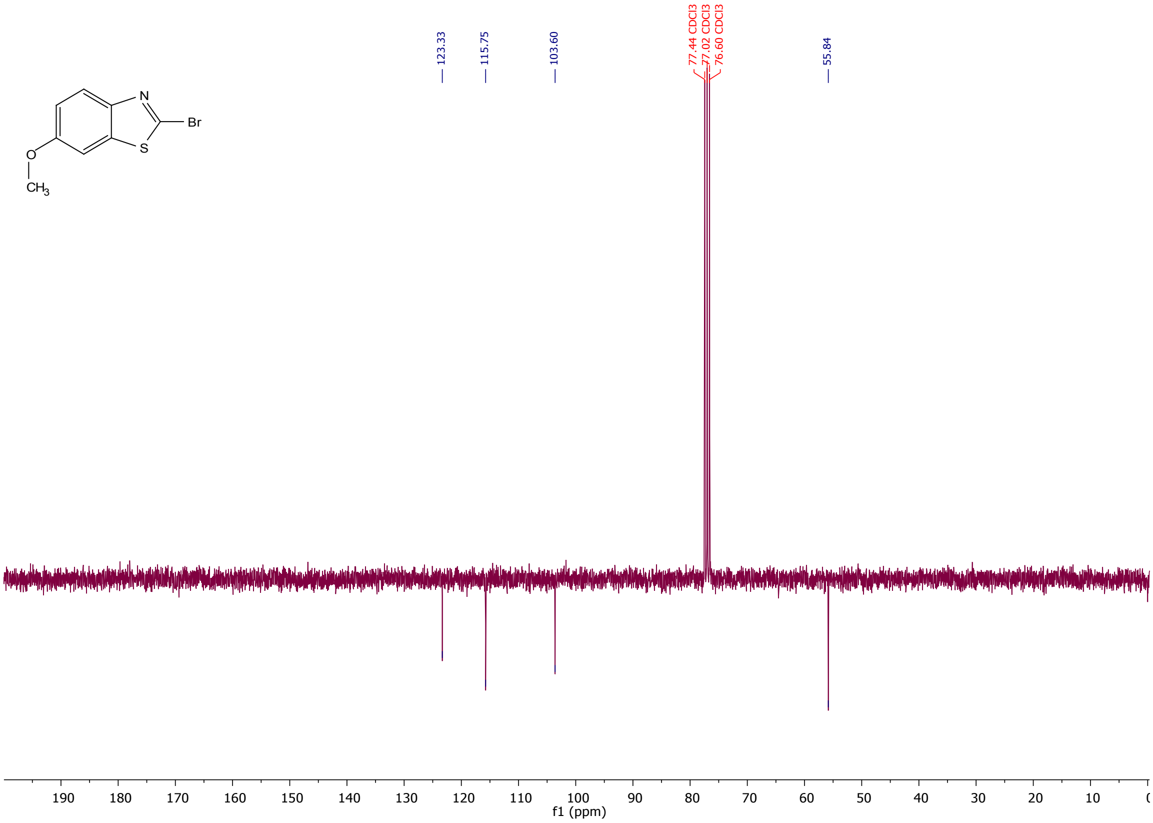

$^{13}C\{^1H\}$ NMR Spectra of **15,75 MHz (CDCl$_3$)**

16 ^1H

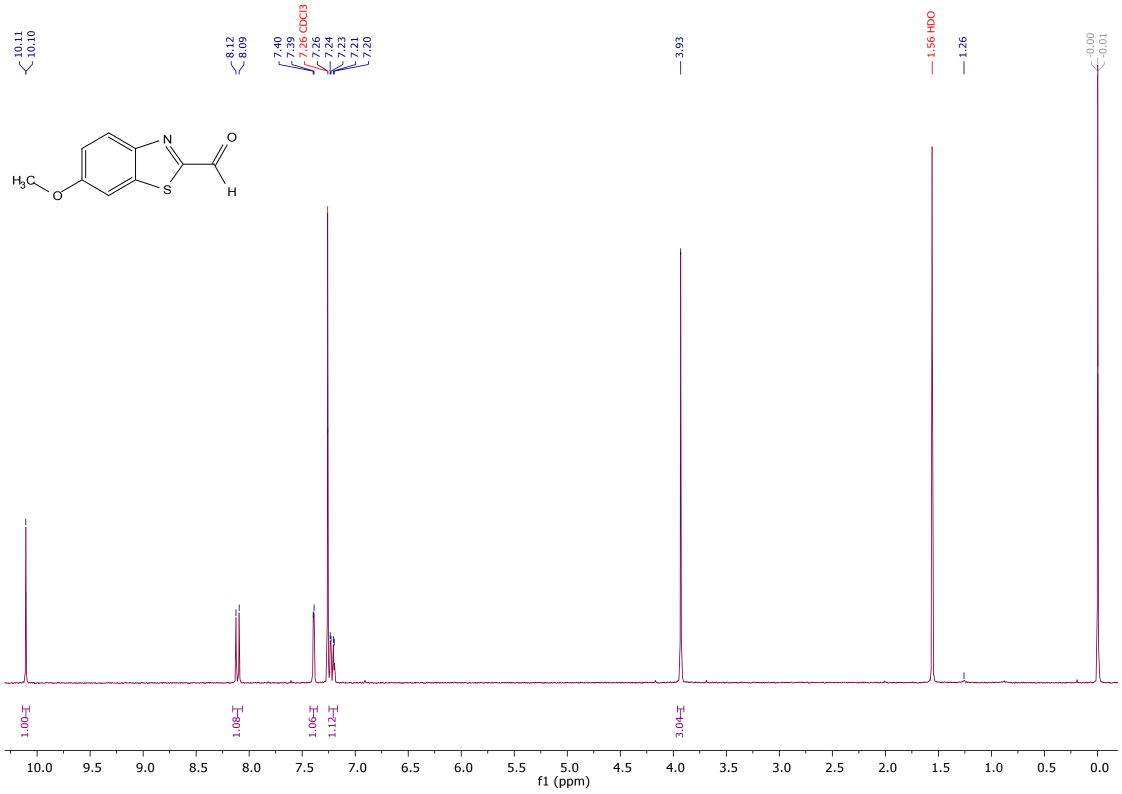

^1H NMR Spectra of **16, 300 MHz (CDCl$_3$)**

16 ${}^{13}C$ {${}^{1}H$}

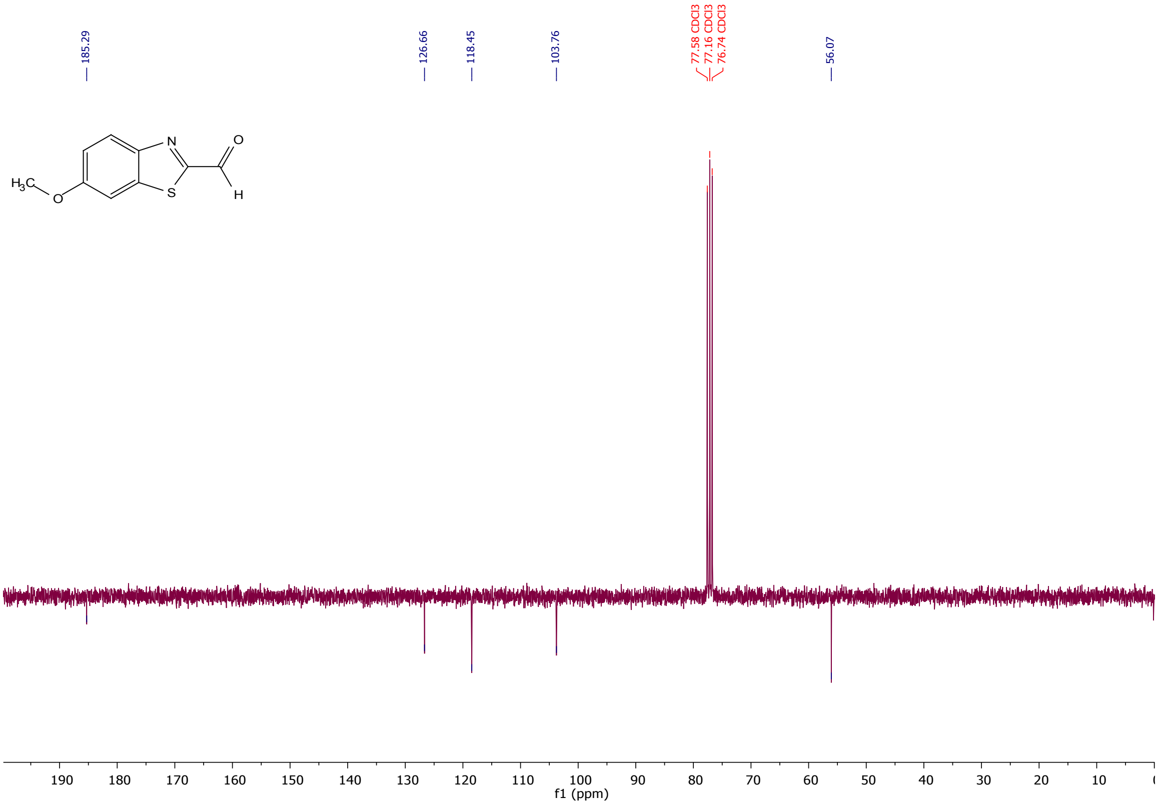

${}^{13}C$ {${}^{1}H$} NMR Spectra of **16,75 MHz (CDCl$_3$)**

10 ${}^{1}H$

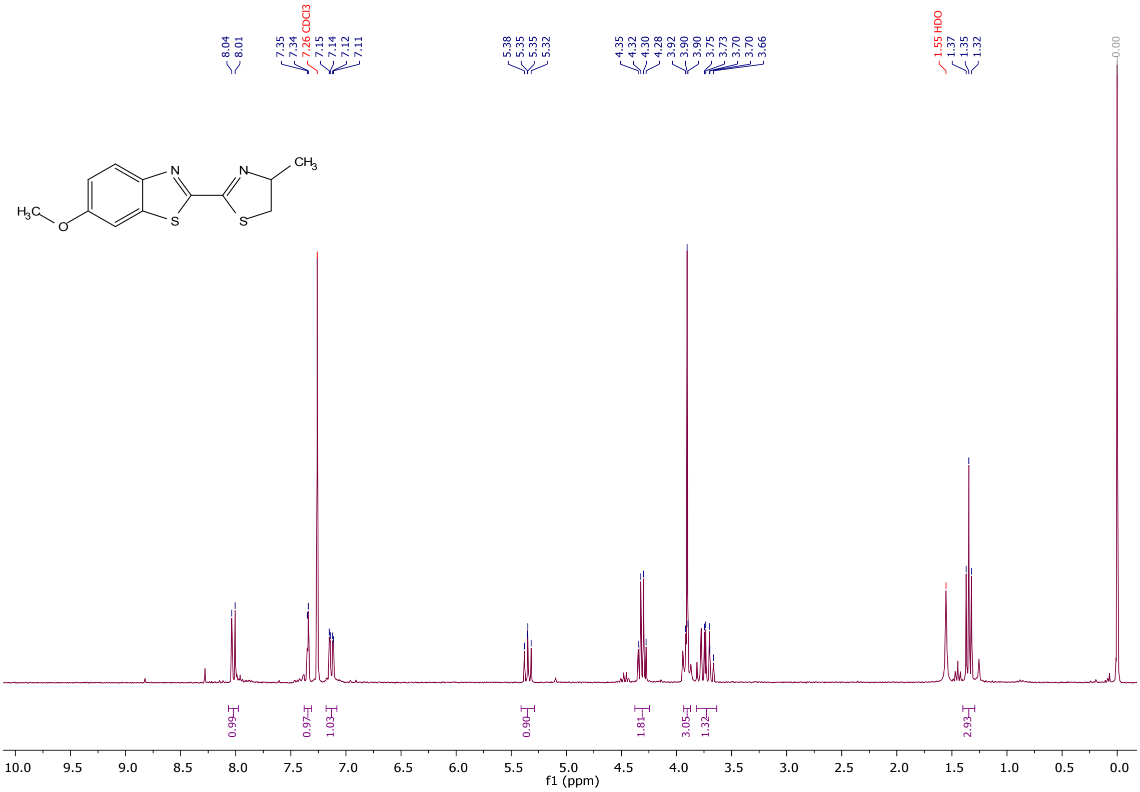

^1H NMR Spectra of **10, 300 MHz (CDCl$_3$)**

10 ^{13}C {^1H}

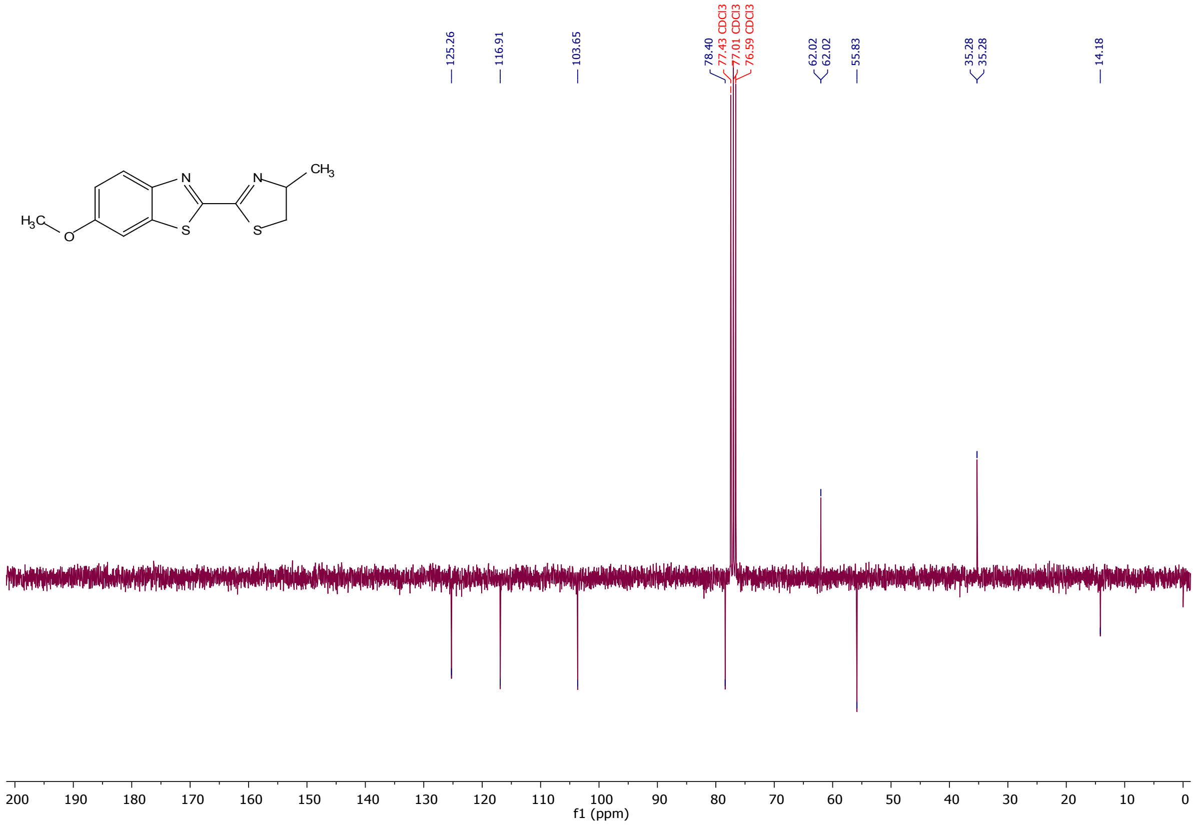

^{13}C {^1H} NMR Spectra of **10,75 MHz (CDCl$_3$)**

11b ^1H

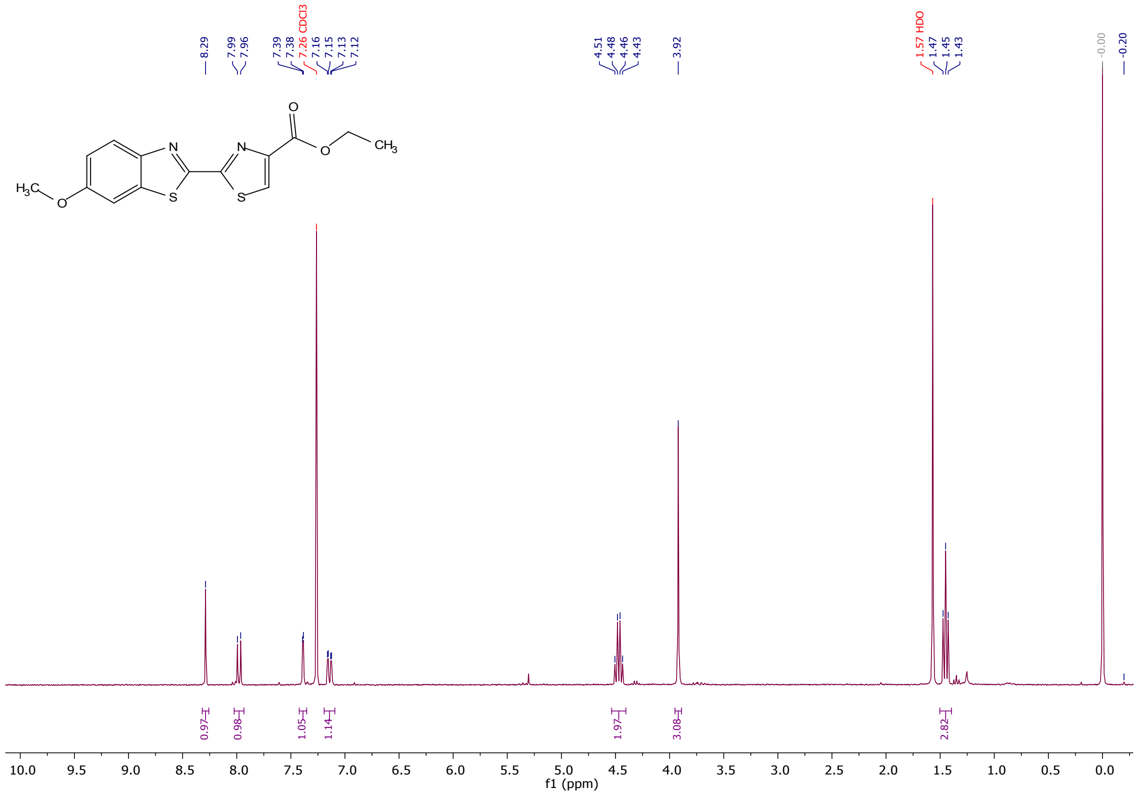

^{1}H NMR Spectra of **11b, 300 MHz (CDCl$_3$)**

11b ^{13}C {^{1}H}

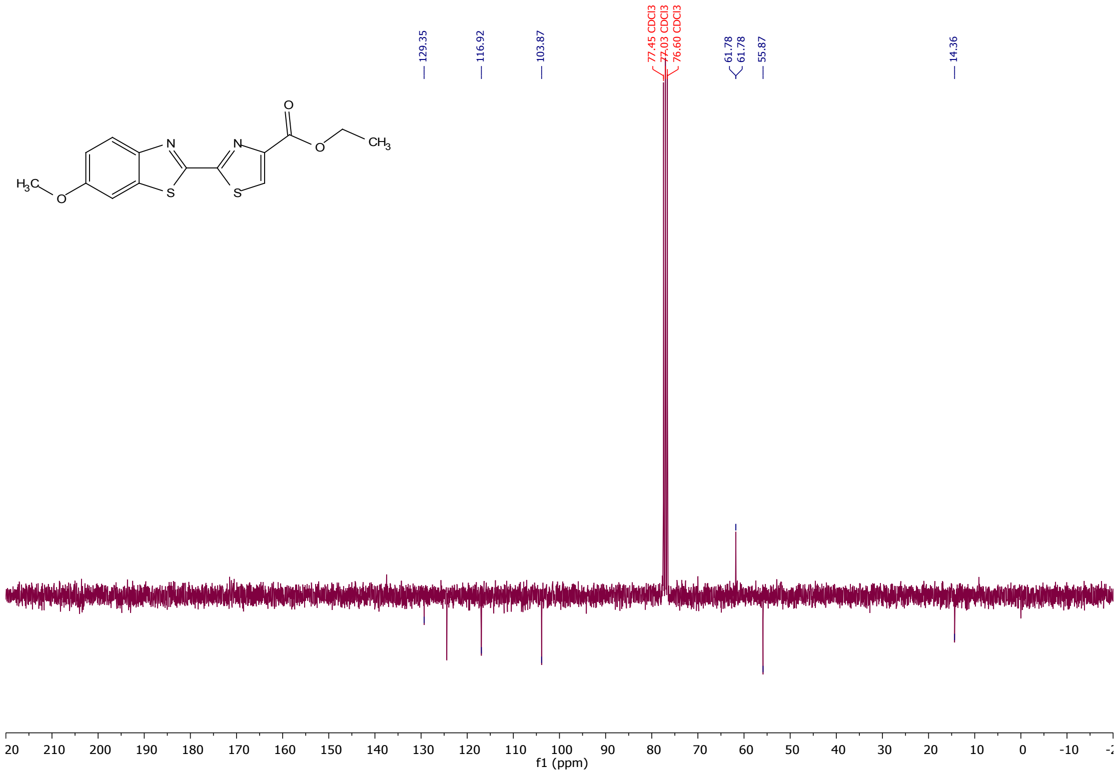

^{13}C {^{1}H} NMR Spectra of **11b,75 MHz (CDCl$_3$)**

12 ^{1}H

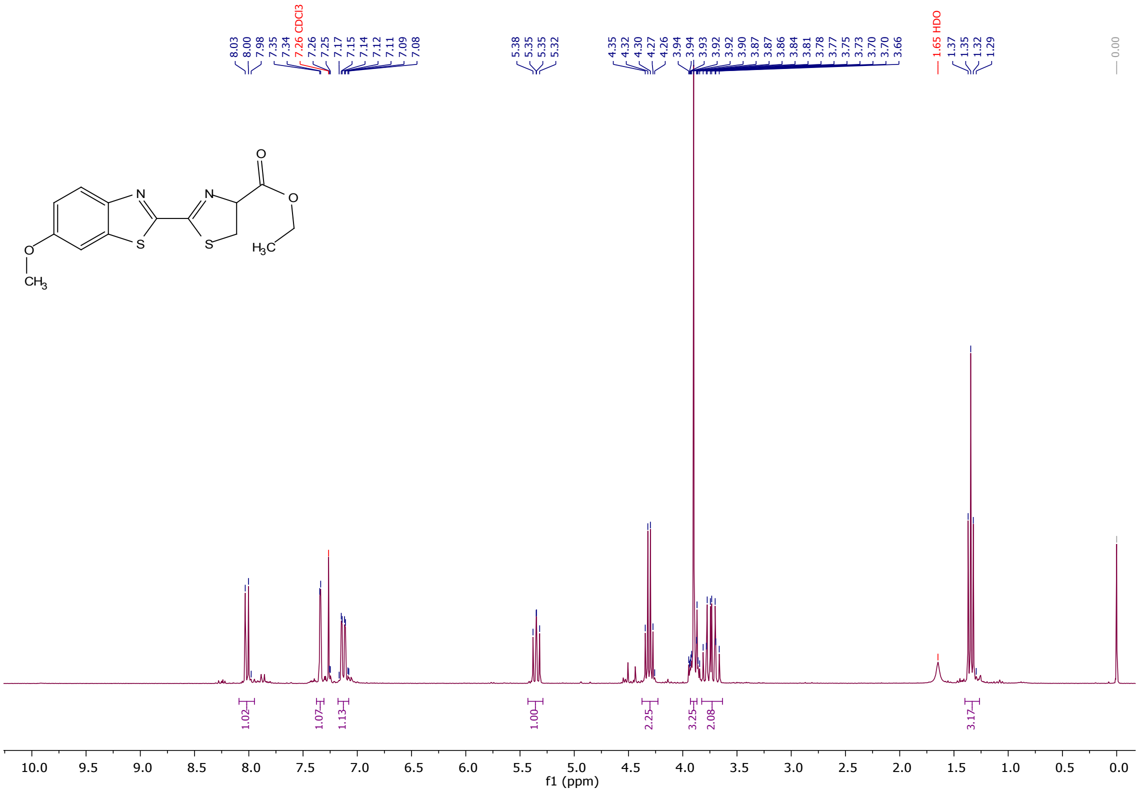

^{1}H NMR Spectra of **11, 300 MHz (CDCl$_3$)**

12 ^{13}C {^{1}H}

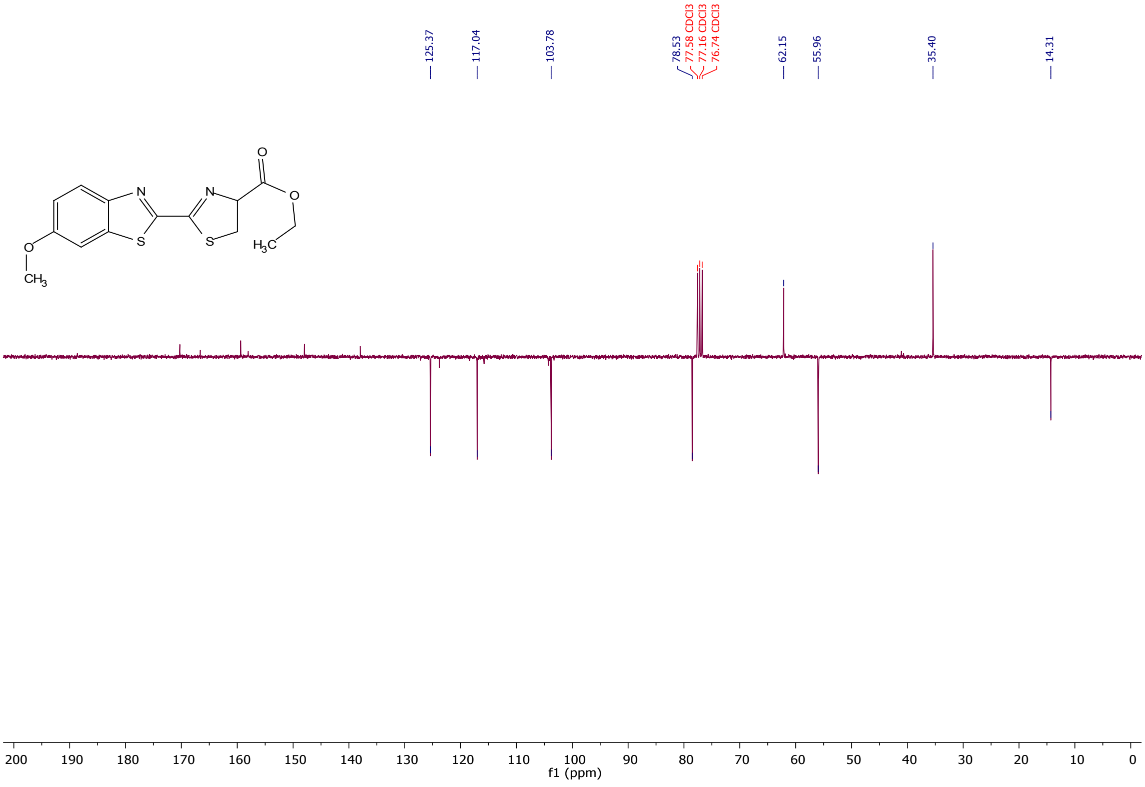

^{13}C {^{1}H} NMR Spectra of **12,75 MHz (CDCl$_3$)**

17 ^{1}H

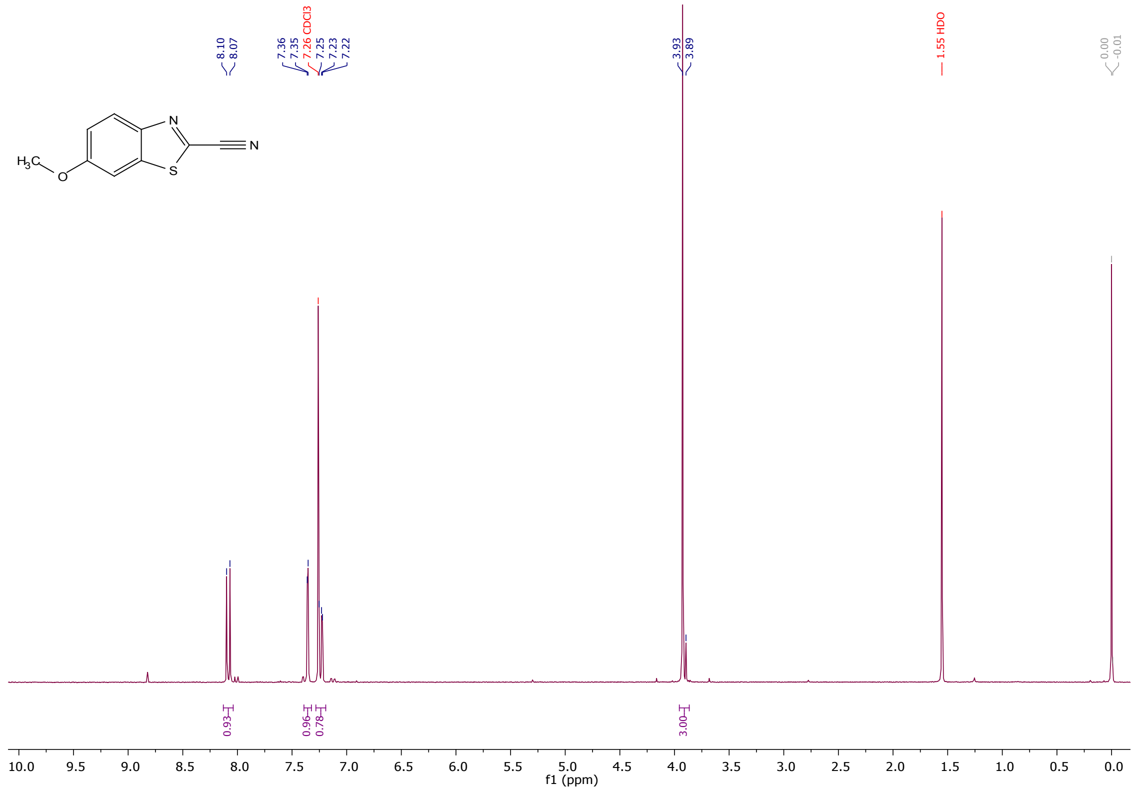

^{1}H NMR Spectra of **17, 300 MHz (CDCl$_3$)**

17 ^{13}C {^{1}H}

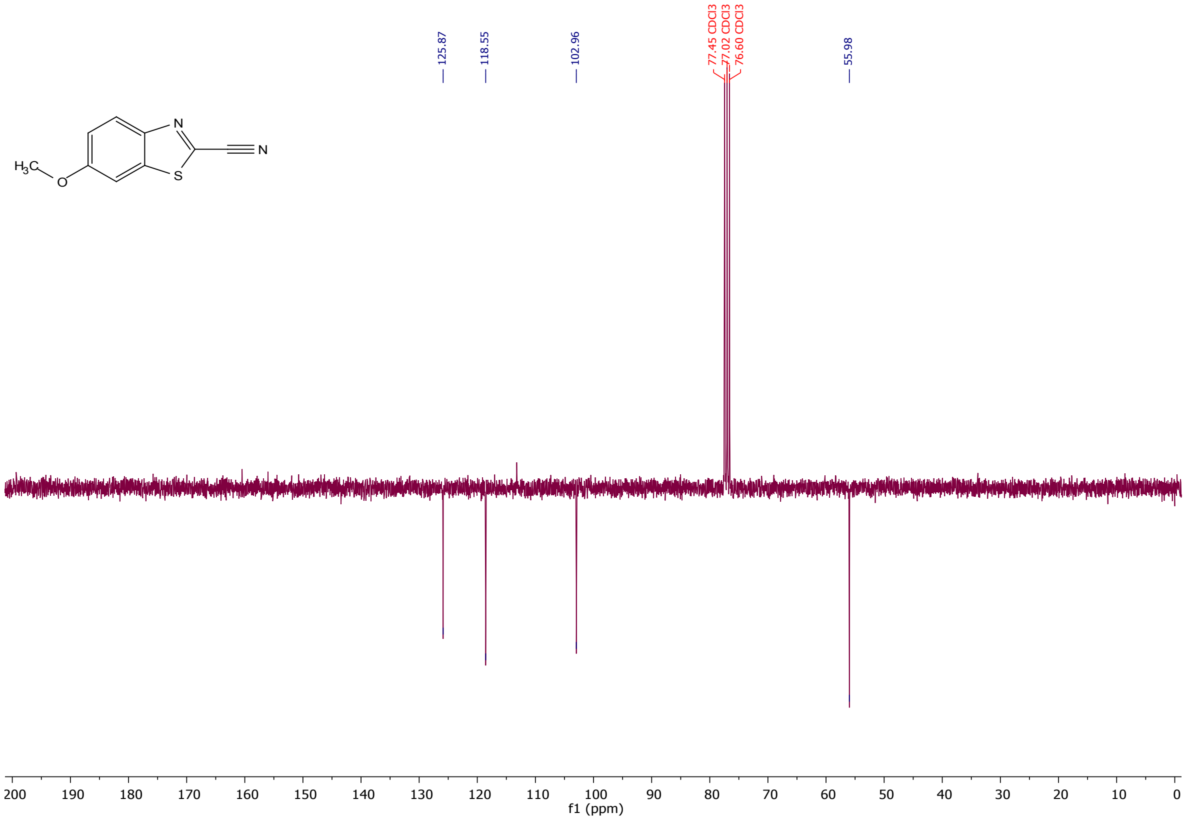

^{13}C {^{1}H} NMR Spectra of **17,75 MHz (CDCl$_3$)**

13 ¹H

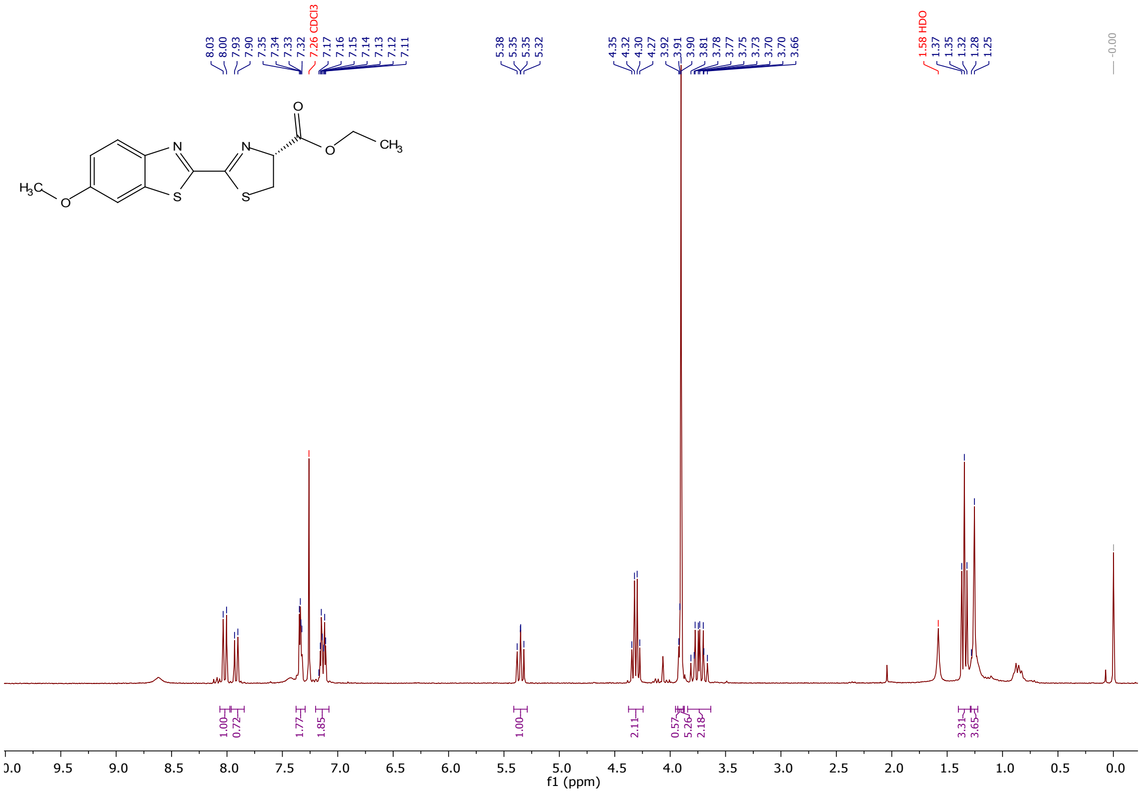

^{1}H NMR Spectra of **13, 300 MHz (CDCl$_3$)**

13 ¹³C {¹H}

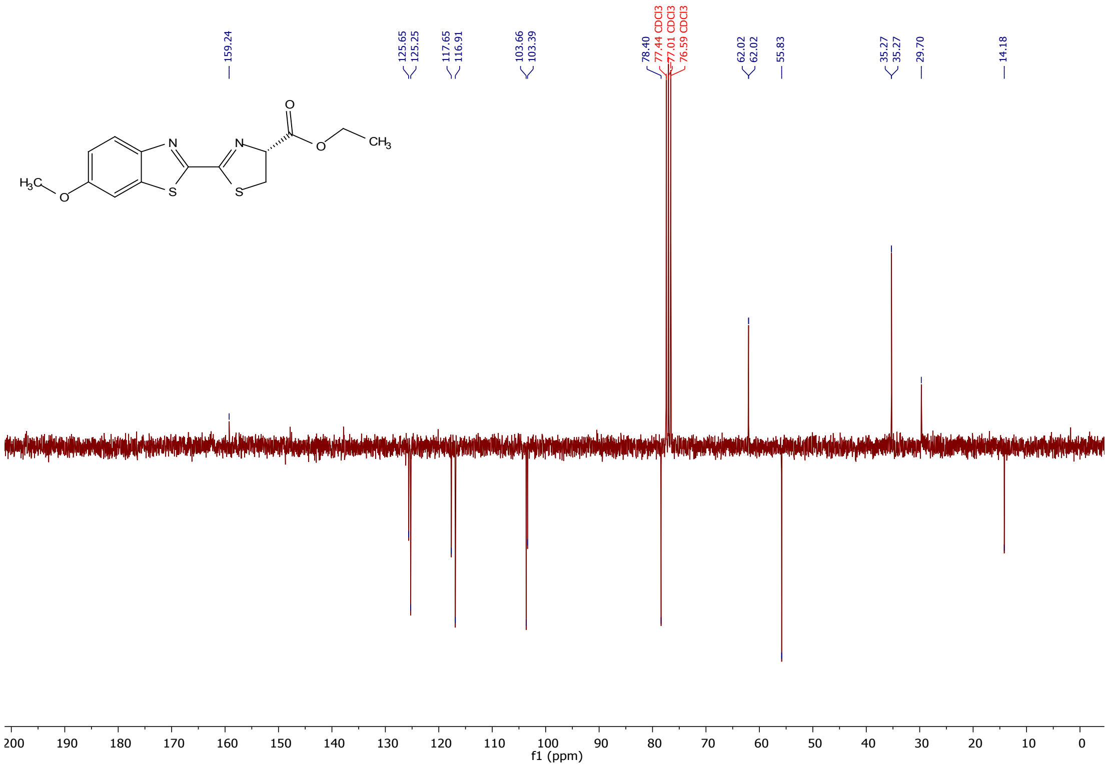

^{13}C {^{1}H} NMR Spectra of **13,75 MHz (CDCl$_3$)**

8 ¹H

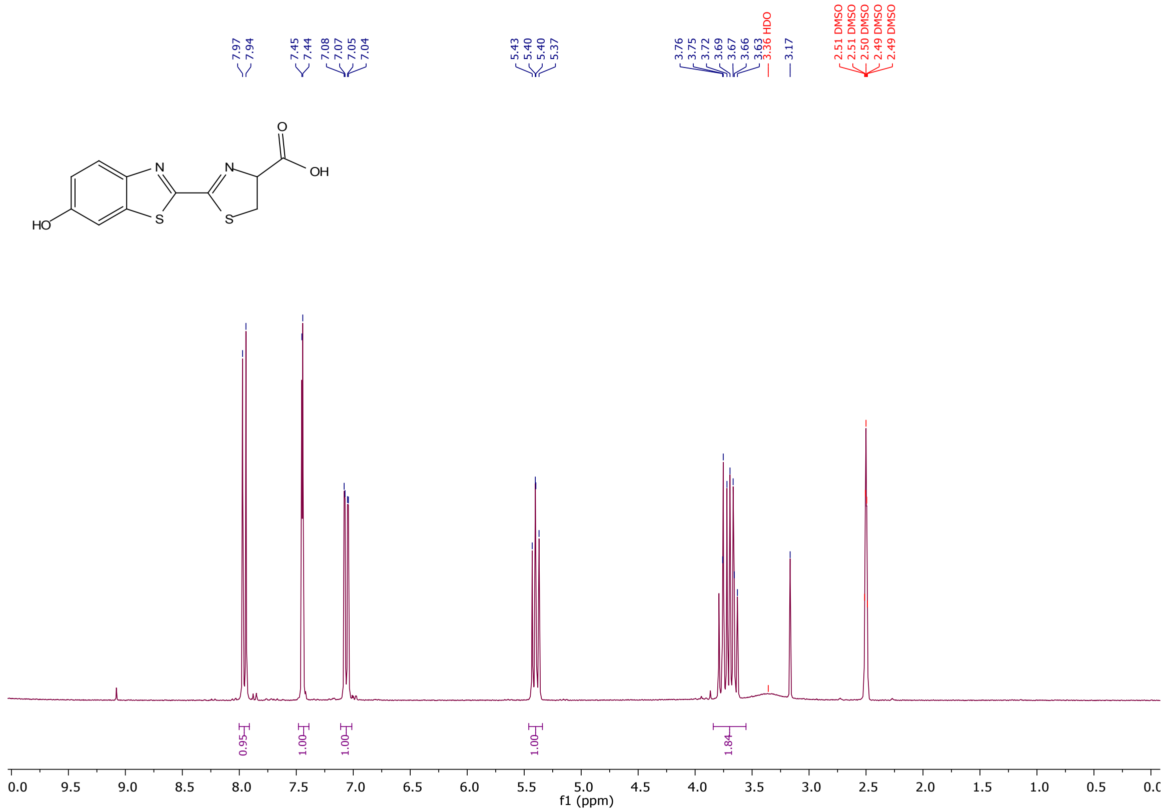

^{1}H NMR Spectra of **8, 300 MHz (DMSO-d6)**

8 ¹³C {¹H}

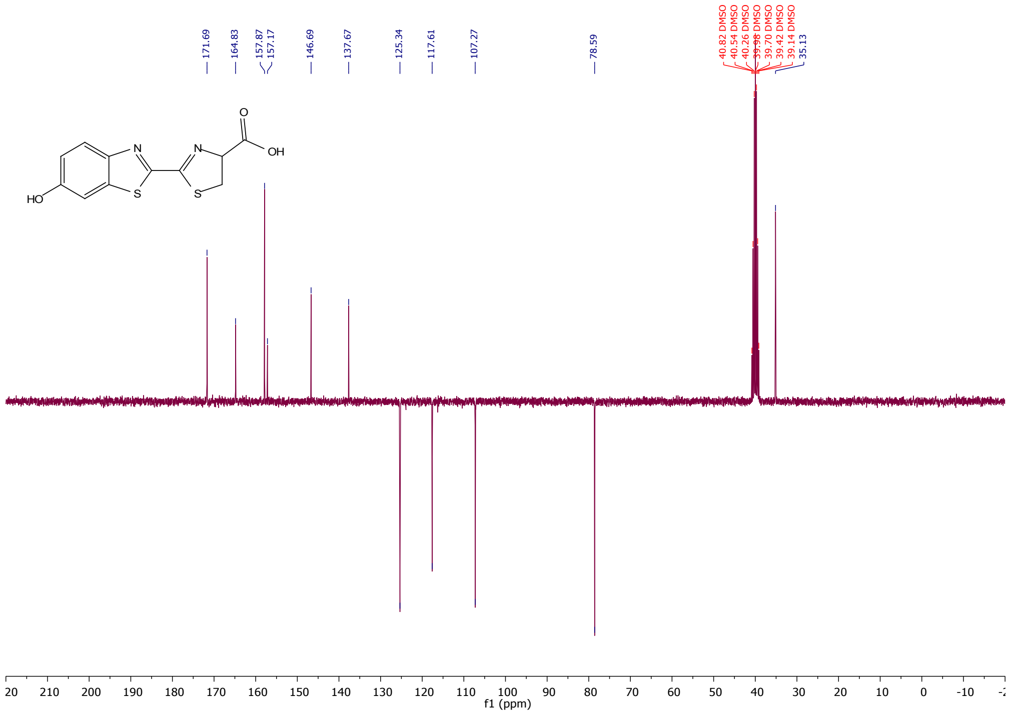

^{13}C {^{1}H} NMR Spectra of **8,75 MHz (DMSO-d6)**

9. Isomerization mechanism of 3-thiazoline (7m) to 2-thiazoline (10)

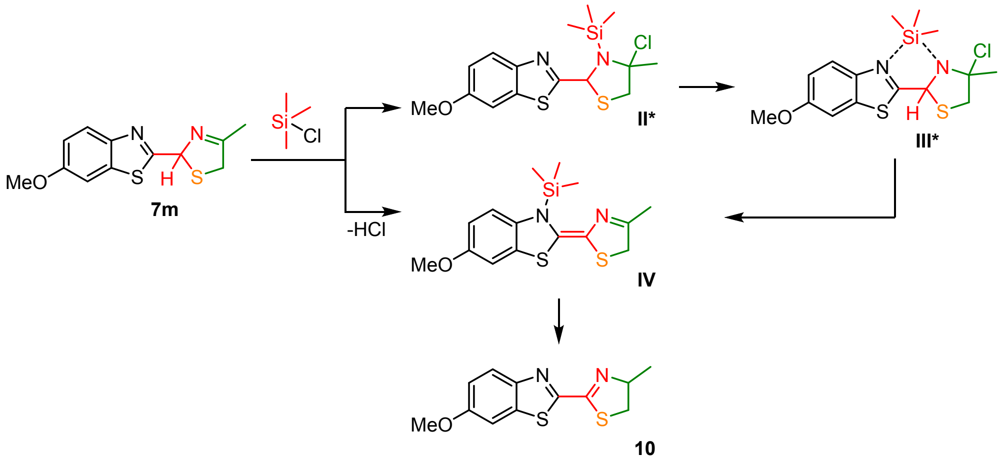

10. References

(1) Fulmer, G. R.; Miller, A. J. M.; Sherden, N. H.; Gottlieb, H. E.; Nudelman, A.; Stoltz, B. M.; Bercaw, J. E.; Goldberg, K. I. NMR Chemical Shifts of Trace Impurities: Common Laboratory Solvents, Organics, and Gases in Deuterated Solvents Relevant to the Organometallic Chemist. *Organometallics* **2010**, *29* (9), 2176–2179. https://doi.org/10.1021/om100106e.

(2) Ojima, I.; Habus, I.; Zhao, M.; Zucco, M.; Park, Y. H.; Sun, C. M.; Brigaud, T. New and Efficient Approaches to the Semisynthesis of Taxol and Its C-13 Side Chain Analogs by Means of β-Lactam Synthon Method. *Tetrahedron* **1992**, *48* (34), 6985–7012. https://doi.org/10.1016/S0040-4020(01)91210-4.

(3) Lou, S.; Moquist, P. N.; Schaus, S. E. Asymmetric Allylboration of Acyl Imines Catalyzed by Chiral Diols. *J. Am. Chem. Soc.* **2007**, *129* (49), 15398–15404. https://doi.org/10.1021/ja075204v.

(4) Suzuki, N.; Nomoto, T.; Toya, Y.; Kanamori, N.; Yoda, B.; Saeki, A. Synthetic Reactions in PEG: PEG-Assisted Synthesis of 2-Cyano-6-Methoxybenzothiazole, A Key Intermediate For The Synthesis of Firefly Luciferin. *Biosci. Biotechnol. Biochem.* **1993**, *57* (9), 1561–1562. https://doi.org/10.1271/bbb.57.1561.

(5) McCutcheon, D. C.; Paley, M. A.; Steinhardt, R. C.; Prescher, J. A. Expedient Synthesis of Electronically Modified Luciferins for Bioluminescence Imaging. *J. Am. Chem. Soc.* **2012**, *134*, 7604–7607. https://doi.org/10.1089/adt.2012.1003.lr.

(6) Bailey, T. S.; Donor, M. T.; Naughton, S. P.; Pluth, M. D. A Simple Bioluminescent Method for Measuring <scp>d</Scp> -Amino Acid Oxidase Activity. *Chem. Commun.* **2015**, *51* (25), 5425–5428. https://doi.org/10.1039/C4CC08145E.
